# Circulating levels of insulin‐like growth factor I (IGF‐I) and risk of multiple myeloma: An observational and Mendelian randomisation study

**DOI:** 10.1111/bjh.70444

**Published:** 2026-03-23

**Authors:** Yolanda Benavente, Sara Hermosa, Nikos Papadimitriou, Alyssa Clay‐Gilmour, Elizabeth E. Brown, Jonathan N. Hofmann, Nathaniel Rothman, Qing Lan, Sonja I. Berndt, Demetrius Albanes, Mark Purdue, Mitchell J. Machiela, Stephen J. Chanock, Parveen Bhatti, Wendy Cozen, Aaron Norman, Susan L. Slager, James R. Cerhan, Vincent Rajkumar, Shaji K. Kumar, Celine M. Vachon, Anne J. Novak, Thomas M. Habermann, Brian K. Link, Gilles Salles, Herve Ghesquieres, Paige M. Bracci, Elizabeth Holly, Rosalie G. Griffin, Michelle A. T. Hildebrandt, Roel C. H. Vermeulen, P. Martijn Kolijn, Henrik Hjalgrim, Karin Ekström Smedby, Harindra Jayasekara, Simon Cheah, Alain Monnereau, Yu Chen, Alan Arslan, Yawei Zhang, Nicola J. Camp, Douglas W. Sborov, Afaf E. G. Osman, Elad Ziv, Immaculata De Vivo, Vijai Joseph, Lauren R. Teras, Alpa V. Patel, Eleanor Kane, Claire M. Vajdic, Adrien Guilloteau, Pierluigi Cocco, Laia Alemany, Juan Sainz, James McKay, Brenda M. Birmann, Delphine Casabonne

**Affiliations:** ^1^ Unit of Infections and Cancer, Cancer Epidemiology Research Programme IDIBELL, Catalan Institute of Oncology L’Hospitalet de Llobregat Spain; ^2^ Centro de Investigación Biomédica en Red de Epidemiología y Salud Pública (CIBERESP) Madrid Spain; ^3^ Department of Public Health, Mental Health and Maternal and Child Health Nursing, Faculty of Nursing Universitat de Barcelona Barcelona Spain; ^4^ Nutrition and Metabolism Branch International Agency for Research on Cancer Lyon France; ^5^ Department of Epidemiology & Biostatistics, Arnold School of Public Health University of South Carolina Columbia South Carolina USA; ^6^ Department of Pathology, Heersink School of Medicine University of Alabama at Birmingham Birmingham Alabama USA; ^7^ Division of Cancer Epidemiology and Genetics, National Cancer Institute National Institutes of Health Bethesda Maryland USA; ^8^ Division of Population Oncology BC Cancer Vancouver British Columbia Canada; ^9^ School of Population and Public Health University of British Columbia Vancouver British Columbia Canada; ^10^ Population Health Sciences BC Cancer Research Institute Vancouver British Columbia Canada; ^11^ Division of Hematology‐Oncology, Department of Medicine University of California Irvine Orange California USA; ^12^ Department of Quantitative Health Sciences Mayo Clinic Rochester Minnesota USA; ^13^ Department of Internal Medicine Mayo Clinic Rochester Minnesota USA; ^14^ Department of Internal Medicine University of Iowa Iowa City Iowa USA; ^15^ Department of Medicine Memorial Sloan Kettering Cancer and Weill Cornell Medical College New York New York USA; ^16^ Department of Hematology Hospices Civils de Lyon, Lyon Sud Hospital Pierre Benite France; ^17^ Department of Epidemiology and Biostatistics UCSF San Francisco California USA; ^18^ Department of Lymphoma/Myeloma, Division of Cancer Medicine The University of Texas MD Anderson Cancer Center Houston Texas USA; ^19^ Institute for Risk Assessment Sciences Utrecht University Utrecht The Netherlands; ^20^ Haematology, Danish Cancer Institute Danish Cancer Society Copenhagen Denmark; ^21^ Department of Haematology Copenhagen University Hospital Rigshospitalet Copenhagen Denmark; ^22^ Department of Clinical Medicine Copenhagen University Copenhagen Denmark; ^23^ Department of Medicine Solna, Division of Clinical Epidemiology Karolinska Institutet Stockholm Sweden; ^24^ Department of Hematology Karolinska University Hospital Stockholm Sweden; ^25^ Cancer Epidemiology Division Cancer Council Victoria Melbourne Victoria Australia; ^26^ Centre for Epidemiology and Biostatistics, School of Population and Global Health University of Melbourne Melbourne Australia; ^27^ School of Public Health and Preventive Medicine, Faculty of Medicine, Nursing and Health Sciences Monash University Clayton Victoria Australia; ^28^ Epidemiology of Childhood and Adolescent Cancers Group, Inserm, Center of Research in Epidemiology and Statistics Sorbonne Paris France; ^29^ Paris Cité (CRESS) Université Paris Descartes Paris France; ^30^ Registre des Hémopathies malignes de la Gironde Institut Bergonié Bordeaux France; ^31^ NYU Grossman School of Medicine New York New York USA; ^32^ Department of Cancer Prevention and Control, National Cancer Center/National Clinical Research Center for Cancer/Cancer Hospital Chinese Academy of Medical Sciences and Peking Union Medical College Beijing China; ^33^ Huntsman Cancer Institute and Division of Hematology and Hematological Malignancies University of Utah School of Medicine Salt Lake City Utah USA; ^34^ Department of Medicine University of California San Francisco Helen Diller Family Comprehensive Cancer Center San Francisco California USA; ^35^ Channing Division of Network Medicine, Department of Medicine Brigham and Women’s Hospital and Harvard Medical School Boston Massachusetts USA; ^36^ Department of Epidemiology, Harvard T.H. Chan School of Public Health Boston Massachusetts USA; ^37^ Niehaus Center for Inherited Cancer Genomics and Department of Medicine, Sloan Kettering Institute Memorial Sloan Kettering Cancer Center New York New York USA; ^38^ Department of Medicine Weill Cornell Medical College New York New York USA; ^39^ Department of Population Science American Cancer Society Atlanta Georgia USA; ^40^ Epidemiology and Cancer Statistics Group, Department of Health Sciences University of York York UK; ^41^ Kirby Institute UNSW Sydney Sydney New South Wales Australia; ^42^ Registre des Hémopathies Malignes de Côte d’Or Dijon France; ^43^ INSERM U1231 CTM, Epi2THM Team Université de Bourgogne Dijon France; ^44^ Centre for Occupational and Environmental Health, Division of Public Health, Health Services Research Primary Care University of Manchester Manchester UK; ^45^ Department of Medical Sciences and Public Health University of Cagliari Cagliari Italy; ^46^ Department of Biochemistry and Molecular Biology, Faculty of Sciences University of Granada (UGR) Granada Spain; ^47^ Genomic Oncology Area, GENYO Centre for Genomics and Oncological Research: Pfizer/University of Granada/Andalusian Regional Government, PTS Granada Spain; ^48^ Instituto de Investigación Biosanitaria IBs.Granada Granada Spain; ^49^ IARC Lyon France

**Keywords:** IGFBP‐3, IGF‐I, lymphoid neoplasms, Mendelian randomisation, multiple myeloma, serum

## Abstract

Evidence for an association between insulin‐like growth factors (IGF) and multiple myeloma (MM) is inconsistent. We examined total IGF‐I concentrations and risk of MM by combining baseline serological data among UK Biobank participants (*n* = 444 187; 732 incident MM) with a two‐sample Mendelian randomisation (MR) analysis using identified genetic variants associated with circulating total IGF‐I and IGF‐binding protein 3 (IGFBP‐3) in the InterLymph consortium (2434 MM and their 2567 controls). Finally, additional lymphoid neoplasm (LN) subtypes were included for comparison with the main hypothesis. Circulating IGF‐I level was positively associated with MM risk Hazard ratio‐HR‐per one standard deviation‐SD‐increase (HR_1‐SD_ = 1.11, 95% confidence interval [CI]: 1.01–1.22; *p*‐value = 0.03), especially closer to diagnosis. Genetically inferred IGF‐I levels were associated with increased MM risk (odds ratio [OR] = 1.27, 95% CI: 1.05–1.54) but not with any other LNs. Genetically inferred IGFBP‐3 levels showed no associations with any LN evaluated. Corroborating previous findings, in a secondary analysis, IGF‐I levels were associated with the risk of chronic lymphocytic leukaemia/small lymphocytic lymphoma (CLL/SLL) in males with higher body mass index (HR_1‐SD in obese male_ = 1.36, 95% CI: 1.14–1.61). Our serological and MR analyses suggest a contributing role of IGF‐I in the susceptibility of MM; the CLL/SLL findings warrant further investigation considering sex‐specific adiposity.

## INTRODUCTION

The insulin‐like growth factor (IGF) system, composed of two polypeptide hormones IGF‐I, IGF‐II, their receptors (IGF‐IR and ‐IIR) and six IGF‐binding proteins (IGFBP‐1 to ‐6), is associated with cell growth, survival, apoptosis, angiogenesis, metabolism and migration.^1^, ^2^ Circulating IGF‐I is associated with the risk of several solid cancers (colorectal, breast and prostate) in observational studies with further support from Mendelian randomisation (MR) studies.^3^, ^4^, ^5^, ^6^, ^7^, ^8^ Evidence is accumulating that the IGF system may contribute to the biology of multiple myeloma (MM).^9^ Borderline non‐significant positive associations were observed for IGF‐I and IGFBP‐3 levels with MM in a nested case–control study within the Multiple Myeloma Cohort Consortium (MMCC).^10^ In the European Prospective Investigation into Cancer and Nutrition (EPIC) study sample, IGF‐I levels were not associated with lymphoid neoplasm (LN) or associated subtypes.^11^ A study examining the risk of 30 cancers and IGF‐I concentrations conducted within the UK Biobank but reported a weak positive association with MM that failed to meet correction for multiple testing.^7^ The recent update of cancer data in the UK Biobank, which shows a doubling of most types of cases, allows for more detailed subtype‐specific investigations to advance aetiological understanding.

Here, we first aimed to estimate if pre‐diagnostic serum levels of IGF‐I are related to MM risk in the UK Biobank study. Then, using a two‐sample MR approach, we obtained estimates that combine the potential effect of genetic variants associated with IGF‐I and IGFBP‐3 levels and estimate the effect of these variants on the risk of MM using genome‐wide association study (GWAS) data from the InterLymph consortium.^12^ We also included additional LN subtypes for comparison. This allowed us to assess whether the observed associations are specific to MM or reflect broader patterns across related LN, thereby strengthening the interpretation and robustness of our findings. Finally, motivated by previous findings linking obesity to MM^13^ and chronic lymphocytic leukaemia/small lymphocytic lymphoma (CLL/SLL)^14^ we examined the role of body mass index (BMI) for these two subtypes.

## METHODS

### The UK Biobank study

#### Observational study

The UK Biobank is a prospective cohort of nearly half a million adults recruited between 2006 and 2010 and aged 40–69 years at enrolment.^15^, ^16^ Further information can be found in Supplementary Methodology S1. Participants with a diagnosis of cancer before recruitment other than non‐melanoma skin cancer (ICD‐10 C44) (*n* = 24 699) and those lacking an IGF‐I measurement (*n* = 33 247) were excluded from the present analysis (Supplementary Methodology S1). Our analysis thus included 444 187 participants (Number 52433).

#### Assessment of outcome

Incident cancer cases within the UK Biobank cohort were identified through linkage to national cancer registries. Cancer incidence data were coded using the 10th Revision of the International Classification of Diseases (ICD‐10) or the 3rd Revision of the ICD‐Oncology (https://biobank.ndph.ox.ac.uk/showcase/ukb/docs/CancerLinkage.pdf). We defined the following first registered end‐points for analysis based on guidelines from the InterLymph Consortium^17^: MM, all LN, Hodgkin lymphoma (HL) and non‐Hodgkin lymphoma (NHL, excluding plasma cell neoplasms^18^); the latter included T‐cell NHL (T‐NHL) and B‐cell NHL (B‐NHL). We also analysed selected common mature B‐cell LN subtypes separately, including diffuse large B‐cell lymphoma (DLBCL), follicular lymphoma (FL) and CLL/SLL (Table S2 for disease classifications).

#### Blood collection and laboratory methods

Serum concentrations of total IGF‐I were measured using chemiluminescence immunoassays (DiaSorin Liaison XL, analytic range 1.3–195 nmol/L). Measurements were conducted at a purpose‐built laboratory for UK Biobank in Stockport, England, and the average within‐laboratory coefficients of variation (ratio of the SD to the mean) were 6.03%, 5.29% and 6.18% for low, medium and high concentrations respectively. Further information can be found in Supplementary Methodology S1.

#### Statistical analysis

Cox proportional hazard models with different levels of covariate adjustment based on a priori assumptions^19^, ^20^, ^21^, ^22^, ^23^ were used, both of which were stratified by age (years). For information on selected variables and categorisation, see Supplementary Methodology S1. Model 1 adjusted for sex, education, UK region and Townsend deprivation index. Model 2 further adjusted for ethnicity, height, BMI, smoking status, alcohol consumption, vigorous physical activity, diabetes history and fasting time. We analysed IGF‐I levels as categorical (sex‐specific quartiles) and continuous variables (per sex‐specific 1‐standard deviation [SD] increase; 1‐SD = 5.7 nmol/L; 5.8 nmol/L and 5.5 nmol/L for all participants, among females and among males respectively). Model 2 was additionally corrected for a regression dilution ratio for IGF‐I (0.77) obtained from the subsample of 17 697 participants with repeated IGF‐I measurement;^24^, ^25^ specifically, to obtain corrected HRs, the log HRs and their standard errors were divided by 0.77 and then exponentiated.^26^ We also obtained results stratified by time from recruitment to the end of follow‐up (<5 years, 5–9 years, 10 or more years) and assessed multiplicative interactions between the continuous concentration of IGF‐I and the following variables by including an interaction term in model 2: sex, BMI (<25, ≥25 kg/m^2^), smoking status (never, ever), alcohol intake (never, ever), ethnicity (white, other), height (≤, >sex‐specific median) for the end‐points (MM, LN subtypes alone and categorised as NHL and B‐NHL). As a secondary analysis,^13^, ^14^ we examined the interaction between the continuous concentration (per 1‐SD increase) of IGF‐I and BMI in relation to the risk of MM and CLL/SLL, and we investigated a potential three‐way interaction of IGF‐I, sex and BMI in relation to risk of those end‐points. As sensitivity analyses, we additionally adjusted model 2 for markers of inflammatory, sex hormone and glycaemic pathways that are known to inter‐relate/cross‐talk with the IGF system, namely, C‐reactive protein (CRP), testosterone, sex hormone‐binding globulin (SHBG) and glycosylated haemoglobin (HbA1c)^27^ (model 3), excluding the first 2 years of follow‐up and examined the main findings (e.g. model 2 with regression dilution correction) excluding those in the highest and lowest 1% of the IGF‐I distribution (*n* = 4442) (Supplementary Methodology S1).

### Mendelian randomisation study

#### Genetic determinants of IGF‐I and IGFBP‐3 circulating levels

We used previously described genetic instruments^28^ (Supplementary Methodology S1). For a summary of the genetic instruments and of the corresponding single nucleotide polymorphism (SNP)‐specific associations with IGF‐I and IGFBP‐3 levels and with the different LN subtypes, see Table S3.

#### Data on LN subtypes

We obtained estimates of the association of IGF‐I and IGFBP‐3 genetic markers with LN subtypes from summary data from GWAS meta‐analyses conducted within the InterLymph consortium, which included 2434 MM and their 2567 controls of European ancestry as well as 3100 CLL/SLL, 2847 FL and 3857 DLBCL and their 8542 controls.^29^, ^30^, ^31^, ^32^ Table S4 lists the InterLymph studies included in this analysis.

#### Statistical analysis

Prior to the analysis, the alleles were harmonised by aligning the effect allele for the exposure (IGF‐I and IGFBP‐3) and the outcomes (MM and LN subtypes), thus ensuring that the effect alleles were the same across both datasets. We used the three most common models to estimate separately the potential effect of genetically predicted levels for the IGF‐I‐ and IGFBP‐3‐related instruments: Inverse variance‐weighted (IVW) models, weighted median models and MR‐Egger method.^33^ We employed the latter to evaluate causal relationships between genetic instruments and outcomes, allowing for the detection of pleiotropy and providing robust causal effect estimates.^34^ GWAS summary statistics from the IEU OpenGWAS and GWAS catalogue for BMI‐associated SNPs were used to test potential violation of the independence assumption. The MR‐PRESSO (Mendelian Randomisation Pleiotropy RESidual Sum and Outlier)^35^ distortion test was employed to assess the presence of horizontal pleiotropy (see Supplementary Methodology S1).

## RESULTS

### Observational study: The UK biobank

The final analytical cohort included 444 187 men and women. During a mean (SD) of 10.5 (1.7) years of follow‐up, 121 incident HL and 2430 incident cases of other LN types were identified; the latter included 732 MM, 667 CLL/SLL, 680 DLBCL, 418 FL and 163 T‐NHL. At enrolment, cohort participants had a mean age of 56 years and a higher proportion of females (*n* = 239 048; 54%) than males (Table 1). Compared to participants with circulating levels of IGF‐I within the lower quartiles, those in the highest quartile (Q4) were younger, had lower BMI, were taller, more likely to be non‐smokers, less likely to have self‐reported diabetes and less likely to be in the most deprived (third tertile) category of the Townsend index (Table 1). Overall, the mean IGF‐I level was 21.4 (SD: 5.7 nmol/L), with a range from 1.4 nmol/L to 126.8 nmol/L. Among LN subtypes, the highest IGF‐I values were observed among participants who subsequently developed MM and CLL/SLL.

**TABLE 1:** Select UK Biobank participant characteristics at recruitment overall and by quartile of circulating IGF‐I level.

	Overall	IGF‐I quartile			
		Q1	Q2	Q3	Q4
Number	444 187	111 058	111 067	111 036	111 026
Female: mean (SD) Min to max, nmol/L	21.0 (5.8)	14.1 (2.2)	18.9 (1.1)	22.6 (1.1)	28.5 (4.0)
	1.4 to 125.1	1.45 to 18.3	17.0 to 21.7	20.8 to 25.2	24.5 to 126.8
Male: mean (SD) Min to max, nmol/L	21.9 (5.5)	15.2 (2.4)	20.1 (1.0)	23.4 (1.0)	28.9 (3.9)
	1.9 to 126.8	1.9 to 18.3	18.3 to 21.8	21.8 to 25.2	25.2 to 126.8
Person‐years, mean (SD) years^a^	10.5 (1.7)	10.4 (1.8)	10.5 (1.6)	10.6 (1.6)	10.6 (1.6)
Female, *n* (%)	239 048 (53.8)	59 773 (53.8)	59 764 (53.8)	59 764 (53.8)	59 747 (53.8)
Age, mean (SD) years	56.4 (8.1)	59.0 (7.2)	57.2 (7.7)	55.7 (8.1)	53.6 (8.4)
Body mass index, mean (SD) kg/m^2^	27.4 (4.8)	28.5 (5.6)	27.4 (4.7)	27.0 (4.4)	26.7 (4.1)
Height, mean (SD) cm	168.6 (9.3)	167.5 (9.2)	168.3 (9.2)	168.9 (9.3)	169.5 (9.3)
Townsend deprivation index 3rd tertile, most deprived *n* (%)	147 867 (33.3)	40 759 (36.7)	36 693 (33.0)	35 244 (31.7)	35 171 (31.7)
Vigorous physical activity for >10 min, mean (SD)days/week	1.9 (2.0)	1.7 (2.0)	1.9 (2.0)	1.9 (2.0)	1.9 (1.9)
Never smoker, *n* (%)	242 695 (54.6)	55 537 (50.0)	59 510 (53.6)	62 294 (56.1)	65 354 (58.9)
Never alcohol intake, *n* (%)	19 508 (4.4)	6149 (5.5)	4674 (4.2)	4403 (4.0)	4282 (3.9)
Fasting (>4 h since last food intake), *n* (%)	103 794 (23.4)	28 153 (25.3)	26 045 (23.4)	25 290 (22.8)	24 306 (21.9)
Self‐reported ethnicity, white *n* (%)	418 123 (94.1)	104 367 (94.0)	104 900 (94.4)	104 788 (94.4)	104 068 (93.7)
Self‐reported diabetes history, *n* (%)	22 949 (5.2)	8892 (8.0)	5112 (4.6)	4526 (4.1)	4419 (4.0)
Sex hormone‐binding globulin, mean (SD) nmol/L	51.5 (27.7)	56.8 (32.1)	52.2 (27.3)	50.1 (25.7)	46.8 (24.1)
Testosterone, mean (SD) nmol/L	6.6 (6.1)	6.8 (6.2)	6.7 (6.1)	6.6 (6.0)	6.5 (5.9)
Glycated haemoglobin, mean (SD) mmol/mol	36.1 (6.8)	37.3 (8.3)	36.1 (6.5)	35.7 (6.0)	35.3 (5.9)
C‐reactive protein, mean (SD) mg/L	2.6 (4.3)	3.5 (5.2)	2.6 (4.1)	2.2 (3.7)	1.9 (3.8)
LN, *n* (%)	3321 (0.7)	971 (0.9)	863 (0.8)	790 (0.7)	697 (0.6)
MM, *n* (%)	732 (0.2)	198 (0.2)	172 (0.2)	181 (0.2)	181 (0.2)
HL, *n* (%)	121 (0.0)	41 (0.0)	33 (0.0)	28 (0.0)	19 (0.0)
NHL, *n* (%)	2430 (0.5)	726 (0.7)	644 (0.6)	567 (0.5)	493 (0.4)
B‐NHL, *n* (%)	2133 (0.5)	647 (0.6)	557 (0.5)	498 (0.4)	431 (0.4)
CLL/SLL, *n* (%)	667 (0.2)	217 (0.2)	163 (0.1)	159 (0.1)	128 (0.1)
DLBCL, *n* (%)	680 (0.2)	194 (0.2)	193 (0.2)	150 (0.1)	143 (0.1)
FL, *n* (%)	418 (0.1)	129 (0.1)	113 (0.1)	103 (0.1)	73 (0.1)
T‐NHL, *n* (%)	163 (0.0)	45 (0.0)	48 (0.0)	39 (0.0)	31 (0.0)
Mean IGF‐I (SD), nmol/L					
MM	21.3 (5.7)	14.5 (2.5)	19.5 (1.1)	23.2 (1.0)	28.5 (3.6)
HL	20.3 (5.6)	14.5 (2.4)	19.8 (1.3)	23.0 (1.1)	29.3 (4.0)
CLL/SLL	20.9 (7.0)	14.7 (2.3)	19.5 (1.2)	23.2 (1.1)	30.3 (9.2)
DLBCL	20.8 (5.7)	14.4 (2.4)	19.6 (1.2)	23.0 (1.1)	28.9 (3.9)
FL	20.5 (5.6)	14.5 (2.6)	19.5 (1.2)	23.0 (1.2)	29.0 (4.4)
T‐NHL	20.8 (5.4)	14.7 (2.8)	19.6 (1.1)	23.2 (1.3)	28.7 (3.2)

*Note*: LN classification in Table S2.

Abbreviations: CLL/SLL, chronic lymphocytic leukaemia/small lymphocytic leukaemia; DLBCL, diffuse large B‐cell lymphoma; FL, follicular lymphoma; HL, Hodgkin lymphoma; IGF, insulin‐like growth factor; LN, lymphoid neoplasm; MM, multiple myeloma; NHL, non‐Hodgkin lymphoma; Q, sex‐specific quartile; SD, standard deviation.

^a^ Person‐years were contributed from the age at entry into the UK Biobank study until the earliest among age at registration of a lymphoid neoplasm of interest, the age at exit from participation in the study or death or last complete follow‐up.

We detected a statistically significant interaction between sex and circulating IGF‐I levels with the main aggregated endpoints (LN, NHL and B‐NHL; Table S5) and decided to present results for the whole cohort and stratified by sex. In multivariate models, higher levels of IGF‐I were associated with increased MM risk (corrected HR_1‐SD_ = 1.11; 95% confidence interval [CI]: 1.01–1.22; *p*‐value = 0.03), whereas IGF‐I levels were not associated with risk of LN, HL, NHL, B‐NHL or LN subtypes (Table 2; for quartile‐specific findings, see Table S6). In the sex‐specific models, we observed an increased risk of CLL/SLL per 1‐SD increase in IGF‐I levels in males but a suggested inverse association in females (males, corrected HR_1‐SD_: 1.13, 95% CI: 1.00–1.28; females, HR_1‐SD_: 0.87, 95% CI: 0.73–1.04; *p*‐value for interaction = 0.01). A three‐way interaction for the association between CLL/SLL and IGF‐I was detected between sex and BMI variables (*p*‐value for interaction = 0.002 and 0.004 for two‐ and three‐level BMI variable, respectively; Table S5 and Table 3). Specifically, we noted the strongest associations of circulating IGF‐I levels with CLL/SLL risk in males with higher BMI (corrected HR_1‐SD_ = 0.79, 1.12 and 1.36 among normal BMI, overweight and obese males respectively). No interaction by sex or BMI was detected for MM. However, the association between IGF‐I levels and MM was stronger among females than males (corrected HR_1‐SD_ for MM: 1.16, 95% CI: 1.00–1.34 in females and 1.06, 95% CI: 0.94–1.20 in males; P‐value for interaction = 0.54; Table 3).

**TABLE 2:** Hazard ratios and 95% confidence intervals (CI) for risk of lymphoid neoplasms per 1‐SD increase in circulating IGF‐I, overall and by sex in the UK Biobank cohort.

	Overall		Female		Male		Test for interaction^c^
	HR^a^ (95% CI)	*p*‐value	HR^a^ (95% CI)	*p*‐value	HR^a^ (95% CI)	*p*‐value	
LN, *n*	3321		1442		1879		
Model^a^	1.01 (0.98–1.05)	0.45	0.97 (0.92–1.03)	0.31	1.04 (1.00–1.09)	0.07	
Model^b^	1.01 (0.98–1.05)	0.43	0.97 (0.92–1.03)	0.34	1.04 (0.99–1.09)	0.10	0.08
Corrected^b^	1.02 (0.97–1.07)		0.97 (0.90–1.04)		1.05 (0.99–1.12)		
MM, *n*	732		323		409		
Model^a^	**1.08 (1.01–1.16)**	**0.03**	1.11 (1.00–1.25)	0.06	1.06 (0.96–1.17)	0.26	
Model^b^	**1.08 (1.01–1.17)**	**0.03**	**1.12 (1.00–1.25)**	**0.05**	1.05 (0.95–1.15)	0.36	0.54
Corrected^b^	**1.11 (1.01–1.22)**		**1.16 (1.00–1.34)**		1.06 (0.93–1.21)		
HL, *n*	121		53		68		
Model^a^	0.83 (0.69–1.01)	0.06	0.80 (0.59–1.08)	0.14	0.85 (0.66–1.10)	0.23	
Model^b^	0.86 (0.71–1.05)	0.14	0.79 (0.58–1.06)	0.12	0.91 (0.71–1.16)	0.44	0.92
Corrected^b^	0.83 (0.64–1.06)		0.73 (0.49–1.08)		0.88 (0.64–1.21)		
NHL, *n*	2430		1052		1378		
Model^a^	1.00 (0.96–1.05)	0.89	0.94 (0.88–1.00)	0.06	1.05 (0.99–1.11)	0.08	
Model^b^	1.00 (0.96–1.04)	0.97	0.94 (0.88–1.00)	0.06	1.05 (0.99–1.11)	0.11	**0.01**
Corrected^b^	1.00 (0.95–1.06)		0.92 (0.84–1.00)		1.06 (0.99–1.13)		
B‐NHL, n	2133		922		1211		
Model^a^	1.01 (0.96–1.05)	0.72	0.94 (0.87–1.01)	0.08	**1.06 (1.00–1.12)**	**0.05**	
Model^b^	1.01 (0.96–1.05)	0.79	0.94 (0.88–1.01)	0.09	1.05 (0.99–1.11)	0.08	**0.01**
Corrected^b^	1.01 (0.95–1.07)		0.92 (0.84–1.01)		1.07 (0.99–1.15)		
*CLL/SLL, n*	667		261		406		
Model^a^	1.02 (0.95–1.11)	0.55	0.90 (0.79–1.03)	0.12	**1.10 (1.00–1.21)**	**0.05**	
Model^b^	1.02 (0.94–1.11)	0.59	0.90 (0.79–1.03)	0.12	**1.10 (1.00–1.21)**	**0.05**	**0.01**
Corrected^b^	1.03 (0.93–1.14)		0.87 (0.73–1.04)		**1.13 (1.00–1.28)**		
*DLBCL, n*	680		311		369		
Model^a^	1.02 (0.94–1.10)	0.69	0.99 (0.88–1.11)	0.85	1.04 (0.94–1.15)	0.47	
Model^b^	1.02 (0.94–1.10)	0.63	1.00 (0.89–1.13)	0.99	1.03 (0.93–1.15)	0.52	0.56
Corrected^b^	1.03 (0.93–1.14)		1.00 (0.85–1.17)		1.04 (0.91–1.20)		
*FL, n*	418		223		195		
Model^a^	0.92 (0.83–1.02)	0.11	0.88 (0.76–1.02)	0.08	0.96 (0.83–1.12)	0.62	
Model^b^	0.92 (0.83–1.02)	0.10	0.88 (0.76–1.02)	0.09	0.96 (0.82–1.11)	0.56	0.33
Corrected^b^	0.89 (0.78–1.02)		0.85 (0.70–1.02)		0.94 (0.78–1.15)		
**T‐NHL, n**	163		68		95		
Model^a^	0.96 (0.82–1.13)	0.63	0.85 (0.65–1.10)	0.21	1.04 (0.84–1.28)	0.71	
Model^b^	0.96 (0.81–1.13)	0.61	0.83 (0.64–1.08)	0.17	1.04 (0.85–1.28)	0.68	0.27
Corrected^b^	0.95 (0.76–1.17)		0.78 (0.55–1.11)		1.06 (0.81–1.38)		

*Note*: LN classification in Table S2. Bold indicates a statistically significant association at the 5% level of significance.

Abbreviations: CLL/SLL, chronic lymphocytic leukaemia/small lymphocytic leukaemia; DLBCL, diffuse large B‐cell lymphoma; FL, follicular lymphoma; HL, Hodgkin lymphoma; IGF, insulin‐like growth factor; LN, lymphoid neoplasms; MM, multiple myeloma; NHL, non‐Hodgkin lymphoma; SD, standard deviation.

^a^ Model 1: adjusted for sex, qualification, UK parts (England/Wales, Scotland), Townsend index (quintiles). Model 2: model 1 + BMI (<25, 25–29, ≥ 30 kg/m^2^), ethnicity (white, black or black British, other), vigorous physical activity, height (sex‐specific tertiles), alcohol (never, previous, current), smoking (never, past, current), diabetes (yes/no) and fasting status (tertiles).

^b^ HRs per 1‐SD increment from model 2 were additionally corrected for a regression dilution ratio (0.77) obtained from the subsample of 17 697 participants with repeat IGF‐I measurements.

^c^ Multiplicative interactions between the continuous concentration (per 1‐SD increase) of IGF‐I and sex were assessed by including an interaction term in model 2.

**TABLE 3:** Hazard ratios (HR) and 95% confidence intervals (CI) for risk of chronic lymphocytic leukaemia (CLL/SLL) per 1‐SD increase in circulating IGF‐I, overall and jointly by sex and body mass index at enrolment in the UK Biobank cohort.

Body mass index (kg/m^2^)	Overall		Female		Male	
	*n*	HR^a^ (95% CI)	*n*	HR^a^ (95% CI)	*n*	HR^a^ (95% CI)
<25	189	0.86 (0.69–1.06)	103	0.94 (0.71–1.25)	86	0.79 (0.58–1.07)
25–29	310	1.06 (0.91–1.23)	92	0.91 (0.67–1.22)	218	1.12 (0.94–1.33)
≥30	167	1.15 (0.96–1.38)	66	0.76 (0.53–1.08)	101	**1.36 (1.14–1.61)**

*Note*: Two‐way interaction P‐value (sex and three‐level body mass index) = 0.004. Bold indicates a statistically significant association at the 5% level of significance.

Abbreviation: IGF, insulin‐like growth factor.

^a^ HRs per 1‐SD increment were corrected for a regression dilution ratio (0.77) obtained from the subsample of 17 697 participants with repeat IGF‐I measurements.

The sensitivity analyses revealed no meaningful differences (Tables S6–S8). When examined separately by follow‐up period (Table 4), the strongest associations were observed for risk of MM within a shorter time period after blood draw (corrected HR_1‐SD_: 1.22, 1.03 and 1.02 within <5 years, 5–9 years and 10 or more years respectively).

**TABLE 4:** Hazard ratios (HR) and 95% confidence intervals (CI) for risk of lymphoid neoplasms per 1‐SD increase in circulating IGF‐I by time since recruitment in the UK Biobank cohort.

	Time from recruitment to LN								
	<5 yrs			5–9 yrs			10+ yrs		
	*N*	HR^a^ (95% CI)	*p*‐value	*N*	HR^a^ (95% CI)	*p*‐value	*N*	HR^a^ (95% CI)	*p*‐value
LN	1239	1.03 (0.96–1.10)	0.48	1731	0.99 (0.93–1.06)	0.80	351	1.12 (0.98–1.29)	0.11
MM	228	**1.22 (1.04–1.44)**	**0.01**	421	1.03 (0.90–1.17)	0.68	83	1.02 (0.76–1.37)	0.90
HL	51	0.92 (0.64–1.33)	0.65	59	0.81 (0.57–1.15)	0.23	11	0.90 (0.39–2.09)	0.80
NHL	952	1.00 (0.92–1.08)	0.99	1233	0.99 (0.91–1.06)	0.74	245	1.15 (0.98–1.36)	0.09
B‐NHL	793	0.98 (0.89–1.07)	0.61	1116	1.01 (0.93–1.09)	0.78	224	1.18 (1.00–1.39)	0.05
CLL/SLL	260	1.03 (0.88–1.20)	0.71	343	1.01 (0.88–1.17)	0.86	64	1.27 (0.94–1.71)	0.12
DLBCL	252	1.01 (0.86–1.18)	0.91	345	0.99 (0.86–1.15)	0.94	83	1.24 (0.96–1.60)	0.09
FL	168	0.95 (0.78–1.16)	0.62	217	0.85 (0.70–1.03)	0.09	33	0.89 (0.55–1.44)	0.63
T‐NHL	76	1.12 (0.83–1.50)	0.46	74	0.81 (0.58–1.12)	0.19	13	0.85 (0.39–1.87)	0.68

*Note*: Sex‐specific quartiles. Model2: adjusted for sex, qualification, UK parts (England/Wales, Scotland), Townsend index (quintiles), BMI (<25, 25–29, > = 30 kg/m^2^), ethnicity (white, black or black British, other), vigorous physical activity, height (sex‐specific tertiles), alcohol (never, previous, current), smoking (never, past, current), diabetes (yes/no) and fasting status (tertiles). Bold indicates a statistically significant association at the 5% level of significance.

Abbreviations: CLL/SLL, chronic lymphocytic leukaemia/small lymphocytic leukaemia; DLBCL, diffuse large B‐cell lymphoma; FL, follicular lymphoma; HL, Hodgkin lymphoma; IGF, insulin‐like growth factor; LN, lymphoid neoplasms; MM, multiple myeloma; NHL, non‐Hodgkin lymphoma; SD, standard deviation; yrs, years.

^a^ HRs per 1‐SD increment were corrected for a regression dilution ratio (0.77) obtained from the subsample of 17 697 participants with repeat IGF‐I measurements.

### Mendelian randomisation

A 1‐SD increment (around 5 nmol/L) in genetically inferred circulating IGF‐I levels was associated with a 27% increase of MM risk (OR = 1.27, 95% CI: 1.05–1.54) (Table 5). For the other LN subtypes, no association was observed for genetically inferred IGF‐I levels, and we found no association of genetically inferred IGFBP‐3 levels with any LN end‐point. The estimates obtained from the weighted‐median approach and MR‐Egger were consistent with those obtained from IVW for all subtypes and for both IGF‐I and IGFBP‐3. As a sensitivity analysis, SNPs associated with BMI were excluded. The causal estimates remained essentially unchanged (*data not shown*). The MR‐PRESSO test identified potential horizontal pleiotropy for the associations of genetically inferred IGF‐I levels with FL and CLL/SLL (global test p‐value 0.001 and <1 × 10^−4^ for FL and CLL/SLL respectively). Removing outliers did not materially change the estimates, suggesting that outlying SNPs were not influencing the original results. No evidence of horizontal pleiotropy was found for MM or DLBCL, supporting the robustness of the estimates (Supplementary Methodology S1, Figure S9).

**TABLE 5:** Mendelian randomisation (MR) analysis estimates for associations between genetically inferred circulating insulin‐like growth factor‐I (IGF‐I) and insulin‐like growth factor‐binding protein‐3 (IGFBP‐3) levels and risk of lymphoid malignancies in the InterLymph Consortium GWAS study sample.

	Inverse‐variance‐weighted	Weighted median	MR‐Egger
	OR (95% CI) per 1‐SD increment	OR (95% CI) per 1‐SD increment	OR (95% CI) per 1‐SD increment
IGF‐I (*n* = 413 SNPs)			
MM	**1.27 (1.05–1.54)**	1.34 (0.98–1.84)	1.26 (0.80–1.98)
CLL/SLL	0.95 (0.80–1.12)	0.96 (0.75–1.22)	1.15 (0.79–1.67)
DLBCL	1.09 (0.94–1.26)	1.12 (0.88–1.42)	1.21 (0.87–1.67)
FL	1.08 (0.91–1.28)	1.04 (0.80–1.35)	0.90 (0.61–1.32)
IGFBP‐3 (*n* = 4 SNPs)			
MM	1.02 (0.81–1.29)	1.04 (0.81–1.34)	1.12 (0.65–1.93)
CLL/SLL	0.96 (0.80–1.14)	0.98 (0.80–1.19)	1.07 (0.70–1.64)
DLBCL	1.00 (0.85–1.19)	1.01 (0.84–1.22)	0.98 (0.65–1.46)
FL	0.86 (0.71–1.04)	0.84 (0.68–1.03)	0.77 (0.49–1.20)

Abbreviations: CI, confidence interval; CLL/SLL, chronic lymphocytic leukaemia/small lymphocytic leukaemia; DLBCL, diffuse large B‐cell lymphoma; FL, follicular lymphoma; MM, multiple myeloma; OR, odds ratio; SD, standard deviation.

## DISCUSSION

In the observational analysis, IGF‐I levels were positively associated with MM risk overall and, in a secondary analysis, with CLL/SLL risk among males (especially those with higher BMI) but not among females. The MR analysis yielded a positive association between genetically inferred circulating IGF‐I levels and MM risk, thus supporting the involvement of the IGF‐I pathway in MM development. In contrast, we did not observe an association of genetically inferred IFGBP‐3 levels with any LN subtype.

Our results are consistent with previous data from the UK Biobank with only 367 cases^7^ and a pooled study nested in six cohorts comprising the MMCC^10^ that included 493 MM and 978 controls but were inconsistent with a nested case–control study within the EPIC cohort, which included 237 MM and matched controls (Table S10).^11^ Supporting our results, Birmann et al. did not report interaction by sex or BMI in the association between IGF‐I or related biomarker levels and MM.^10^ Although these studies—including ours—had large sample sizes, they were limited in their ability to stratify by time (mean time from blood draw to diagnosis: 9 [SD: 2] years and 6.6 [range 0.3–18.1] years for the EPIC and the MMCC studies respectively), and the large 95% CIs precluded definitive conclusions. Of note, however, in the present analysis, we also observed the strongest association with MM diagnosis <5 years after blood draw, which might reflect disease progression from monoclonal gammopathy of undetermined significance (MGUS) to MM.

IGF is a complex system,^9^, ^36^ and by examining total IGF‐I, we did not disentangle free (bioavailable) and bound IGF‐I. Indeed, the bioavailability of IGF‐I is strictly controlled by IGFBPs and in particular by the abundant IGFBP‐3.^37^ Free IGF‐I can bind to IGF‐1 receptor (IGF‐1R), which might produce cell proliferation as well as inhibition of apoptosis.^38^ MM cells have autocrine IGF‐I production that have been associated with cell survival and disease progression^39^, ^40^, ^41^, ^42^ and MM cells display aberrant IGF‐1R expression.^43^ Hence, serum total circulating IGF‐I levels might not be the best biomarker to study the IGF‐I system. However, there is currently no genetic instrument as a proxy for free IGF‐I exposure, and our findings serve as a preliminary step and underscore the need for future MR studies incorporating free IGF‐I levels. The MMCC study reported a non‐significant positive association between IGFBP‐3 levels and MM Hazard ratio‐HR‐per one standard deviation‐SD‐increase (HR_1SD_: 1.1; 95% CI: 1.0–1.2; *p*‐value: 0.08 and HR_Q4 vs. Q1_: 1.4; 95% CI: 1.0–1.9). However, the molar ratio of IGF‐I to IGFBP‐3 (an empirical indicator of IGF‐I bioavailability) was not associated with MM risk. Additionally, the MMCC study noted a positive association between pre‐diagnosis IGFBP‐1 levels and MM risk,^10^ with the strongest association within 3 years after blood draw. Further examination of additional IGF pathway signalling molecules is warranted.

Consistent with our results, the case–control study nested within the EPIC cohort^11^ detected no overall association between CLL/SLL and pre‐diagnosis IGF‐I concentrations; conventional (retrospective) case–control studies have reported contradictory results.^14^, ^44^, ^45^, ^46^ The EPIC study detected borderline statistically significant separate interactions of IGF‐I levels and LN with sex and with BMI but did not present results stratified jointly by sex and BMI; in the EPIC study, circulating IGF‐I levels were inversely associated with LN risk (all subtypes grouped) in women and in individuals with normal BMI.^11^ In the EpiLymph and the Spanish multi‐case–control (MCC‐Spain) studies, we examined circulating IGF‐I and IGFBP‐3 levels among 209 untreated CLL/SLL patients and their age‐ and sex‐matched controls. IGF‐I levels were positively associated with CLL/SLL risk among obese patients, but interactions of BMI with sex, a matching variable, were not assessed.^14^ In the current examination of those interactions, we found a stronger positive association of circulating IGF‐I with CLL/SLL among overweight/obese men. Of interest, the inverse association observed between MM risk and pre‐diagnosis plasma adiponectin, an adipokine, varied by BMI in the MMCC study.^47^ These findings warrant further investigation of these biomarkers in studies that include data on sex and BMI, in order to clarify potential stratum‐specific associations.

Observational studies are prone to residual confounding and reverse causation. MR uses genetic variants as instrumental variables to assess potential effects, mimicking randomisation and reducing residual confounding. The present MR analysis supported the observational data for MM, the plausibility of which is further supported by extensive laboratory studies reporting a role for the IGF‐I system in the growth, survival, apoptosis and migration of MM cells.^9^, ^36^, ^40^, ^48^, ^49^, ^50^ Based on the laboratory evidence, several clinical trials have also been designed in recent decades to test putative therapeutic molecules that target the IGF‐I receptor.^51^, ^52^, ^53^ Overall results from our MR study of CLL/SLL did not support a role of IGF‐I, but heterogeneity of the estimates by sex and BMI could not be evaluated by MR since sex‐specific data were not available. To address the key MR assumption of no horizontal pleiotropy, we conducted sensitivity analyses, which supported the robustness of our estimates for IGF‐I and MM.

This is the largest and most comprehensive study to date, exploring the role of IGF‐I in LN subtype development using two complementary designs. However, despite the large sample size, stratified results were based on small numbers of cases. Furthermore, we studied only total IGF‐I levels in the observational study, and the IGF‐I instrument variable in the MR analyses was likewise related to total rather than free IGF‐I. Those limitations do not support inferences regarding bioavailable IGF‐I per se, and we did not observe associations for the IGFBP‐3 instrument variable. Our main hypothesis was on the association between IGF‐I levels and MM. We included other LN subtypes as comparators, allowing us to determine whether any observed associations were unique to MM or indicative of broader LN patterns. While our results would not remain statistically significant after adjusting for multiple testing, our hypothesis‐driven framework ensured that the observed findings remain scientifically relevant and worthy of further investigation. Another important point is the small sample sizes in stratified groups that result in wider confidence intervals and less precise estimates. This highlights the need to interpret findings within these subgroups with caution. If feasible, increasing the sample size in future studies could help improve the stability and reliability of these estimates. Therefore, we recommend caution in interpreting our findings until additional large, prospective studies with information on relevant covariates can confirm them. Finally, the prevalence of MGUS among the different studies was not known and might have affected the results.

In conclusion, this work is consistent with a direct relationship between MM and IGF‐I levels. However, given the modest effect sizes observed, the MR findings should be contextualised within the inherent limitations of genetic instrumental variable analyses and limited statistical power. In turn, further studies are needed to better understand the relevance of other components of the IGF‐I signalling pathway (e.g. IGF‐1R) and of bioavailable IGF‐I, to confirm the aetiologically relevant timing of IGF‐I signalling for these end‐points and to further elucidate the apparent difference in associations that we observed between circulating IGF‐I levels and CLL/SLL by sex and BMI. Future work should also include participants with precursor stages of CLL/SLL and MM (monoclonal B‐cell lymphocytosis and MGUS, respectively) to better understand the IGF‐I associations.

## AUTHOR CONTRIBUTIONS

DC designed the research study. DC and YB performed the research. DC, YB and SH analysed the data. DC, YB, LA, SH and BMB involved in funding acquisition. DC, YB, SH and BMB wrote the paper. All authors involved in data acquisition, paper revision and approval of the submitted and final versions.

## FUNDING INFORMATION

This work was partially supported by Instituto de Salud Carlos III—ISCIII (Spanish Government) co‐funded by FEDER funds/European Regional Development Fund (ERDF)—a way to build Europe (CIBERESP CB06/02/0073, PI20/00288, FI21/00090, PI20/01845 and PI25/00156). Additional support came from the Agency for Management of Universities and Research Grants (AGAUR; 2021SGR01354, 2014SGR756) and the Consejería de Salud y Familia de la Junta de Andalucía (PY20/01282) and FEDER. We thank the Fundacio Institució dels Centres de Recerca de Catalunya (CERCA) Program/Generalitat de Catalunya for institutional support to IDIBELL. The InterLymph data coordinating centre (DCC) was supported by the NCI/U01CA257679. Information on funding for individual studies can be found in Table S4.

## CONFLICT OF INTEREST STATEMENT

The authors declare no conflicts of interest.

## ETHICAL APPROVAL STATEMENT

The UK biobank study received ethical approval from relevant committees and is overseen by an independent Ethics and Governance Council (https://www.ukbiobank.ac.uk/learn‐more‐about‐uk‐biobank/about‐us/ethics). All studies from the InterLymph consortium were approved by their respective ethical committees.

## Supporting information


Data S1.

## Data Availability

Genotype data from the NCI NHL GWAS are available on dbGaP (phs000801.v2.p1) for research purposes in accordance with dbGaP data access policies. Other data in this manuscript are available for shared research purposes through the InterLymph Consortium upon approval in accordance with the InterLymph data sharing framework, institutional review boards and general data protection regulations (https://datacollect.iarc.fr/redcap/surveys/?s=73H8NHL8PN8XCJ4J). The UK Biobank data can be accessed applying at https://www.ukbiobank.ac.uk/enable‐your‐research/apply‐for‐access.
