# Supplementary material for: Circulating levels of insulin‐like growth factor I (IGF‐I) and risk of multiple myeloma: An observational and Mendelian randomisation study
Source: Br J Haematol. 2026 Mar 23;208(6):1942–53. doi: 10.1111/bjh.70444 (PMC13267475; doi:10.1111/bjh.70444)
Supplement: Supplementary file 1 — Data S1. [file BJH-208-1942-s001.pdf]

SUPPLEMENTARY MATERIAL

Title: Circulating levels of insulin-like growth factor I (IGF-I) and risk of multiple myeloma: an observational and Mendelian Randomisation study

Yolanda Benavente et al.

SUPPLEMENTARY METHODOLOGY S1………………..…………………………………. page 2

SUPPLEMENTARY TABLE S2. List of the lymphoid neoplasm classification……………………………………………………………………………….... page 6

SUPPLEMENTARY TABLE S3. Summary of the genetic instruments and the beta estimates for the association of each SNP with IGF-I and IGFBP-3 levels and with the different lymphoma subtypes……………………………………………..………………………………....... page 8

SUPPLEMENTARY TABLE S4. List of INTERLYMPH studies, their funding and special acknowledgements..………........ page15

SUPPLEMENTARY TABLE S5. Tests for interactions (likelihood ratio tests for multiplicative interactions) between serum IGF-I and sex, age, height, BMI, alcohol, smoking, and ethnicity in relation to risk of lymphoid neoplasm………………………………………………………….. page 20

SUPPLEMENTARY TABLE S6. Sensitivity analysis: Hazard ratios and 95% CI for the association of serum IGF-I with risk of lymphoid neoplasms, adjusted for C-reactive protein (CRP), testosterone, sex hormone binding globulin (SHBG), and glycosylated haemoglobin (HbA1c), overall and stratified by sex………………………...…………………………………………….…..…… page 21

SUPPLEMENTARY TABLE S7. Sensitivity analysis: Hazard ratios and 95% CI for the association of serum IGF-I with risk of multiple myeloma, excluding the first two years of follow-up, and with complete data analysis, overall and stratified by sex ....page 24

SUPPLEMENTARY TABLE S8. Sensitivity analysis: Hazard ratios (HR) and 95% confidence intervals (CI) for risk of chronic lymphocytic leukaemia (CLL/SLL) per 1-SD increase in circulating IGF-I, overall and jointly by sex and body mass index at enrolment, following exclusion of participants in the highest and lowest values, 1% of the distribution (n= 4,442) ……..….. page 25

SUPPLEMENTARY FIGURE S9. Sensitivity analysis: Scatter plot of SNP-IGF-I vs SNP-MM associations ………………………………………………………………………………. page 26

SUPPLEMENTARY TABLE S10. Comparison of published and current results from prospective studies on the relation between IGF-I levels and multiple myeloma……………….……..…page 27

References……………………………………………………………………………....…page 28

SUPPLEMENTARY METHODOLOGY S1

The UK Biobank study (https://www.ukbiobank.ac.uk/)**:**

Study characteristics:
The UK Biobank invited 9.2 million people to participate via postal invitations and telephone follow-up, with a 5.7% response rate. Participants were registered with the UK National Health Service (NHS) and lived within 25 miles of one of 22 assessment centres. At baseline, participants were asked to complete a self-administered touchscreen extensive questionnaire and underwent physical measurements. Further, blood samples were collected and centrifuged, and serum was separated and stored at 80ºC. The study received ethical approval from relevant committees and is overseen by an independent Ethics and Governance Council. Participants provided written informed consent and were followed using data linkage to national administrative data and electronic health care records.
Flow chart of UK Biobank participant inclusions and exclusions:

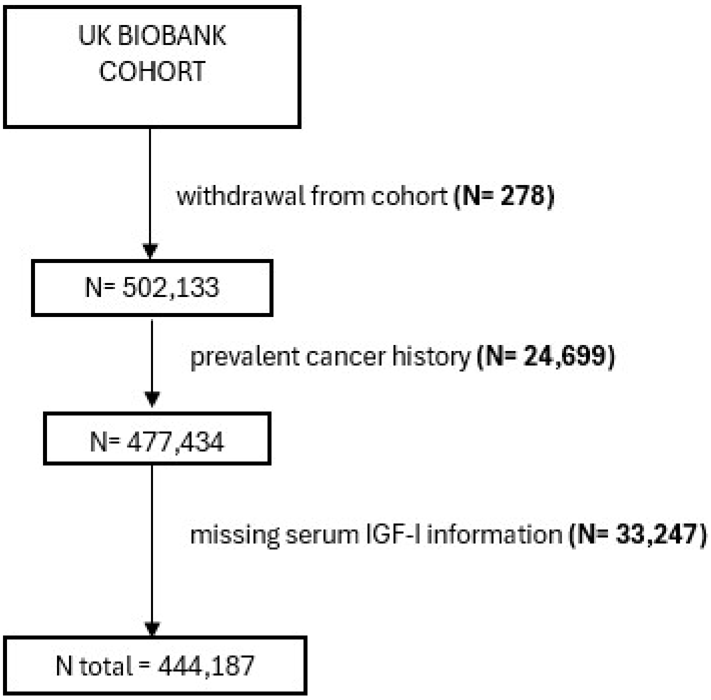

Selected covariates:
The covariate data derived from the physical measurements and touchscreen questionnaires administered at baseline and/or from linked electronic medical records. We calculated body mass index (BMI) as weight (kg) divided by height squared (m2) and categorized BMI into three analysis groups (<25kg/m2; 25-29kg/m2; >30kg/m2) and created sex-specific tertiles for height (cm). The Townsend deprivation index, an established British index which captures neighbourhood-level socioeconomic deprivation and for which lower values indicate less deprivation, was divided into tertiles. We also defined 3 categories for ethnicity (white, Black or Black British, and other), and educational attainment (college/university degree, A/O/ Certificate of Secondary Education [CSE] levels, and other level of attainment [national vocational qualification (NVQ) or higher national diploma (HND) or higher national certificate (HNC) or equivalent, other professional qualifications, e.g., nursing, teaching, none of the above]), as well as for alcohol consumption and tobacco smoking history (never, previous, and current). Geographical region was categorized into two groups (England/Wales and Scotland). Vigorous physical activity was based on the questions, “In

a typical week, how many days did you do 10 minutes or more of vigorous physical activity? These are activities that make you sweat and breathe hard such as fast cycling, aerobics, heavy lifting". We classified the responses into three groups (0, 1 to 3, and 4 to 7 days/week). We divided self-reported diabetes (any types based on the question: "has a doctor ever told you that you have diabetes?") into diabetic and non-diabetic groups. Finally, we defined fasting based on the question "time since last meal or drink (except plain water)" and categorized the responses into tertiles (<3, 4, and >5 hours). All adjustment variables were categorical, and missing data were classified in a separate category. The proportion of missing was as follows: educational attainment (1%), Townsend deprivation index (0.1%), BMI (0.4%), ethnicity (0.5%), vigorous physical activity (5.4%), height (0.3%), alcohol intake (0.3%), smoking status (0.5%), diabetes (0.4%) and fasting (<0.01%).

Blood collection and laboratory methods:

Non-fasting blood samples were collected from all participants at recruitment. A repeat assessment of 20,000 participants, facilitating correction for regression dilution bias,[1] was carried out between August 2012 and June 2013 at the UK Biobank Co-ordinating Centre, Stockport, UK. Participants who lived within a 35 km radius of the assessment centre were invited via email or letter, with an overall response rate of 21% (https://biobank.ndph.ox.ac.uk/~bbdatan/repeat_assessment_characteristics.pdf). Blood samples were centrifuged, and serum was stored at 80ºC. Serum concentrations of total IGF-I were measured using Chemiluminescence Immunoassays (DiaSorin Liaison XL, analytic range 1.3–195 nmol/L). Measurements were conducted at a purpose-built laboratory for UK Biobank in Stockport, England and the average within-laboratory coefficients of variation (ratio of the SD to the mean) were 6.03%, 5.29% and 6.18% for low, medium and high concentrations, respectively. With regard to additional serologic markers included in sensitivity analyses (see below), levels of testosterone and sex hormone binding globulin (SHBG) were determined by a chemiluminescent immunoassay (DXI 800, Beckman Coulter, London, UK), serum high sensitivity C-reactive protein (CRP) concentrations by an immuno-turbidimetric method (DXI 800), and glycated haemoglobin (HbA1c) concentrations by a high-performance liquid chromatography (HPLC) Variant II Turbo 2.0 system (Bio-Rad, Hercules, CA). Information on serum concentrations of IGF-I, CRP, HbA1c, testosterone and SHBG can be found in https://biobank.ndph.ox.ac.uk/showcase/showcase/docs/serum_biochemistry.pdf).[2] A total of 17,697 participants had IGF-I concentrations measured in blood samples collected at both the recruitment and repeat assessment visit.

Statistical methods:

Person-years were contributed from the age at entry into the UK Biobank study until the earliest among age at registration of multiple myeloma (MM) or a LN of interest, the age at exit from study participation or death, or last complete follow-up. For England and Wales, NHS England provided the cancer data, and the censoring date was 31 December 2020 (England) or 31 December 2016 (Wales). For Scotland, National Records of Scotland and the NHS Central Register provided the cancer data, and the censoring date was 30 November 2021. We report two-sided p-values with statistical significance set at $P < 0.05$.

Mendelian randomization study

Genetic determinants of IGF-I and IGFBP-3 circulating levels:

We used a 2-sample MR approach to support evidence consistent with a relationship between circulating IGF-I levels and the risk of MM and other main LN subtypes. This approach can provide evidence indicative of a possible direct association of a genetically inferred level of exposure with the outcome by combining the estimates of genetic markers associated with the exposure at a

genome-wide significance level (p-value<5x10-8) and the estimates of the associations of these variants with the outcome.

To ensure the robustness and reproducibility of our findings, we deliberately selected instrumental variables (IVs) previously identified and published by Murphy et al[3] for the same exposure. As both our study and the original publication derived from the UK Biobank and considering the rigorous selection criteria applied by our collaborators and co-authors, the chosen instruments provide a robust methodological foundation for our analysis. Briefly, the genetic instrument for IGF-I consists of 413 SNPs identified in the UKB GWAS, which includes 358,072 individuals. For IGFBP-3, the instrument comprises 4 SNPs identified in a meta-analysis of 13 studies (N=18,995). All participants in these studies were of European ancestry, and correlated variants (R2<0.01) among those reaching genome-wide significance were excluded.

Statistical Analysis:

Prior to the analysis, the alleles were harmonized by aligning the effect allele for the exposure (IGF-I and IGFBP-3) and the outcomes (LN subtypes), thus ensuring that the effect alleles were the same across both datasets.

Three assumptions are required for the validity of MR estimates: 1) the genetic instruments must be strongly associated with the outcome; 2) the genetic instruments must not be associated with any potential confounders of the relationship between the exposure and outcomes; and 3) there must be no horizontal pleiotropy, meaning the genetic instrument must not affect the outcome independently of the exposure. Firstly, we assessed the strength of the genetic instruments by referencing the F-statistic values reported by Murphy N et al. for each SNP associated with IGF-I and IGFBP-3, as we did not calculate these values independently. They reported that all individual F-statistic values surpassed the conventional threshold of 10, with a combined F-statistic of 89.9 for IGF-I and 308.4 for IGFBP-3, suggesting that weak instrument bias is unlikely. Additionally, the proportion of variance in IGF levels explained by the respective instruments was 9.4% for IGF-I and 6.1% for IGFBP-3.

As sensitivity analysis, we evaluated potential violations of the independence assumption by testing associations between IGF-I genetic instruments and the potential confounder, BMI, using GWAS summary statistics from the IEU OpenGWAS database. SNPs showing strong associations ($P < 1\times10^{-5}$) were excluded in sensitivity analyses. We employed the MR-Egger regression method to assess the robustness of our findings to potential violations of the instrumental variable assumptions, particularly pleiotropy. This approach allows for the detection and adjustment of directional pleiotropic effects, providing additional insight into the validity of our primary MR estimates.[5]The MR-PRESSO (Mendelian Randomisation Pleiotropy RESidual Sum and Outlier)[6] distortion test was employed to assess whether horizontal pleiotropy, resulting from any identified outlier SNPs, introduced bias into the effect estimates (P<0.05). Results were consistent across pleiotropy-robust MR methods

Statistical packages

All statistical analyses were carried out using R (version 4.2.1), RStudio (version 2022.07.1), and STATA-1 version 16.1 (StataCorp. 2021. Stata Statistical Software: Release 16.1. College Station, TX: StataCorp LLC). The analyses in this study were conducted using the *MendelianRandomization 0.10.0* package and *MRPRESSO 1.0* in RStudio 2023.12.0.

SUPPLEMENTARY TABLE S2. List of the lymphoid malignancy classification

ICD10	Histology	LN	HL	NHL	B-NHL	CLL/SLL	DLBCL	FL	T-NHL	MM
D472	9765	☑								
C837	9687	☑		☑	☑					
C911	9823	☑		☑	☑	☑				
C830	9670	☑		☑	☑	☑				
	9823	☑		☑	☑	☑				
C831	9673	☑		☑	☑					
C838	9673	☑		☑	☑					
C831	8000	☑		☑	☑					
C833	9680	☑		☑	☑		☑			
C834	9684	☑		☑	☑		☑			
C833	9590	☑		☑	☑		☑			
C820	9695	☑		☑	☑			☑		
C821	9691	☑		☑	☑			☑		
C829	9691	☑		☑	☑			☑		
C822	9698	☑		☑	☑			☑		
C823	9698	☑		☑	☑			☑		
C829	9698	☑		☑	☑			☑		
C829	9690	☑		☑	☑			☑		
C829	8000	☑		☑	☑			☑		
	9691	☑		☑	☑			☑		
	9690	☑		☑	☑			☑		
C914	9940	☑		☑	☑					
C914	9591	☑		☑	☑					
	9940	☑		☑	☑					
C830	9671	☑		☑	☑					
C838	9671	☑		☑	☑					
C880	9761	☑		☑	☑					
C857	9699	☑		☑	☑					
C859	9699	☑		☑	☑					
C884	9699	☑		☑	☑					
C830	9689	☑		☑	☑					
C833	9689	☑		☑	☑					
C900	9732	☑		☑	☑					☑
C900	8000	☑		☑	☑					☑
C509	9734	☑		☑	☑					
C902	9731	☑		☑	☑					
C903	9731	☑		☑	☑					
D479	9731	☑		☑	☑					
C910	9811	☑		☑	☑					
C910	9836	☑		☑	☑					
C851	9591	☑		☑	☑					
C851	9590	☑		☑	☑					
C813	9653	☑	☑							
	9653	☑	☑							
C812	9652	☑	☑							
	9652	☑	☑							
C817	9661	☑	☑							
C810	9651	☑	☑							
C811	9663	☑	☑							
C811	9664	☑	☑							
C811	9665	☑	☑							
C811	9667	☑	☑							
C811	9650	☑	☑							
C817	9650	☑	☑							
C857	9719	☑		☑						
C860	9719	☑		☑						
C810	9659	☑	☑							

C915	9827	☑		☑	☑
C844	9718	☑		☑	☑
C866	9718	☑		☑	☑
C840	9700	☑		☑	☑
C841	9701	☑		☑	☑
	9700	☑		☑	☑
C910	9837	☑		☑	☑
C844	9705	☑		☑	☑
C865	9705	☑		☑	☑
C847	9702	☑		☑	☑
C862	9717	☑		☑	☑
C844	9724	☑		☑	☑
C844	9702	☑		☑	☑
C857	9714	☑		☑	☑
C845	9709	☑		☑	☑
C848	9709	☑		☑	☑
C913	9834	☑		☑	☑
C844	9591	☑		☑	☑
C845	9591	☑		☑	☑
C845	9590	☑		☑	☑
C845	8000	☑		☑	☑
C910	9835	☑		☑	
C864	9727	☑		☑	
C819	9650	☑	☑		
	9650	☑	☑		
C919	9820	☑		☑	
C832	9675	☑		☑	
C169	9591	☑		☑	
C300	9591	☑		☑	
C342	9591	☑		☑	
C445	9591	☑		☑	
C690	9591	☑		☑	
C73	9591	☑		☑	
C770	9591	☑		☑	
C773	9591	☑		☑	
C774	9591	☑		☑	
C779	9591	☑		☑	
C850	9591	☑		☑	
C859	9591	☑		☑	
C857	9590	☑		☑	
C859	9590	☑		☑	
C839	8000	☑		☑	
	9591	☑		☑	
C169	9590	☑			
C349	9590	☑			
C421	9590	☑			
C423	9590	☑			
C64	9590	☑			
C772	9590	☑			
C774	9590	☑			
C779	9590	☑			
	9590	☑			

SUPPLEMENTARY TABLE S3. Summary of the genetic instruments and the beta estimates for the association of each SNP with IGF-I and IGFBP-3 levels and with the different lymphoma subtypes

IGFBP-3

							EXPOSURE (IGF)		CLL/SLL			DLBCL			FL			MM		
Instrument	rs	chr	bp	Gene	Effect Allele	Other Allele	Beta	SE	Beta[1]	SE	p-value	Beta[1]	SE	p-value	Beta[1]	SE	p-value	Beta[1]	SE	p-value
IGFBP-3	rs4234798	4	7219933	*SORCS2*	G	T	0.095	0.011	-0.031	0.032	0.327	-0.001	0.031	0.980	-0.013	0.034	0.698	-0.015	0.042	0.720
IGFBP-3	rs11977526	7	46008110	*IGFBP3*	A	G	0.287	0.011	-0.007	0.032	0.821	0.005	0.031	0.873	-0.051	0.034	0.137	0.010	0.042	0.806
IGFBP-3	rs700753	7	46753684	*TNS3*	G	C	0.158	0.011	0.001	0.033	0.981	-0.024	0.032	0.452	-0.031	0.035	0.377	0.010	0.043	0.811
IGFBP-3	rs1065656	16	1838836	*NUBP2*	G	C	0.111	0.011	-0.006	0.034	0.858	0.028	0.033	0.391	0.016	0.036	0.651	-0.005	0.045	0.916

IGF-I

							EXPOSURE (IGF)		CLL/SLL			DLBCL			FL			MM		
Instrument	rs	chr	bp	Gene	Effect Allele	Other Allele	Beta	SE	Beta[1]	SE	p-value	Beta[1]	SE	p-value	Beta[1]	SE	p-value	Beta[1]	SE	p-value
IGF-I	rs903908	1	2202967	*SKI*	T	C	-0.016	0.003	-0.027	0.031	0.380	0.013	0.030	0.666	0.011	0.033	0.743	-0.026	0.041	0.518
IGF-I	rs17393144	1	9210262	*MIR34A*	G	A	-0.016	0.003	-0.031	0.034	0.359	-0.003	0.032	0.920	0.018	0.035	0.604	0.014	0.045	0.755
IGF-I	rs112436634	1	10637709	*PEX14*	C	T	-0.016	0.003	0.019	0.034	0.574	0.014	0.032	0.657	0.033	0.035	0.357	-0.041	0.044	0.346
IGF-I	rs17037452	1	11895675	*CLCN6*	A	G	0.023	0.003	0.020	0.043	0.647	0.064	0.042	0.122	-0.050	0.046	0.272	-0.037	0.056	0.503
IGF-I	rs36086195	1	16510894	*ARHGEF19/AS1*	T	C	-0.019	0.003	0.029	0.032	0.363	0.007	0.030	0.806	-0.004	0.033	0.895	0.030	0.041	0.461
IGF-I	rs12723255	1	21233570	*EIF4G3*	T	C	-0.017	0.003	-0.034	0.032	0.291	-0.059	0.031	0.053	-0.005	0.033	0.879	-0.003	0.042	0.941
IGF-I	rs6701954	1	22022176	*USP48*	T	G	0.014	0.003	-0.008	0.033	0.799	-0.006	0.031	0.852	0.027	0.034	0.431	0.097	0.042	0.020
IGF-I	rs76914895	1	23292603	*LACTBL1*	T	C	-0.027	0.005	0.087	0.071	0.218	0.094	0.066	0.152	-0.032	0.075	0.668	-0.005	0.083	0.950
IGF-I	rs2075995	1	23847464	*E2F2*	A	C	0.014	0.003	0.038	0.031	0.217	-0.019	0.030	0.511	-0.017	0.033	0.600	-0.092	0.041	0.025
IGF-I	rs2802330	1	26466831	*PDIK1L*	A	G	-0.031	0.003	0.018	0.041	0.666	0.054	0.040	0.174	-0.001	0.043	0.980	-0.037	0.054	0.499
IGF-I	rs17360994	1	27278573	*KDF1*	C	T	-0.042	0.005	-0.040	0.058	0.489	-0.001	0.056	0.980	0.020	0.061	0.742	-0.001	0.077	0.985
IGF-I	rs569356	1	29136686	*OPRD1*	A	G	-0.027	0.004	-0.048	0.045	0.288	-0.033	0.043	0.442	-0.002	0.047	0.964	-0.055	0.060	0.354
IGF-I	rs3131646	1	40383552	*MYCL*	G	T	-0.016	0.003	-0.037	0.035	0.290	0.003	0.033	0.938	-0.002	0.036	0.967	0.003	0.045	0.940
IGF-I	rs61780439	1	41490177	*SLFNL1—AS1*	G	A	-0.021	0.003	0.088	0.038	0.020	0.027	0.036	0.455	0.028	0.040	0.477	0.028	0.051	0.581
IGF-I	rs2819336	1	44015809	*PTPRF*	C	T	-0.027	0.003	-0.048	0.033	0.142	-0.001	0.031	0.972	-0.074	0.034	0.031	0.010	0.043	0.824
IGF-I	rs7539178	1	65383002	*JAK1*	A	C	-0.026	0.004	0.013	0.046	0.782	0.002	0.044	0.968	-0.020	0.048	0.683	0.014	0.059	0.813
IGF-I	rs1046011	1	65898996	*LEPR/LEPROT*	C	T	-0.021	0.003	-0.030	0.034	0.378	-0.004	0.033	0.899	0.053	0.036	0.147	-0.009	0.044	0.839
IGF-I	rs1430753	1	68692642	*WLS*	G	A	-0.021	0.003	-0.043	0.040	0.280	0.046	0.038	0.235	-0.034	0.042	0.418	-0.056	0.052	0.276
IGF-I	rs165316	1	91533297	*RPL5P6*	A	G	-0.073	0.003	-0.053	0.039	0.174	0.047	0.037	0.210	0.064	0.042	0.128	0.033	0.051	0.521
IGF-I	rs1825813	1	92708973	*C1orf146*	G	A	-0.023	0.003	-0.069	0.039	0.076	-0.014	0.037	0.708	-0.092	0.041	0.024	0.058	0.051	0.258
IGF-I	rs599839	1	109822166	*PSRC1/CELSR2*	A	G	-0.031	0.003	0.070	0.038	0.062	-0.004	0.036	0.922	-0.036	0.039	0.354	0.028	0.048	0.567
IGF-I	rs45505697	1	153651058	*NPR1*	C	A	0.03	0.005	-0.002	0.068	0.973	0.058	0.065	0.371	-0.086	0.072	0.230	0.091	0.094	0.335
IGF-I	rs1127313	1	154556425	*ADAR*	G	A	0.024	0.003	0.028	0.031	0.374	0.052	0.030	0.085	0.018	0.033	0.580	-0.009	0.040	0.824
IGF-I	rs77369503	1	163027266	*RGS4*	G	A	0.045	0.007	0.096	0.094	0.305	-0.048	0.089	0.589	0.030	0.101	0.764	-0.311	0.127	0.015
IGF-I	rs75681856	1	174916323	*RABGAP1L*	C	T	-0.023	0.004	-0.052	0.049	0.288	-0.031	0.046	0.494	-0.060	0.051	0.237	-0.022	0.064	0.739
IGF-I	rs12749024	1	176522365	*PAPPA2*	C	T	-0.075	0.004	0.045	0.049	0.356	0.073	0.046	0.119	0.051	0.052	0.325	-0.021	0.058	0.712
IGF-I	rs10913351	1	177447742	*AL122019.1*	G	A	-0.032	0.005	0.108	0.067	0.106	0.104	0.064	0.102	-0.033	0.071	0.639	0.043	0.084	0.607
IGF-I	rs11577063	1	179341999	*AXDND1*	G	T	-0.02	0.003	-0.024	0.037	0.528	-0.023	0.036	0.522	0.010	0.039	0.789	-0.013	0.049	0.794
IGF-I	rs143885630	1	183482785	*SMG7*	G	A	0.03	0.004	-0.017	0.050	0.741	0.029	0.048	0.552	0.074	0.052	0.156	-0.038	0.064	0.553
IGF-I	rs940400	1	200269134	*LINC00862*	C	A	0.025	0.004	-0.052	0.050	0.294	0.066	0.048	0.168	0.048	0.052	0.359	-0.015	0.065	0.814
IGF-I	rs7545345	1	205690941	*NUCKS1*	T	C	-0.026	0.004	-0.021	0.047	0.652	-0.032	0.044	0.469	-0.042	0.049	0.393	0.018	0.062	0.774
IGF-I	rs2724373	1	207999200	*C1orf132*	C	T	0.019	0.003	-0.040	0.033	0.234	-0.004	0.032	0.908	0.019	0.035	0.594	0.069	0.044	0.118
IGF-I	rs10779509	1	209728370	*AL023754.1*	T	C	-0.014	0.003	0.005	0.032	0.885	0.012	0.031	0.684	0.010	0.034	0.771	0.008	0.041	0.850
IGF-I	rs340837	1	214162734	*PROX1*	T	G	-0.021	0.003	-0.012	0.031	0.699	-0.017	0.030	0.570	0.005	0.033	0.869	0.100	0.041	0.015
IGF-I	rs12141189	1	221053545	*HLX*	C	T	-0.045	0.003	0.002	0.036	0.955	-0.044	0.035	0.210	0.045	0.038	0.244	-0.033	0.046	0.465
IGF-I	rs4306136	1	221608720	*AL360013.2*	A	G	0.017	0.003	-0.007	0.033	0.822	0.003	0.031	0.918	0.040	0.034	0.244	-0.006	0.041	0.884
IGF-I	rs708108	1	228189855	*WNT3A*	C	T	-0.015	0.003	-0.092	0.033	0.005	-0.031	0.031	0.314	0.002	0.035	0.958	0.052	0.042	0.210
IGF-I	rs684818	1	234854779	*AL160408.6*	T	C	0.024	0.003	0.027	0.032	0.400	0.034	0.030	0.269	0.007	0.034	0.838	0.025	0.041	0.533
IGF-I	rs7517340	1	243710190	*AKT3*	C	T	0.035	0.003	-0.022	0.041	0.583	-0.089	0.039	0.022	-0.031	0.043	0.467	0.012	0.052	0.823
IGF-I	rs35135518	2	16120506	*RN7SL104P*	T	C	0.029	0.004	0.019	0.055	0.727	0.028	0.052	0.595	0.086	0.056	0.126	-0.104	0.070	0.136
IGF-I	rs12710648	2	17989500	*SMC6*	A	G	0.017	0.003	0.034	0.032	0.283	0.002	0.030	0.941	0.057	0.034	0.087	-0.026	0.041	0.535
IGF-I	rs6760135	2	26088769	*ASXL2*	C	T	-0.05	0.003	0.016	0.038	0.678	0.041	0.036	0.256	-0.030	0.041	0.461	-0.044	0.049	0.363
IGF-I	rs1260326	2	27730940	*GCKR*	C	T	0.063	0.003	0.008	0.032	0.798	-0.015	0.030	0.621	-0.031	0.033	0.353	0.011	0.043	0.795
IGF-I	rs11677980	2	30522137	*LBH*	A	G	-0.015	0.003	0.035	0.034	0.306	0.038	0.033	0.248	0.059	0.036	0.103	-0.012	0.044	0.785
IGF-I	rs7574340	2	40621239	*SLC8A1*	C	T	-0.017	0.003	-0.102	0.034	0.003	-0.028	0.032	0.379	-0.022	0.036	0.536	0.031	0.044	0.481
IGF-I	rs6544549	2	42693056	*KCNG3*	T	C	0.024	0.004	-0.019	0.046	0.685	-0.033	0.044	0.449	-0.055	0.049	0.257	0.042	0.059	0.476
IGF-I	rs62136965	2	44347953	*snRNA*	T	C	-0.037	0.006	0.033	0.074	0.654	-0.109	0.072	0.130	0.123	0.080	0.126	-0.166	0.101	0.100

IGF-I (continued)

Instrument	rs	chr	bp	Gene	Effect Allele	Other Allele	EXPOSURE (IGF) Beta	SE	CLL/SLL Beta[1]	SE	p-value	DLBCL Beta[1]	SE	p-value	FL Beta[1]	SE	p-value	MM Beta[1]	SE	p-value
**IGF-I**	rs3791679	2	56096892	*EFEMP1*	A	G	-0.018	0.003	-0.017	0.037	0.649	0.009	0.035	0.803	-0.015	0.038	0.690	0.028	0.048	0.566
**IGF-I**	rs12471768	2	64928603	*SERTAD2*	C	T	0.022	0.003	-0.027	0.034	0.438	0.007	0.033	0.835	0.052	0.036	0.149	-0.018	0.045	0.694
**IGF-I**	rs702878	2	65702609	*AC007389.1*	A	G	0.014	0.003	-0.025	0.031	0.427	-0.004	0.030	0.889	0.004	0.033	0.906	0.051	0.041	0.214
**IGF-I**	rs35641591	2	70323994	*PCBP1—AS1*	C	T	0.05	0.006	-0.097	0.112	0.386	0.052	0.106	0.626	0.206	0.117	0.077	-0.069	0.113	0.542
**IGF-I**	rs6749680	2	73685852	*ALMS1*	A	G	0.015	0.003	-0.004	0.032	0.898	-0.017	0.031	0.588	-0.039	0.033	0.240	0.078	0.041	0.062
**IGF-I**	rs73954943	2	111890432	*BCL2L11*	G	A	-0.031	0.005	-0.178	0.066	0.006	-0.095	0.062	0.125	-0.021	0.069	0.756	0.022	0.083	0.793
**IGF-I**	rs7578633	2	113978650	*PAX8*	C	T	-0.018	0.003	0.019	0.033	0.559	0.012	0.031	0.704	0.013	0.034	0.696	-0.019	0.042	0.643
**IGF-I**	rs17050272	2	121306440	*AC073257.2*	G	A	0.024	0.003	0.024	0.032	0.449	-0.049	0.030	0.105	0.023	0.033	0.486	-0.054	0.041	0.184
**IGF-I**	rs58387407	2	152924773	*CACNB4*	A	G	-0.018	0.003	-0.021	0.039	0.582	-0.002	0.037	0.963	0.005	0.041	0.906	-0.024	0.049	0.626
**IGF-I**	rs2674492	2	172422338	*CYBRD1*	G	A	-0.014	0.003	0.021	0.032	0.516	-0.013	0.031	0.683	-0.030	0.034	0.382	0.039	0.043	0.358
**IGF-I**	rs17400325	2	178565913	*PDE11A*	C	T	0.054	0.006	-0.018	0.080	0.821	0.122	0.072	0.090	-0.109	0.083	0.191	0.148	0.107	0.164
**IGF-I**	rs6435156	2	203425475	*BMPR2*	C	T	0.024	0.003	-0.007	0.037	0.853	0.054	0.035	0.123	0.004	0.038	0.912	-0.028	0.047	0.558
**IGF-I**	rs1427676	2	204741166	*CTLA4*	T	C	0.015	0.003	-0.054	0.033	0.105	-0.017	0.032	0.591	-0.032	0.035	0.365	0.011	0.043	0.800
**IGF-I**	rs13418037	2	218314141	*DIRC3*	C	T	-0.02	0.003	-0.039	0.042	0.351	0.038	0.039	0.336	0.056	0.044	0.199	-0.074	0.051	0.149
**IGF-I**	rs62182127	2	219279588	*VIL1*	A	G	0.019	0.003	-0.061	0.032	0.054	0.042	0.031	0.174	0.055	0.034	0.101	-0.014	0.040	0.728
**IGF-I**	rs11678946	2	222302730	*EPHA4*	C	A	-0.014	0.003	-0.004	0.031	0.893	0.003	0.030	0.910	0.007	0.033	0.837	-0.005	0.040	0.893
**IGF-I**	rs4402747	2	225457173	*CUL3*	G	A	-0.016	0.003	0.031	0.031	0.323	-0.006	0.030	0.841	-0.018	0.033	0.592	-0.004	0.041	0.932
**IGF-I**	rs17323117	2	230162971	*PID1*	A	G	-0.029	0.005	0.047	0.057	0.416	-0.047	0.056	0.400	-0.046	0.061	0.456	0.055	0.079	0.481
**IGF-I**	rs1465529	2	231039037	*SP110*	T	C	0.019	0.003	-0.045	0.034	0.195	-0.006	0.033	0.851	-0.038	0.036	0.297	-0.023	0.044	0.607
**IGF-I**	rs6437249	2	242175331	*HDLBP*	C	T	0.019	0.003	-0.069	0.035	0.047	0.014	0.034	0.686	0.006	0.036	0.878	-0.067	0.044	0.130
**IGF-I**	rs7625680	3	11378069	*ATG7*	A	G	0.015	0.003	-0.014	0.032	0.669	-0.005	0.031	0.874	0.033	0.034	0.332	0.026	0.042	0.544
**IGF-I**	rs1822825	3	12449963	*PPARG*	A	G	-0.014	0.003	0.008	0.031	0.806	0.015	0.030	0.612	0.002	0.033	0.942	0.017	0.041	0.673
**IGF-I**	rs2607748	3	14158725	*CHCHD4*	T	C	-0.017	0.003	0.002	0.032	0.956	-0.042	0.030	0.166	-0.024	0.033	0.480	-0.036	0.041	0.377
**IGF-I**	rs11717397	3	23368583	*UBE2E2*	G	A	0.015	0.003	0.055	0.032	0.079	-0.020	0.030	0.502	0.010	0.033	0.772	-0.049	0.041	0.230
**IGF-I**	rs2362755	3	24716668	*THRB-AS1*	G	T	-0.016	0.003	0.016	0.032	0.608	0.028	0.030	0.361	-0.003	0.034	0.940	0.031	0.041	0.442
**IGF-I**	rs11928797	3	33457493	*UBP1*	A	C	0.03	0.004	-0.036	0.051	0.484	0.022	0.048	0.652	0.008	0.055	0.884	0.102	0.067	0.126
**IGF-I**	rs33969824	3	42679777	*NKTR*	G	T	0.02	0.004	0.068	0.046	0.145	0.008	0.045	0.863	0.040	0.049	0.414	0.015	0.060	0.806
**IGF-I**	rs12491473	3	46989904	*CCDC12*	G	A	0.02	0.003	0.080	0.032	0.011	0.044	0.030	0.148	0.060	0.033	0.071	0.070	0.041	0.088
**IGF-I**	rs2228561	3	48628014	*COL7A1*	A	G	0.02	0.004	-0.150	0.046	0.001	-0.060	0.044	0.167	-0.089	0.048	0.066	0.009	0.061	0.878
**IGF-I**	rs4768	3	49758764	*RNF123*	A	G	-0.015	0.003	-0.046	0.034	0.173	-0.074	0.032	0.021	-0.062	0.035	0.079	0.046	0.044	0.297
**IGF-I**	rs9809209	3	51281664	*DOCK3*	G	A	-0.017	0.003	0.053	0.033	0.115	0.045	0.032	0.155	0.047	0.035	0.178	0.050	0.042	0.236
**IGF-I**	rs112893170	3	57211863	*IL17RD*	T	C	0.02	0.003	0.013	0.043	0.759	0.006	0.041	0.891	0.020	0.045	0.658	-0.080	0.054	0.135
**IGF-I**	rs7628689	3	88216647	*C3orf38*	G	A	0.029	0.003	-0.071	0.045	0.112	-0.040	0.043	0.347	-0.103	0.047	0.028	-0.102	0.057	0.075
**IGF-I**	rs3772102	3	98502628	*ST3GAL6*	T	G	-0.02	0.003	0.036	0.031	0.259	-0.005	0.030	0.876	0.089	0.033	0.007	-0.024	0.041	0.555
**IGF-I**	rs62280667	3	101084604	*SENP7*	T	C	-0.028	0.003	0.016	0.033	0.634	-0.042	0.032	0.180	-0.024	0.035	0.492	-0.005	0.043	0.910
**IGF-I**	rs62263345	3	107252190	*BBX*	A	G	0.028	0.004	0.008	0.048	0.873	0.023	0.046	0.622	0.002	0.051	0.968	0.139	0.061	0.022
**IGF-I**	rs13069961	3	124358715	*KALRN*	A	G	-0.018	0.003	-0.026	0.038	0.504	-0.093	0.037	0.011	-0.021	0.040	0.595	-0.059	0.050	0.237
**IGF-I**	rs687339	3	135932359	*AC092991.1*	T	C	0.04	0.003	0.011	0.038	0.762	-0.038	0.036	0.287	-0.011	0.039	0.773	0.067	0.049	0.169
**IGF-I**	rs811332	3	138078348	*MRAS*	C	T	0.019	0.003	-0.077	0.039	0.049	0.020	0.038	0.587	0.051	0.042	0.221	0.058	0.052	0.265
**IGF-I**	rs55717031	3	138848505	*MRPS22*	G	T	0.032	0.003	-0.041	0.034	0.232	0.027	0.033	0.421	-0.052	0.036	0.146	0.003	0.044	0.953
**IGF-I**	rs6440008	3	141154542	*ZBTB38*	T	C	0.035	0.003	-0.053	0.033	0.108	0.013	0.032	0.680	0.035	0.035	0.318	0.043	0.042	0.305
**IGF-I**	rs73238159	3	142078759	*XRN1*	T	C	-0.025	0.004	-0.067	0.046	0.139	0.025	0.044	0.563	-0.091	0.049	0.060	0.020	0.061	0.740
**IGF-I**	rs13073970	3	170630520	*EIF5A2*	G	T	-0.025	0.003	-0.026	0.039	0.505	0.037	0.037	0.312	0.080	0.040	0.048	-0.052	0.050	0.301
**IGF-I**	rs56062334	3	172299226	*LINC02068*	T	C	0.017	0.003	-0.028	0.033	0.403	-0.016	0.032	0.621	-0.033	0.035	0.340	-0.009	0.041	0.817
**IGF-I**	rs9819762	3	178914879	*PIK3CA*	T	C	0.019	0.003	-0.011	0.040	0.782	-0.060	0.038	0.118	-0.110	0.042	0.009	0.004	0.051	0.937
**IGF-I**	rs66707192	3	186382065	*HRG*	G	A	0.018	0.003	0.016	0.036	0.654	-0.037	0.034	0.278	0.010	0.037	0.789	0.029	0.047	0.542
**IGF-I**	rs13108218	4	3443931	*HGFAC*	G	A	0.017	0.003	-0.021	0.033	0.533	-0.064	0.031	0.042	-0.015	0.035	0.658	-0.046	0.041	0.270
**IGF-I**	rs1055582	4	39700173	*UBE2K*	C	T	0.027	0.003	0.041	0.032	0.199	0.016	0.030	0.587	0.044	0.033	0.180	0.005	0.041	0.912
**IGF-I**	rs62302688	4	46448465	*GABRA2*	G	A	0.039	0.004	0.018	0.071	0.796	0.080	0.066	0.227	-0.044	0.075	0.556	0.037	0.076	0.624
**IGF-I**	rs115805235	4	69764890	*AC021146.3*	C	T	0.039	0.006	-0.058	0.077	0.455	0.109	0.074	0.140	-0.129	0.082	0.118		NA	
**IGF-I**	rs16845929	4	72017058	*SLC4A4*	C	T	0.032	0.005	-0.086	0.067	0.198	-0.035	0.064	0.587	0.037	0.071	0.597	-0.205	0.079	0.010
**IGF-I**	rs2280099	4	90035549	*TIGD2*	G	A	0.025	0.003	-0.023	0.041	0.573	-0.002	0.039	0.963	0.016	0.043	0.712	0.026	0.054	0.630
**IGF-I**	rs35036084	4	97552791	*LINC02267*	T	C	0.017	0.003	-0.019	0.033	0.552	0.040	0.031	0.201	0.015	0.034	0.657	-0.026	0.041	0.535
**IGF-I**	rs6532798	4	100054827	*ADH4*	T	C	0.037	0.003	-0.009	0.034	0.784	0.001	0.032	0.968	-0.010	0.035	0.787	-0.065	0.045	0.148
**IGF-I**	rs62342064	4	104665972	*TACR3*	C	T	0.022	0.004	0.050	0.056	0.366	-0.063	0.054	0.247	-0.044	0.060	0.461	0.103	0.061	0.096
**IGF-I**	rs17429745	4	106038169	*AC096577.1*	G	T	0.026	0.003	0.020	0.033	0.558	0.021	0.032	0.514	0.011	0.035	0.756	0.023	0.043	0.605
**IGF-I**	rs3804173	4	121719923	*PRDMS*	A	G	-0.02	0.003	-0.052	0.033	0.122	-0.018	0.032	0.578	-0.038	0.035	0.280	0.023	0.044	0.605
**IGF-I**	rs111443396	4	124773202	*LINC01091*	T	C	-0.026	0.004	0.011	0.048	0.816	-0.035	0.046	0.447	0.052	0.050	0.302	0.023	0.065	0.729
**IGF-I**	rs7667562	4	129133826	*LARP1B*	C	A	0.016	0.003	0.017	0.035	0.631	0.017	0.034	0.604	0.010	0.037	0.791	-0.026	0.045	0.574
**IGF-I**	rs6827641	4	145653694	*GYPA/HHIP*	C	T	-0.014	0.003	0.046	0.031	0.144	-0.004	0.030	0.885	0.027	0.033	0.420	0.033	0.041	0.419
**IGF-I**	rs6853741	4	148982559	*ARHGAP10*	A	G	0.024	0.003	-0.030	0.036	0.405	0.016	0.034	0.645	-0.058	0.038	0.120	-0.031	0.046	0.492
**IGF-I**	rs62334147	4	169345005	*DDX60L*	T	C	-0.019	0.003	0.022	0.040	0.588	0.026	0.039	0.499	-0.022	0.043	0.612	-0.042	0.053	0.420
**IGF-I**	rs4394044	4	186607420	*SORBS2*	T	C	0.014	0.003	-0.036	0.032	0.255	0.002	0.030	0.951	-0.095	0.033	0.004	0.050	0.041	0.229
**IGF-I**	rs9292578	5	35230075	*PRLR*	C	A	0.04	0.006	0.044	0.077	0.565	-0.010	0.074	0.894	-0.175	0.082	0.034	0.181	0.100	0.071
**IGF-I**	rs6895953	5	39084471	*RICTOR*	G	A	0.024	0.003	-0.028	0.032	0.373	0.001	0.030	0.965	0.013	0.033	0.699	-0.065	0.041	0.113

IGF-I (continued)

							EXPOSURE (IGF)		CLL/SLL			DLBCL			FL			MM		
Instrument	rs	chr	bp	Gene	Effect Allele	Other Allele	Beta	SE	Beta[1]	SE	p-value	Beta[1]	SE	p-value	Beta[1]	SE	p-value	Beta[1]	SE	p-value
**IGF-I**	rs72758321	5	41464841	*PLCXD3*	A	G	-0.047	0.006	-0.126	0.095	0.183	-0.059	0.092	0.524	0.148	0.099	0.134	0.091	0.102	0.373
**IGF-I**	rs6180	5	42719239	*GHR*	C	A	-0.035	0.003	0.050	0.032	0.114	0.000	0.030	0.988	0.029	0.033	0.389	0.078	0.041	0.057
**IGF-I**	rs12520263	5	44122508	*RNU6-381P*	G	T	-0.017	0.003	-0.004	0.036	0.916	-0.008	0.034	0.825	0.067	0.037	0.071	-0.006	0.045	0.896
**IGF-I**	rs7719168	5	53292390	*ARL15*	A	C	-0.03	0.004	-0.033	0.051	0.524	0.058	0.049	0.234	0.046	0.054	0.392	0.075	0.065	0.244
**IGF-I**	rs28650790	5	55861464	*C5orf67*	C	T	-0.018	0.003	-0.001	0.040	0.974	0.026	0.039	0.505	-0.033	0.043	0.445	-0.003	0.052	0.961
**IGF-I**	rs1498603	5	58333125	*PDE4D*	T	G	0.031	0.005	-0.035	0.067	0.598	-0.060	0.064	0.354	0.061	0.070	0.388	0.023	0.080	0.775
**IGF-I**	rs11954036	5	59028853	*PDE4D*	T	C	0.037	0.003	-0.044	0.033	0.181	-0.042	0.032	0.185	-0.017	0.035	0.632	0.025	0.043	0.561
**IGF-I**	rs80170948	5	64020316	*SREK1IP1*	T	G	-0.039	0.006	0.036	0.093	0.701	-0.075	0.089	0.401	-0.083	0.096	0.386	0.027	0.104	0.799
**IGF-I**	rs2227819	5	76012745	*F2R*	C	T	-0.022	0.004	0.015	0.055	0.790	0.099	0.053	0.061	0.119	0.058	0.038	-0.049	0.068	0.472
**IGF-I**	rs12108803	5	77158507	*TBCA*	T	G	-0.033	0.006	0.073	0.078	0.349	0.052	0.078	0.503	0.120	0.082	0.145	-0.123	0.094	0.192
**IGF-I**	rs840809	5	87173927	*TMEM161B*	A	C	0.016	0.003	-0.026	0.035	0.464	-0.001	0.033	0.972	0.039	0.037	0.296	-0.048	0.045	0.283
**IGF-I**	rs13178887	5	88355993	*MEF2C-AS1*	T	C	0.023	0.003	-0.007	0.032	0.829	-0.027	0.031	0.383	-0.084	0.034	0.014	0.000	0.041	0.994
**IGF-I**	rs2366398	5	89437963	*LINC01339*	G	T	-0.018	0.003	-0.005	0.037	0.895	0.014	0.035	0.681	-0.010	0.039	0.794	-0.023	0.048	0.636
**IGF-I**	rs26822	5	102518795	*PPIP5K2*	A	G	-0.017	0.003	0.002	0.034	0.960	0.044	0.032	0.177	0.027	0.036	0.456	-0.087	0.044	0.048
**IGF-I**	rs73271090	5	132313550	*AC010240.1*	G	A	0.044	0.003	-0.067	0.044	0.127	-0.038	0.041	0.363	-0.050	0.046	0.282	0.047	0.056	0.397
**IGF-I**	rs329122	5	133864599	*JADE2*	G	A	-0.018	0.003	-0.003	0.031	0.933	-0.038	0.030	0.207	-0.012	0.033	0.722	-0.049	0.041	0.230
**IGF-I**	rs11242236	5	134586980	*C5orf66*	G	A	0.025	0.003	-0.018	0.032	0.563	-0.047	0.030	0.124	-0.008	0.033	0.800	0.019	0.041	0.645
**IGF-I**	rs2348604	5	136809831	*SPOCK1*	T	C	-0.016	0.003	-0.055	0.036	0.125	-0.052	0.034	0.126	-0.023	0.038	0.538	0.024	0.046	0.612
**IGF-I**	rs3734166	5	137665323	*CDC25C*	A	G	0.028	0.003	-0.004	0.035	0.902	0.046	0.033	0.166	0.015	0.037	0.681	0.014	0.046	0.759
**IGF-I**	rs2042253	5	143059433	*MIR5197*	T	C	0.023	0.003	0.016	0.036	0.667	0.013	0.035	0.715	0.021	0.038	0.583	0.020	0.047	0.670
**IGF-I**	rs35668185	5	168256455	*SLIT3*	T	C	0.056	0.003	-0.004	0.039	0.918	-0.015	0.037	0.696	-0.015	0.041	0.707	0.008	0.051	0.882
**IGF-I**	rs13168379	5	173382761	*CPEB4*	G	A	-0.031	0.005	-0.002	0.061	0.971	-0.022	0.058	0.703	-0.085	0.064	0.187	-0.001	0.076	0.994
**IGF-I**	rs17714046	5	180661980	*TRIM41*	C	T	0.042	0.006	-0.106	0.076	0.162	-0.003	0.072	0.967	-0.058	0.079	0.465		NA	
**IGF-I**	rs584955	6	7097141	*RREB1*	A	G	0.036	0.006	-0.015	0.078	0.848	-0.078	0.075	0.297	-0.046	0.082	0.579	-0.127	0.095	0.182
**IGF-I**	rs2296198	6	18399750	*RNF144B*	C	T	0.016	0.003	0.050	0.037	0.171	0.019	0.035	0.583	0.032	0.039	0.405	0.030	0.047	0.512
**IGF-I**	rs72828596	6	19183591	*AL589647.1*	G	A	-0.019	0.004	-0.075	0.047	0.116	-0.008	0.045	0.869	-0.069	0.051	0.170	-0.017	0.061	0.783
**IGF-I**	rs73382439	6	20404420	*E2F3*	C	T	0.019	0.003	-0.038	0.041	0.354	-0.025	0.039	0.519	-0.033	0.043	0.444	-0.003	0.052	0.959
**IGF-I**	rs17258904	6	21928131	*CASC15*	A	G	0.017	0.003	-0.018	0.035	0.620	0.078	0.034	0.021	0.068	0.037	0.066	-0.011	0.046	0.803
**IGF-I**	rs1165196	6	25813150	*SLC17A1*	A	G	-0.029	0.003	-0.021	0.031	0.506	-0.015	0.030	0.612	-0.076	0.033	0.021	-0.037	0.042	0.369
**IGF-I**	rs13195402	6	26463575	*BTN2A1*	T	G	-0.038	0.004	0.049	0.062	0.436	0.043	0.086	0.620	-0.012	0.065	0.853	-0.039	0.109	0.718
**IGF-I**	rs16897515	6	27278020	*POM121L2*	A	C	-0.023	0.003	0.112	0.042	0.008	0.067	0.046	0.144	0.042	0.044	0.336	0.003	0.063	0.960
**IGF-I**	rs33932084	6	28268824	*PGBD1*	G	A	-0.034	0.004	0.050	0.055	0.365	0.067	0.074	0.367	-0.026	0.058	0.651	-0.075	0.108	0.491
**IGF-I**	rs9267488	6	31514247	*ATP6V1/G2 DDX39B/ ATP6V1G2*	G	A	-0.031	0.004	0.187	0.049	0.000	0.213	0.053	0.000	0.009	0.052	0.866	0.002	0.097	0.983
**IGF-I**	rs1150752	6	32064726	*TNXB*	C	T	-0.031	0.004	0.234	0.050	0.000	0.180	0.054	0.001	-0.062	0.054	0.250	0.009	0.097	0.928
**IGF-I**	rs12194618	6	38091030	*ZFAND*	G	A	-0.017	0.003	-0.025	0.032	0.434	-0.004	0.031	0.899	-0.071	0.034	0.039	0.087	0.042	0.040
**IGF-I**	rs7740433	6	42908013	*CNPY3*	A	G	0.017	0.003	-0.057	0.037	0.122	0.054	0.035	0.121	-0.047	0.039	0.229	0.042	0.048	0.383
**IGF-I**	rs998584	6	43757896	*VEGFA*	A	C	0.02	0.003	0.019	0.034	0.582	0.064	0.032	0.047	-0.005	0.036	0.894	0.006	0.040	0.884
**IGF-I**	rs6924225	6	45584732	*RUNX2*	G	A	0.019	0.003	-0.007	0.043	0.871	0.049	0.041	0.229	-0.016	0.046	0.726	-0.040	0.057	0.481
**IGF-I**	rs2397112	6	52684333	*GSTA6*	A	G	0.019	0.003	0.009	0.032	0.786	-0.052	0.030	0.086	-0.005	0.033	0.881	-0.001	0.042	0.989
**IGF-I**	rs9361489	6	79816785	*PHIP*	T	C	0.022	0.003	0.009	0.032	0.782	-0.006	0.030	0.849	0.003	0.033	0.925	-0.003	0.041	0.941
**IGF-I**	rs6916994	6	87991236	*GJB7*	C	T	0.029	0.003	0.031	0.031	0.321	-0.005	0.030	0.863	-0.061	0.033	0.064	-0.067	0.040	0.094
**IGF-I**	rs670049	6	100087024	*PRDM1*	A	C	0.019	0.003	0.019	0.033	0.578	0.001	0.032	0.980	0.002	0.035	0.949	-0.002	0.044	0.969
**IGF-I**	rs9322822	6	105369598	*LIN28B—AS1*	C	T	0.015	0.003	0.014	0.034	0.691	0.026	0.032	0.431	-0.065	0.036	0.070	-0.037	0.043	0.398
**IGF-I**	rs4946810	6	107420270	*BEND3*	A	C	-0.016	0.003	0.033	0.033	0.307	0.000	0.031	0.991	0.022	0.035	0.525	-0.029	0.042	0.489
**IGF-I**	rs218291	6	108467024	*OSTM1*	G	A	0.016	0.003	0.019	0.033	0.566	-0.022	0.031	0.483	-0.013	0.034	0.699	0.049	0.043	0.257
**IGF-I**	rs9398171	6	108983527	*FOXO3*	T	C	0.05	0.003	-0.008	0.034	0.814	-0.038	0.033	0.249	-0.034	0.036	0.343	0.071	0.044	0.110
**IGF-I**	rs41285260	6	126661502	*CENPW*	T	G	0.039	0.004	0.062	0.061	0.308	0.062	0.058	0.288	0.047	0.064	0.467	0.098	0.073	0.181
**IGF-I**	rs9321106	6	128355316	*PTPRK*	A	G	0.018	0.003	0.107	0.043	0.013	0.042	0.042	0.312	0.037	0.045	0.416	-0.019	0.055	0.731
**IGF-I**	rs9398891	6	129314749	*LAMA2*	C	T	-0.017	0.003	-0.020	0.034	0.559	0.032	0.032	0.323	-0.023	0.035	0.513	-0.011	0.044	0.806
**IGF-I**	rs3890746	6	130371055	*L3MBTL3*	T	C	-0.02	0.003	-0.005	0.031	0.875	0.008	0.030	0.788	0.009	0.033	0.787	0.027	0.041	0.513
**IGF-I**	rs2786185	6	147595554	*STXBPS*	G	A	0.019	0.003	0.001	0.031	0.981	0.027	0.030	0.376	0.054	0.033	0.099	0.002	0.041	0.955
**IGF-I**	rs7774230	6	152164239	*ESR1*	C	T	-0.026	0.003	0.028	0.031	0.376	0.036	0.030	0.239	0.038	0.033	0.250	-0.018	0.041	0.656
**IGF-I**	rs790513	6	154420368	*OPRM1*	C	A	0.025	0.003	-0.003	0.036	0.930	0.013	0.035	0.703	-0.056	0.038	0.144	-0.047	0.047	0.322
**IGF-I**	rs7758644	6	156583467	*snoRNA*	C	A	-0.019	0.003	0.020	0.042	0.641	0.040	0.041	0.333	0.002	0.045	0.967	-0.024	0.055	0.672
**IGF-I**	rs3127579	6	160674632	*SLC22A2*	G	A	-0.033	0.004	0.024	0.048	0.609	-0.011	0.046	0.819	0.006	0.050	0.898	-0.074	0.063	0.240
**IGF-I**	rs12211977	6	161252770	*lincRNA*	G	A	-0.023	0.004	-0.075	0.054	0.162	-0.064	0.052	0.218	-0.006	0.057	0.917	-0.059	0.072	0.409
**IGF-I**	rs504371	6	165724052	*C6orf118*	C	A	-0.015	0.003	0.043	0.033	0.189	0.054	0.032	0.087	0.035	0.035	0.313	-0.028	0.043	0.513
**IGF-I**	rs4709995	6	166313447	*PDE10A*	C	T	-0.042	0.003	-0.002	0.032	0.960	0.072	0.031	0.018	0.043	0.034	0.201	0.040	0.042	0.332
**IGF-I**	rs7802508	7	1191689	*ZFAND2A*	G	A	-0.021	0.003	0.013	0.032	0.684	-0.005	0.031	0.875	-0.035	0.034	0.297	-0.026	0.042	0.530
**IGF-I**	rs12699547	7	2015970	*MAD1L1*	C	T	0.021	0.003	-0.031	0.033	0.337	-0.019	0.031	0.545	0.003	0.034	0.940	0.014	0.042	0.738
**IGF-I**	rs1182174	7	2875420	*GNA12*	G	A	-0.021	0.003	0.035	0.034	0.304	-0.010	0.033	0.771	0.034	0.036	0.348	0.031	0.045	0.479
**IGF-I**	rs2250243	7	6690240	*ZNF316*	T	C	-0.024	0.003	0.018	0.037	0.619	0.040	0.035	0.252	-0.005	0.039	0.903	0.005	0.047	0.908
**IGF-I**	rs4719393	7	14219213	*DGKB*	T	G	0.027	0.003	0.038	0.034	0.259	-0.043	0.033	0.182	0.061	0.036	0.089	0.003	0.044	0.951
**IGF-I**	rs10252510	7	31023108	*GHRHR*	G	A	0.02	0.003	0.024	0.035	0.496	-0.033	0.033	0.320	0.020	0.036	0.592	0.012	0.044	0.780
**IGF-I**	rs7790246	7	32976416	*AVL9/RP9P*	C	T	-0.017	0.003	-0.009	0.035	0.788	0.026	0.033	0.432	-0.019	0.037	0.598	-0.046	0.044	0.299
**IGF-I**	rs1050327	7	44808017	*ZMIZ2*	G	A	-0.017	0.003	-0.013	0.032	0.684	0.051	0.030	0.089	0.007	0.033	0.827	0.052	0.041	0.207
**IGF-I**	rs870796	7	45426435	*ELK1P1*	G	A	0.017	0.003	0.063	0.032	0.049	0.006	0.031	0.835	-0.018	0.034	0.600	0.000	0.041	0.993
**IGF-I**	rs2270628	7	45949570	*IGFBP3*	C	T	-0.033	0.003	-0.056	0.038	0.144	-0.014	0.037	0.711	-0.066	0.040	0.101	0.046	0.051	0.369
**IGF-I**	rs74657816	7	46670682	*HMGN1P19*	T	G	0.047	0.005	0.032	0.074	0.661	0.051	0.069	0.460	-0.037	0.078	0.637	-0.130	0.086	0.129
**IGF-I**	rs12538762	7	47264328	*TNS3*	C	T	0.026	0.004	-0.025	0.051	0.627	0.016	0.049	0.746	-0.013	0.054	0.814	0.221	0.066	0.001

IGF-I (continued)

Instrument	rs	chr	bp	Gene	Effect Allele	Other Allele	EXPOSURE (IGF) Beta	SE	CLL/SLL Beta[1]	SE	p-value	DLBCL Beta[1]	SE	p-value	FL Beta[1]	SE	p-value	MM Beta[1]	SE	p-value
IGF-I	rs6974707	7	55982894	ZNF713	G	A	-0.019	0.003	-0.061	0.038	0.104	-0.017	0.036	0.634	-0.058	0.039	0.143	0.031	0.049	0.514
IGF-I	rs35862187	7	69625029	AUTS2	A	G	0.031	0.006	0.044	0.073	0.545	-0.052	0.071	0.464	0.025	0.076	0.746	0.031	0.091	0.738
IGF-I	rs17145738	7	72982874	TBM	C	T	-0.034	0.004	-0.010	0.050	0.838	0.071	0.048	0.143	0.084	0.052	0.109	-0.006	0.063	0.925
IGF-I	rs411717	7	94033031	COL1A2	C	T	-0.015	0.003	-0.021	0.031	0.504	-0.008	0.030	0.799	-0.002	0.033	0.964	-0.025	0.041	0.553
IGF-I	rs34670419	7	99130834	ZKSCANS	G	T	-0.036	0.006	0.064	0.083	0.439	-0.079	0.081	0.331	-0.006	0.088	0.947	0.083	0.109	0.445
IGF-I	rs34312198	7	99674870	ZNF3	C	A	-0.024	0.004	0.052	0.051	0.306	0.024	0.049	0.620	-0.054	0.054	0.314	-0.013	0.066	0.845
IGF-I	rs7783012	7	114116881	FOXP2	A	G	-0.016	0.003	0.065	0.032	0.040	-0.044	0.030	0.144	0.041	0.033	0.219	0.044	0.041	0.279
IGF-I	rs12666306	7	115082406	AC073901.1	G	A	0.017	0.003	0.057	0.031	0.066	0.034	0.030	0.252	0.087	0.033	0.008	0.044	0.041	0.282
IGF-I	rs2896395	7	127511705	SND1	C	T	0.015	0.003	0.007	0.034	0.830	0.034	0.033	0.291	0.050	0.036	0.161	0.017	0.044	0.699
IGF-I	rs11556924	7	129663496	ZC3HC1	T	C	-0.016	0.003	0.077	0.033	0.019	0.026	0.032	0.419	0.028	0.035	0.421	0.036	0.042	0.396
IGF-I	rs207212	7	130547217	LINC00513	C	T	0.028	0.004	0.004	0.064	0.954	-0.058	0.062	0.355	0.044	0.069	0.521	0.060	0.067	0.368
IGF-I	rs1986692	7	133743393	EXOC4	A	G	-0.015	0.003	0.003	0.033	0.916	-0.017	0.032	0.599	0.005	0.035	0.874	-0.012	0.042	0.767
IGF-I	rs273956	7	137603188	CREB3L2	G	A	-0.021	0.003	0.004	0.033	0.903	0.028	0.031	0.372	0.004	0.035	0.913	-0.016	0.041	0.691
IGF-I	rs114949263	7	150498245	TMEM176B/ TMEM 176A	T	C	0.027	0.004	-0.079	0.051	0.120	-0.146	0.049	0.003	-0.006	0.053	0.913	0.050	0.066	0.454
IGF-I	rs10246481	7	156184748	lincRNA	A	G	-0.015	0.003	0.013	0.032	0.692	-0.046	0.031	0.132	-0.030	0.034	0.370	-0.004	0.041	0.928
IGF-I	rs9657541	8	10643164	SOX7/PINX1/	C	T	0.02	0.003	-0.005	0.039	0.906	-0.034	0.038	0.368	0.067	0.041	0.102	0.089	0.053	0.091
IGF-I	rs76393968	8	16282937	MSR1	G	A	0.06	0.01	-0.120	0.147	0.413	-0.057	0.134	0.670	0.016	0.153	0.916		NA	
IGF-I	rs1495741	8	18272881	NAT2	A	G	-0.026	0.003	-0.038	0.038	0.312	-0.014	0.036	0.696	-0.110	0.039	0.005	-0.006	0.048	0.898
IGF-I	rs11782452	8	26361601	BNIP3L	G	A	0.015	0.003	-0.025	0.032	0.429	-0.018	0.030	0.556	-0.015	0.033	0.659	-0.077	0.041	0.064
IGF-I	rs56352849	8	73769173	KCNB2	G	A	-0.016	0.003	-0.001	0.035	0.977	0.014	0.034	0.665	0.044	0.037	0.231	-0.064	0.045	0.157
IGF-I	rs1431015	8	77131580	HNF4G	C	T	0.02	0.003	0.032	0.032	0.326	0.015	0.031	0.618	-0.009	0.034	0.801	0.015	0.042	0.715
IGF-I	rs6473015	8	78178485	lincRNA	A	C	-0.019	0.003	0.007	0.034	0.834	0.014	0.033	0.661	-0.031	0.036	0.396	0.093	0.045	0.041
IGF-I	rs445036	8	81408409	ZBTB10	T	C	0.019	0.003	0.018	0.034	0.593	0.007	0.033	0.822	0.017	0.036	0.639	0.013	0.044	0.769
IGF-I	rs1786342	8	101676363	SNX31	T	C	0.017	0.003	-0.028	0.032	0.378	-0.063	0.031	0.041	-0.023	0.034	0.503	0.053	0.042	0.206
IGF-I	rs60862542	8	109275071	EIF3E	G	A	0.017	0.003	0.019	0.038	0.607	0.006	0.036	0.861	-0.009	0.040	0.822	-0.019	0.049	0.702
IGF-I	rs2737205	8	116610180	TRPS1	C	T	-0.023	0.003	-0.019	0.032	0.544	-0.025	0.030	0.410	-0.028	0.033	0.408	-0.014	0.041	0.729
IGF-I	rs2978062	8	134571618	ST3GAL1	T	G	-0.019	0.003	0.015	0.044	0.736	0.023	0.042	0.586	-0.019	0.047	0.687	0.037	0.055	0.502
IGF-I	rs716100	8	135661278	ZFAT	G	A	-0.019	0.003	-0.031	0.034	0.359	0.006	0.032	0.857	-0.039	0.035	0.268	0.009	0.043	0.839
IGF-I	rs12549853	8	145020636	PLEC	G	A	-0.016	0.003	-0.024	0.032	0.449	-0.027	0.031	0.384	-0.006	0.034	0.855	-0.066	0.042	0.118
IGF-I	rs10114121	9	19440136	ACER2	G	A	-0.02	0.004	0.023	0.044	0.604	0.009	0.042	0.833	0.003	0.046	0.944	0.140	0.057	0.013
IGF-I	rs10757291	9	22161884	CDKN2B/AS1	A	G	-0.019	0.003	-0.118	0.031	0.000	-0.001	0.030	0.977	-0.042	0.033	0.201	-0.029	0.040	0.478
IGF-I	rs10811787	9	22871816	AL391117.1	T	C	-0.015	0.003	-0.020	0.031	0.532	-0.006	0.030	0.855	-0.011	0.033	0.740	0.051	0.041	0.214
IGF-I	rs11557154	9	34107505	DCAF12	T	C	0.024	0.004	0.046	0.047	0.334	0.051	0.045	0.259	0.164	0.049	0.001	-0.019	0.059	0.748
IGF-I	rs10869022	9	74057313	TRPM3	C	T	0.021	0.003	-0.048	0.039	0.225	-0.013	0.038	0.718	-0.091	0.042	0.030	0.122	0.050	0.014
IGF-I	rs2378662	9	86707289	AL390838.1	A	G	-0.017	0.003	0.058	0.032	0.068	-0.003	0.030	0.928	-0.016	0.033	0.628	-0.019	0.041	0.638
IGF-I	rs10908903	9	92228559	GADD45G	T	G	0.015	0.003	-0.014	0.031	0.645	-0.038	0.030	0.205	0.035	0.033	0.285	0.020	0.040	0.622
IGF-I	rs1055710	9	96214928	FAM/120AOS	A	G	-0.018	0.003	0.032	0.033	0.334	0.021	0.032	0.510	0.010	0.035	0.770	-0.135	0.043	0.002
IGF-I	rs75660441	9	97662448	C9orf3	A	G	0.039	0.005	0.047	0.065	0.469	-0.046	0.063	0.464	-0.103	0.069	0.137	0.027	0.085	0.757
IGF-I	rs28831479	9	98254526	PTCH1	C	A	0.022	0.003	0.010	0.036	0.775	-0.017	0.034	0.614	-0.017	0.037	0.652	-0.021	0.046	0.655
IGF-I	rs7034716	9	101858382	TGFBR1	C	T	0.015	0.003	-0.026	0.035	0.455	0.040	0.033	0.229	-0.026	0.036	0.469		NA	
IGF-I	rs6479003	9	102948685	INVS	G	A	0.024	0.004	-0.032	0.051	0.525	-0.052	0.049	0.290	-0.047	0.054	0.382	0.061	0.070	0.385
IGF-I	rs41277821	9	109689972	ZNF462	T	C	0.062	0.009	0.190	0.135	0.158	0.085	0.129	0.508	-0.108	0.144	0.452	-0.098	0.161	0.542
IGF-I	rs7872812	9	119341544	ASTN2	C	T	-0.026	0.004	-0.026	0.044	0.546	0.044	0.042	0.298	-0.022	0.047	0.638	0.037	0.057	0.510
IGF-I	rs13301073	9	128284378	MAPKAP1	G	A	0.022	0.003	0.049	0.033	0.137	-0.043	0.031	0.165	0.007	0.034	0.849	0.046	0.043	0.285
IGF-I	rs1832007	10	5254847	AKR1C4	A	G	-0.057	0.003	-0.031	0.044	0.484	0.032	0.042	0.448	0.019	0.046	0.674	0.128	0.056	0.023
IGF-I	rs2801482	10	12459773	CAMK1D	A	G	-0.05	0.008	0.167	0.099	0.093	0.147	0.090	0.102	0.141	0.103	0.171	-0.120	0.123	0.331
IGF-I	rs7921105	10	13535398	BEND7	T	C	-0.016	0.003	0.037	0.032	0.241	-0.051	0.030	0.093	0.023	0.033	0.481	-0.038	0.041	0.354
IGF-I	rs11012712	10	21760015	—	C	T	0.022	0.003	-0.067	0.040	0.091	-0.080	0.038	0.036	-0.044	0.042	0.293	0.062	0.052	0.238
IGF-I	rs10047326	10	22839463	PIP4K2A	A	C	0.017	0.003	-0.002	0.032	0.955	0.037	0.031	0.231	0.071	0.034	0.035	0.041	0.042	0.331
IGF-I	rs293275	10	53215020	PRKG1	T	C	-0.014	0.003	-0.019	0.031	0.548	-0.007	0.030	0.819	0.020	0.033	0.538	-0.013	0.041	0.747
IGF-I	rs10821713	10	62055781	ANK3	C	T	-0.017	0.003	0.003	0.032	0.926	-0.005	0.030	0.868	-0.027	0.033	0.414	-0.027	0.041	0.510
IGF-I	rs7910087	10	77209145	LRMDA	C	T	-0.017	0.003	0.045	0.032	0.156	0.018	0.030	0.554	0.016	0.034	0.638	0.009	0.041	0.821
IGF-I	rs4418728	10	94839724	CYP26A1	G	T	0.024	0.003	-0.020	0.032	0.518	0.009	0.030	0.766	0.077	0.033	0.020	0.005	0.040	0.893
IGF-I	rs116454156	10	95347041	FFAR4	A	G	0.078	0.01	0.095	0.175	0.588	-0.086	0.180	0.634	0.252	0.179	0.159	0.093	0.170	0.583
IGF-I	rs11187838	10	96038686	PLCE1	G	A	0.024	0.003	0.007	0.031	0.827	-0.034	0.030	0.257	0.047	0.033	0.160	0.064	0.041	0.117
IGF-I	rs10509746	10	102656897	PAX2	C	T	0.027	0.003	-0.008	0.031	0.800	0.059	0.030	0.049	-0.005	0.033	0.886	-0.030	0.041	0.478
IGF-I	rs4917962	10	103931931	NOLC1	G	T	-0.024	0.004	-0.006	0.048	0.908	0.064	0.045	0.159	0.046	0.049	0.350	-0.076	0.061	0.215
IGF-I	rs12244851	10	114773926	TCF7L2	C	T	-0.015	0.003	-0.093	0.034	0.006	-0.021	0.032	0.502	-0.044	0.035	0.209	-0.040	0.044	0.366
IGF-I	rs3858325	10	117988795	GFRA1	C	T	-0.019	0.003	0.013	0.031	0.688	-0.004	0.030	0.897	0.032	0.033	0.331	-0.033	0.041	0.414
IGF-I	rs61867536	11	1513700	MOB2	C	T	0.018	0.003	-0.046	0.032	0.147	-0.006	0.030	0.846	0.021	0.033	0.521	-0.078	0.041	0.056
IGF-I	rs3213223	11	2156930	IGF2/INS—IGF—2	G	A	-0.076	0.003	-0.044	0.039	0.255	-0.022	0.037	0.552	-0.014	0.040	0.737	0.008	0.049	0.871
IGF-I	rs11029620	11	3771924	NUP98	C	T	0.022	0.003	-0.019	0.038	0.609	0.044	0.036	0.221	0.028	0.040	0.477	-0.022	0.048	0.648
IGF-I	rs67257872	11	8530218	STK33	A	G	0.014	0.003	-0.031	0.031	0.317	0.011	0.030	0.716	-0.022	0.033	0.510	0.095	0.040	0.019
IGF-I	rs7947951	11	13356030	ARNTL	G	A	0.02	0.003	-0.024	0.033	0.472	0.075	0.032	0.017	-0.033	0.035	0.347	-0.002	0.043	0.956
IGF-I	rs72858776	11	15772953	AC087379.1	G	T	0.03	0.005	0.084	0.059	0.150	0.037	0.055	0.499	0.029	0.061	0.634	-0.109	0.077	0.157
IGF-I	rs11024614	11	18326758	HPS5	T	C	-0.023	0.003	-0.031	0.032	0.334	-0.022	0.030	0.479	-0.007	0.033	0.834	0.008	0.041	0.854
IGF-I	rs34452566	11	27793470	AC103796.1	G	T	-0.018	0.003	0.028	0.043	0.516	-0.021	0.041	0.603	-0.040	0.045	0.375	0.003	0.051	0.957
IGF-I	rs11031058	11	30375889	ARL14EP	C	T	-0.022	0.003	-0.015	0.042	0.711	-0.051	0.040	0.202	-0.030	0.044	0.492	0.066	0.054	0.220
IGF-I	rs117529631	11	46159633	AC024475.4	C	T	-0.041	0.006	-0.084	0.078	0.285	-0.057	0.077	0.457	0.080	0.084	0.340	-0.016	0.103	0.877
IGF-I	rs5896	11	46745003	F2	T	C	0.036	0.004	0.042	0.046	0.360	-0.009	0.044	0.841	0.112	0.049	0.022	0.051	0.060	0.394

IGF-I (continued)

							EXPOSURE (IGF)		CLL/SLL			DLBCL			FL			MM		
Instrument	rs	chr	bp	Gene	Effect Allele	Other Allele	Beta	SE	Beta[1]	SE	p-value	Beta[1]	SE	p-value	Beta[1]	SE	p-value	Beta[1]	SE	p-value
IGF-I	rs1051006	11	47306585	*MADD*	A	G	0.04	0.003	0.004	0.042	0.925	0.009	0.040	0.814	0.076	0.044	0.084	-0.010	0.058	0.865
IGF-I	rs1039481	11	48182237	*PTPRJ*	A	G	-0.042	0.003	0.028	0.037	0.446	-0.012	0.035	0.737	-0.071	0.038	0.063	-0.049	0.064	0.446
IGF-I	rs202676	11	49227620	*FOLH1*	G	A	0.021	0.003	-0.083	0.037	0.026	-0.028	0.036	0.438	0.092	0.039	0.017	0.034	0.070	0.627
IGF-I	rs10769621	11	49860463	*TRIM51FP*	T	C	0.02	0.003	-0.021	0.034	0.539	-0.030	0.033	0.357	0.087	0.036	0.014	-0.075	0.072	0.292
IGF-I	rs11230983	11	55541284	*OR5D13*	A	G	0.035	0.004	-0.088	0.048	0.065	-0.032	0.047	0.493	0.081	0.050	0.105	0.080	0.086	0.356
IGF-I	rs78460947	11	56143715	*OR8U1*	G	A	0.042	0.006	-0.197	0.190	0.301	-0.076	0.183	0.679	0.450	0.204	0.028	0.058	0.120	0.630
IGF-I	rs146345029	11	59596007	*GIF*	G	A	-0.034	0.006	0.201	0.078	0.010	0.208	0.078	0.007	0.003	0.087	0.972	-0.077	0.106	0.464
IGF-I	rs174554	11	61579463	*FADS1/FADS2*	A	G	0.022	0.003	-0.021	0.033	0.519	-0.025	0.032	0.428	-0.044	0.035	0.208	0.020	0.043	0.636
IGF-I	rs117104648	11	65543736	*AP5B1*	T	C	-0.036	0.005	0.003	0.070	0.970	-0.047	0.068	0.491	0.077	0.074	0.300	0.049	0.086	0.571
IGF-I	rs12790261	11	66988048	*KDM2A*	C	A	-0.031	0.005	0.112	0.094	0.234	0.154	0.090	0.086	-0.057	0.097	0.561	0.005	0.082	0.949
IGF-I	rs4980661	11	69306579	*CCND1*	A	G	0.014	0.003	-0.004	0.031	0.890	0.012	0.030	0.679	0.081	0.033	0.015	0.036	0.041	0.370
IGF-I	rs2512525	11	77923019	*USP35*	T	C	0.024	0.003	-0.049	0.042	0.251	0.059	0.040	0.136	0.024	0.044	0.584	0.013	0.054	0.814
IGF-I	rs61904289	11	85994731	*AP003084.1*	C	T	-0.016	0.003	-0.019	0.034	0.575	-0.054	0.032	0.094	0.007	0.035	0.843	0.040	0.044	0.370
IGF-I	rs625245	11	94192103	*MREII*	T	G	-0.016	0.003	0.026	0.033	0.435	0.036	0.032	0.261	0.082	0.035	0.018	-0.063	0.043	0.139
IGF-I	rs35023999	11	113266411	*ANKK1*	C	A	0.015	0.003	-0.022	0.031	0.473	-0.034	0.030	0.258	-0.014	0.033	0.666	-0.017	0.041	0.677
IGF-I	rs10892564	11	120224650	*ARHGEF12*	A	G	-0.017	0.003	0.026	0.032	0.419	0.049	0.031	0.110	0.006	0.034	0.869	-0.047	0.042	0.262
IGF-I	rs4936759	11	122763516	*C11orf63*	C	T	0.016	0.003	-0.011	0.032	0.725	-0.016	0.030	0.606	-0.051	0.033	0.129	0.007	0.041	0.865
IGF-I	rs10893499	11	126241979	*ST3GAL4*	G	A	0.022	0.004	-0.008	0.045	0.862	-0.033	0.043	0.441	-0.081	0.048	0.091	0.010	0.060	0.869
IGF-I	rs11064536	12	905582	*WNK1*	T	C	0.02	0.003	-0.024	0.042	0.568	0.023	0.040	0.567	-0.001	0.044	0.982	-0.063	0.054	0.247
IGF-I	rs2856321	12	11855773	*ETV6*	A	G	-0.026	0.003	-0.019	0.033	0.557	0.043	0.031	0.174	0.005	0.035	0.883	0.000	0.043	0.997
IGF-I	rs10841649	12	20954879	*SLCO1B3*	C	T	0.021	0.004	0.043	0.051	0.401	-0.074	0.049	0.127	-0.013	0.054	0.812	-0.079	0.059	0.184
IGF-I	rs9738365	12	31997635		C	A	-0.058	0.003	-0.038	0.036	0.284	-0.044	0.034	0.197	0.002	0.037	0.962	-0.006	0.047	0.904
IGF-I	rs12231073	12	38526901		G	T	-0.017	0.003	-0.003	0.036	0.933	-0.029	0.030	0.333	-0.034	0.033	0.291	0.130	0.080	0.106
IGF-I	rs11175935	12	40693806	*LRRK2*	G	T	0.02	0.003	0.036	0.040	0.373	-0.034	0.038	0.376	-0.021	0.042	0.615	0.039	0.053	0.460
IGF-I	rs247917	12	46265916	*ARID2*	C	T	-0.015	0.003	-0.005	0.031	0.868	0.000	0.030	0.999	-0.071	0.033	0.031	-0.019	0.041	0.647
IGF-I	rs117564283	12	52300110	*ACVRL1*	C	T	-0.029	0.005	0.117	0.064	0.067	0.062	0.061	0.314	0.001	0.067	0.992	-0.010	0.078	0.900
IGF-I	rs773116	12	56486159	*ERBB3*	G	A	0.016	0.003	-0.058	0.032	0.070	-0.023	0.031	0.458	0.014	0.033	0.668	-0.010	0.040	0.797
IGF-I	rs78607331	12	57648644	*R3HDM2*	T	C	-0.037	0.006	-0.062	0.079	0.429	-0.026	0.076	0.730	-0.021	0.084	0.799	-0.128	0.108	0.232
IGF-I	rs4547160	12	63503650	*AVPR1A*	G	T	-0.018	0.003	0.023	0.033	0.483	0.055	0.032	0.081	0.020	0.035	0.566	-0.029	0.043	0.509
IGF-I	rs1351394	12	66351826	*HMGA2*	C	T	0.024	0.003	-0.036	0.031	0.252	-0.023	0.030	0.441	0.031	0.033	0.347	0.015	0.041	0.720
IGF-I	rs2230281	12	89917518	*GALN/T4/POC1B —GALNT4*	G	A	-0.016	0.003	-0.014	0.035	0.683	0.010	0.033	0.759	0.012	0.037	0.737	-0.006	0.045	0.886
IGF-I	rs10777540	12	94150321	*CRADD*	T	G	-0.018	0.003	-0.026	0.031	0.403	0.009	0.030	0.767	-0.028	0.033	0.392	0.047	0.041	0.245
IGF-I	rs10860237	12	98157010	*AC007424.1*	G	A	-0.03	0.003	0.014	0.034	0.682	0.011	0.032	0.734	0.034	0.035	0.341	-0.063	0.043	0.141
IGF-I	rs11111274	12	102838128	*IGF1*	A	G	-0.08	0.003	-0.004	0.035	0.908	-0.013	0.034	0.708	-0.027	0.037	0.457	-0.032	0.046	0.473
IGF-I	rs10745954	12	103483094	*AC068643.1*	G	A	0.015	0.003	-0.002	0.031	0.959	-0.060	0.030	0.047	0.001	0.033	0.970	-0.027	0.041	0.503
IGF-I	rs7314285	12	111522026	*CUX2*	T	G	-0.052	0.005	-0.063	0.062	0.306	-0.032	0.058	0.581	-0.039	0.063	0.538	-0.030	0.076	0.692
IGF-I	rs1061657	12	115108136	*TBX3*	T	C	-0.022	0.003	0.047	0.037	0.205	0.061	0.035	0.084	-0.013	0.039	0.740	-0.080	0.046	0.081
IGF-I	rs2460488	12	116187660		G	A	0.026	0.003	-0.089	0.044	0.045	-0.018	0.042	0.670	-0.029	0.046	0.520	-0.027	0.054	0.612
IGF-I	rs1800574	12	121416864	*HNF1A*	T	C	0.145	0.007	0.102	0.101	0.309	0.066	0.097	0.499	0.000	0.106	0.998	0.075	0.127	0.555
IGF-I	rs11057265	12	123805950	*SBNO1*	G	A	0.044	0.007	-0.122	0.090	0.172	0.082	0.086	0.344	0.125	0.093	0.179	0.058	0.117	0.617
IGF-I	rs9532512	13	40769897	*LINC00598*	G	A	-0.043	0.003	-0.019	0.040	0.639	0.007	0.038	0.849	-0.020	0.042	0.629	-0.016	0.052	0.760
IGF-I	rs1170158	13	42701941	*DGKH*	T	G	0.021	0.003	-0.024	0.040	0.542	0.007	0.038	0.846	-0.022	0.042	0.604	0.012	0.051	0.817
IGF-I	rs1535793	13	47154966	*LRCH1*	A	G	0.024	0.003	-0.005	0.036	0.899	0.063	0.035	0.070	0.087	0.038	0.021	0.020	0.047	0.676
IGF-I	rs118081390	13	49671053	*FNDC3A*	G	A	0.028	0.005	0.008	0.059	0.888	0.064	0.056	0.254	0.041	0.062	0.501	0.099	0.079	0.211
IGF-I	rs9573360	13	74771429	*KLF12*	A	C	0.014	0.003	-0.024	0.031	0.451	-0.017	0.030	0.580	-0.026	0.033	0.425	-0.023	0.041	0.573
IGF-I	rs71432868	13	106559402	*SNORA25*	T	C	-0.028	0.005	0.007	0.093	0.938	-0.034	0.090	0.704	0.216	0.096	0.025		NA	
IGF-I	rs9583151	13	107666257		C	T	0.014	0.003	0.002	0.031	0.957	0.021	0.030	0.472	-0.037	0.033	0.259	0.032	0.041	0.433
IGF-I	rs7323205	13	110365525	*LINC00676*	C	T	0.015	0.003	-0.017	0.032	0.597	0.065	0.031	0.035	0.100	0.034	0.004	-0.019	0.042	0.646
IGF-I	rs6602909	13	114551993	*GAS6*	T	C	-0.02	0.003	-0.019	0.033	0.564	-0.061	0.032	0.054	-0.009	0.035	0.789	0.033	0.043	0.439
IGF-I	rs8017377	14	24883887	*NYNRIN*	A	G	-0.017	0.003	-0.009	0.032	0.788	0.001	0.030	0.967	-0.052	0.033	0.117	0.006	0.041	0.884
IGF-I	rs28396553	14	36673392	*lincRNA*	T	C	0.015	0.003	0.003	0.032	0.926	0.002	0.031	0.953	-0.030	0.034	0.376	0.037	0.041	0.371
IGF-I	rs33912345	14	60976537	*SIX6*	A	C	-0.023	0.003	0.031	0.032	0.331	0.017	0.031	0.586	0.014	0.033	0.666	0.007	0.042	0.876
IGF-I	rs79936318	14	64315556	*SYNE2*	G	A	-0.017	0.003	0.031	0.047	0.503	0.036	0.044	0.421	0.038	0.049	0.440	0.070	0.053	0.185
IGF-I	rs168961	14	69282930	*ZFP36L1*	A	G	-0.018	0.003	0.001	0.032	0.967	-0.018	0.030	0.540	-0.033	0.033	0.326	-0.054	0.041	0.186
IGF-I	rs75088740	14	69819101	*GALNT16*	G	A	-0.019	0.003	-0.027	0.042	0.532	0.081	0.040	0.042	0.094	0.045	0.036	0.040	0.053	0.451
IGF-I	rs13379043	14	74250126	*ELMSAN1*	T	C	0.025	0.003	0.037	0.036	0.299	0.033	0.034	0.325	0.013	0.038	0.731	-0.031	0.045	0.497
IGF-I	rs175043	14	75471803	*EIF2B2*	G	A	0.018	0.003	-0.015	0.031	0.630	-0.008	0.030	0.795	-0.032	0.033	0.324	0.009	0.041	0.826
IGF-I	rs1061638	14	77928525	*AHSA1*	G	A	0.018	0.003	-0.004	0.034	0.898	-0.040	0.032	0.218	0.023	0.035	0.515	0.096	0.044	0.030
IGF-I	rs10145154	14	79939525	*NRXN3*	C	T	-0.018	0.003	-0.042	0.038	0.270	-0.043	0.037	0.237	-0.015	0.040	0.707	-0.052	0.049	0.289
IGF-I	rs78598185	14	92791479	*SLC24A4*	A	G	-0.029	0.004	0.060	0.056	0.284	0.027	0.052	0.606	-0.025	0.059	0.670	-0.016	0.070	0.813
IGF-I	rs1115897	14	93910816	*UNC79*	A	C	0.021	0.003	0.005	0.033	0.869	0.012	0.032	0.720	0.028	0.035	0.418	-0.014	0.044	0.748
IGF-I	rs28929474	14	94844947	*SERPINA1*	T	C	-0.063	0.009	0.058	0.115	0.612	-0.178	0.116	0.124	-0.054	0.125	0.668	-0.072	0.155	0.641
IGF-I	rs10136874	14	101202022	*DLK1*	G	T	0.023	0.003	0.017	0.032	0.591	-0.015	0.030	0.622	0.043	0.033	0.198	0.083	0.041	0.044
IGF-I	rs17747633	15	40916237	*KNL1*	G	A	0.015	0.003	0.049	0.032	0.123	-0.027	0.030	0.382	-0.046	0.033	0.165	0.007	0.042	0.859
IGF-I	rs62020698	15	43237414	*UBR1*	C	T	0.047	0.004	0.025	0.068	0.716	0.033	0.064	0.607	-0.026	0.073	0.722	0.161	0.075	0.031
IGF-I	rs55707100	15	43820717	*MAP1A*	T	C	-0.151	0.008	0.022	0.088	0.801	0.039	0.081	0.629	0.149	0.090	0.098	-0.171	0.123	0.163
IGF-I	rs4273010	15	44947434	*SPG11*	T	C	0.128	0.008	-0.225	0.123	0.068	-0.109	0.112	0.332	0.048	0.130	0.711	0.141	0.139	0.308
IGF-I	rs4545755	15	51549044	*MIR4713HG/CYP/19A1*	G	A	0.016	0.003	0.052	0.032	0.099	-0.015	0.030	0.622	-0.065	0.033	0.052	-0.023	0.041	0.585
IGF-I	rs12442867	15	62489128	*AC126323.2*	C	A	-0.017	0.003	-0.025	0.032	0.424	0.028	0.031	0.367	0.037	0.034	0.265	-0.005	0.041	0.902
IGF-I	rs79076440	15	63803863	*USP3*	A	G	0.019	0.003	0.014	0.041	0.740	-0.007	0.039	0.856	-0.031	0.043	0.470	-0.001	0.054	0.984

IGF-I (continued)

							EXPOSURE (IGF)		CLL/SLL			DLBCL			FL			MM		
Instrument	rs	chr	bp	Gene	Effect Allele	Other Allele	Beta	SE	Beta[1]	SE	p-value	Beta[1]	SE	p-value	Beta[1]	SE	p-value	Beta[1]	SE	p-value
**IGF-I**	rs10851736	15	64940718	*ZNF609*	C	T	-0.027	0.005	-0.013	0.055	0.811	0.004	0.053	0.944	-0.068	0.057	0.238	0.044	0.069	0.518
**IGF-I**	rs8024330	15	67443926	*SMAD3*	C	T	0.018	0.003	0.037	0.034	0.270	0.014	0.033	0.670	-0.053	0.036	0.143	0.060	0.044	0.172
**IGF-I**	rs8033075	15	68353652	*PIAS1*	A	G	0.045	0.005	-0.085	0.071	0.227	-0.071	0.068	0.294	0.013	0.073	0.854	0.019	0.093	0.844
**IGF-I**	rs5742915	15	74336633	*PML*	C	T	0.025	0.003	-0.030	0.031	0.332	0.025	0.030	0.409	0.001	0.033	0.986	0.092	0.041	0.024
**IGF-I**	rs12593755	15	89111712	*AC013489.2*	G	T	-0.016	0.003	-0.065	0.032	0.043	-0.033	0.031	0.280	-0.025	0.034	0.459	-0.040	0.042	0.342
**IGF-I**	rs11856160	15	93452846	*CHD2*	A	G	0.021	0.003	-0.029	0.043	0.492	-0.076	0.041	0.066	-0.017	0.045	0.713	-0.026	0.055	0.647
**IGF-I**	rs12912439	15	95828705	*LINC01197*	C	T	-0.022	0.003	0.093	0.035	0.008	0.017	0.033	0.622	0.003	0.037	0.934	-0.020	0.045	0.657
**IGF-I**	rs34040697	15	97125666		A	G	0.016	0.003	0.037	0.033	0.255	0.032	0.031	0.314	0.006	0.035	0.852	0.005	0.041	0.896
**IGF-I**	rs142354201	15	99524022	*PGPEP1L*	G	A	0.034	0.006	-0.144	0.100	0.152	0.002	0.093	0.979	-0.181	0.104	0.082	-0.079	0.098	0.421
**IGF-I**	rs4988483	16	1129010	*SSTRS*	A	C	-0.172	0.006	-0.275	0.103	0.007	-0.015	0.098	0.882	-0.118	0.106	0.266		NA	
**IGF-I**	rs72761177	16	1833508	*NUBP2*	A	G	0.077	0.004	-0.113	0.059	0.055	-0.008	0.056	0.886	0.043	0.061	0.477	0.061	0.075	0.413
**IGF-I**	rs11077337	16	3492048	*AC0252832/ZNF597 CORO7*	T	G	0.015	0.003	0.009	0.032	0.780	-0.015	0.030	0.626	-0.008	0.033	0.820	-0.006	0.041	0.878
**IGF-I**	rs8182173	16	4420787	*CORO7—PAM16*	C	T	-0.018	0.003	0.025	0.037	0.495	0.042	0.036	0.240	0.044	0.039	0.253	0.014	0.047	0.760
**IGF-I**	rs74774288	16	5922263	*RBFOX1*	G	T	0.027	0.003	-0.084	0.041	0.042	-0.018	0.040	0.649	-0.008	0.044	0.849	-0.122	0.054	0.023
**IGF-I**	rs4985062	16	8996636	*USP7*	T	C	0.015	0.003	0.020	0.032	0.526	-0.072	0.031	0.020	-0.028	0.034	0.404	0.019	0.041	0.651
**IGF-I**	rs1532824	16	10532211	*ATF7IP2*	C	A	-0.017	0.003	-0.051	0.035	0.144	-0.023	0.033	0.491	-0.044	0.036	0.224	0.048	0.046	0.296
**IGF-I**	rs12935465	16	17476853	*XYLT1*	T	C	0.016	0.003	0.017	0.031	0.589	-0.058	0.030	0.054	-0.024	0.033	0.464	0.038	0.041	0.340
**IGF-I**	rs2023762	16	19276597	*SYT17*	T	C	0.015	0.003	0.041	0.031	0.196	0.013	0.030	0.663	-0.002	0.033	0.954	-0.072	0.040	0.073
**IGF-I**	rs12927172	16	27325021	*IL4R*	G	A	-0.015	0.003	-0.006	0.032	0.845	0.009	0.031	0.783	0.043	0.034	0.216	-0.019	0.041	0.644
**IGF-I**	rs7498665	16	28883241	*SH2B1*	G	A	0.019	0.003	0.044	0.032	0.164	0.036	0.031	0.241	0.026	0.034	0.436	-0.006	0.041	0.885
**IGF-I**	rs4788220	16	30063780	*FAM57B*	A	G	-0.017	0.003	0.058	0.032	0.070	0.026	0.030	0.401	0.057	0.033	0.087	-0.039	0.041	0.344
**IGF-I**	rs750952	16	31093954	*ZNF646*	C	T	0.032	0.003	-0.017	0.032	0.600	-0.038	0.031	0.215	-0.078	0.034	0.022	-0.069	0.041	0.096
**IGF-I**	rs116971887	16	51170026	*SALL1*	G	T	0.036	0.006	0.063	0.088	0.475	-0.008	0.083	0.922	0.038	0.093	0.681	0.006	0.098	0.951
**IGF-I**	rs12597502	16	53170069	*CHD9*	A	G	-0.015	0.003	-0.023	0.035	0.502	-0.039	0.033	0.240	-0.057	0.036	0.118	0.080	0.046	0.082
**IGF-I**	rs1548917	16	56109333	*CESSA*	C	T	-0.015	0.003	-0.014	0.032	0.650	0.007	0.030	0.825	-0.066	0.033	0.046	0.012	0.041	0.765
**IGF-I**	rs111792934	16	69131293	*HAS3*	C	T	0.022	0.003	0.019	0.043	0.655	0.012	0.041	0.774	-0.028	0.044	0.530	0.044	0.056	0.434
**IGF-I**	rs17299478	16	69775500		C	T	0.032	0.003	0.017	0.044	0.703	0.054	0.043	0.205	-0.046	0.047	0.331	-0.018	0.057	0.758
**IGF-I**	rs12935091	16	71525208	*ZNF19*	A	G	-0.035	0.006	-0.056	0.081	0.491	-0.002	0.078	0.983	0.050	0.086	0.558	0.148	0.102	0.147
**IGF-I**	rs147491123	16	72567795	*LINC01572*	C	T	0.036	0.007	-0.095	0.090	0.294	0.065	0.084	0.442	0.046	0.093	0.623	0.031	0.111	0.785
**IGF-I**	rs8059803	16	81603001	*CMIP*	A	G	0.031	0.003	0.019	0.035	0.583	-0.022	0.033	0.515	0.055	0.037	0.131	-0.034	0.044	0.428
**IGF-I**	rs11149612	16	83980965	*AC009119.2*	C	T	0.027	0.003	0.015	0.032	0.644	-0.017	0.031	0.582	-0.038	0.034	0.269	-0.028	0.041	0.483
**IGF-I**	rs8054322	16	85201405	*GSE1*	G	A	-0.015	0.003	-0.015	0.031	0.628	-0.028	0.030	0.354	0.005	0.033	0.874	-0.029	0.041	0.470
**IGF-I**	rs7502910	17	1638718	*WDR81*	A	G	0.016	0.003	-0.043	0.032	0.174	-0.023	0.030	0.441	-0.073	0.033	0.029	0.019	0.041	0.637
**IGF-I**	rs2309401	17	5471902	*NLRP1*	T	G	0.015	0.003	0.022	0.032	0.490	0.037	0.030	0.213	-0.003	0.033	0.919	-0.066	0.041	0.105
**IGF-I**	rs9892862	17	7439014	*POLR2A*	G	A	0.022	0.003	-0.078	0.038	0.040	-0.029	0.037	0.436	-0.007	0.040	0.863	-0.037	0.049	0.443
**IGF-I**	rs6416868	17	15924370	*TTC19*	G	A	-0.019	0.003	0.015	0.031	0.640	-0.038	0.030	0.200	0.042	0.033	0.208	-0.005	0.041	0.902
**IGF-I**	rs8075153	17	17622666	*RAI1*	C	T	0.021	0.003	-0.078	0.032	0.014	0.048	0.030	0.113	0.022	0.033	0.505	-0.014	0.041	0.741
**IGF-I**	rs8079923	17	19869544	*AKAP10*	C	T	0.016	0.003	0.000	0.036	0.989	-0.010	0.035	0.773	0.005	0.038	0.886	0.022	0.047	0.646
**IGF-I**	rs56030650	17	38131187	*GSDMA*	A	C	-0.022	0.003	-0.014	0.031	0.648	0.001	0.030	0.979	-0.015	0.033	0.655	0.107	0.040	0.008
**IGF-I**	rs668799	17	40716235	*COASY*	C	T	0.018	0.003	0.027	0.036	0.450	0.011	0.034	0.759	-0.047	0.038	0.218	0.054	0.046	0.248
**IGF-I**	rs199525	17	44847834	*WNT3*	T	G	-0.02	0.003	-0.067	0.040	0.090	-0.041	0.037	0.273	-0.053	0.042	0.203	-0.026	0.050	0.598
**IGF-I**	rs11079157	17	53360799	*HLF*	G	T	-0.02	0.003	-0.009	0.037	0.811	-0.001	0.036	0.983	0.028	0.039	0.480	-0.083	0.048	0.086
**IGF-I**	rs2250014	17	57836134	*VMP1*	T	C	-0.021	0.003	0.038	0.042	0.367	0.012	0.040	0.759	0.018	0.045	0.686	0.004	0.054	0.944
**IGF-I**	rs142377191	17	61649170	*DCAF7*	G	A	-0.125	0.009	-0.028	0.134	0.835	-0.045	0.127	0.724	-0.151	0.145	0.296		NA	
**IGF-I**	rs76708468	17	62206299	*ERN1*	T	C	-0.087	0.006	-0.104	0.113	0.358	-0.179	0.109	0.102	-0.022	0.119	0.856		NA	
**IGF-I**	rs78357146	17	64305051	*PRKCA*	A	G	-0.09	0.007	0.186	0.116	0.107	0.075	0.110	0.497	0.062	0.118	0.601		NA	
**IGF-I**	rs77542162	17	67081278	*ABCA6*	G	A	0.054	0.009	-0.162	0.108	0.134	-0.027	0.104	0.799	-0.183	0.111	0.101	0.057	0.155	0.711
**IGF-I**	rs6501601	17	71124903	*SLC39A11*	G	A	0.015	0.003	0.023	0.034	0.499	-0.011	0.032	0.722	0.000	0.035	0.997	0.070	0.044	0.114
**IGF-I**	rs4789227	17	73794354	*UNK*	T	C	0.015	0.003	0.004	0.033	0.898	0.027	0.032	0.400	0.051	0.035	0.141	0.029	0.043	0.495
**IGF-I**	rs4075483	17	79074817	*BAIAP2*	C	T	0.017	0.003	-0.007	0.033	0.839	0.015	0.031	0.622	-0.007	0.034	0.844	0.009	0.042	0.822
**IGF-I**	rs8095538	18	1616505		T	G	-0.02	0.003	0.018	0.034	0.601	0.031	0.032	0.331	0.041	0.036	0.255	0.016	0.045	0.719
**IGF-I**	rs8084351	18	50726559	*DCC*	G	A	0.015	0.003	0.034	0.031	0.277	0.002	0.030	0.949	0.035	0.033	0.287	-0.005	0.040	0.899
**IGF-I**	rs11152071	18	56087417	*AC105105.3*	C	T	0.02	0.003	-0.048	0.036	0.191	-0.002	0.035	0.966	-0.016	0.038	0.671	0.006	0.047	0.894
**IGF-I**	rs190102446	18	57048571	—	C	T	0.041	0.007	-0.065	0.084	0.434	0.111	0.080	0.163	0.122	0.087	0.164	0.194	0.108	0.074
**IGF-I**	rs585187	18	58177124	*MRPS5P4*	T	G	0.015	0.003	-0.002	0.031	0.949	-0.032	0.030	0.279	0.017	0.033	0.599	0.017	0.040	0.675
**IGF-I**	rs12454712	18	60845884	*BCL2*	T	C	0.018	0.003	0.027	0.033	0.421	-0.002	0.031	0.939	0.020	0.035	0.572	-0.032	0.042	0.449
**IGF-I**	rs8097893	18	74983055	*GALR1*	A	G	0.058	0.006	-0.106	0.075	0.159	-0.123	0.074	0.096	-0.022	0.079	0.783	0.023	0.092	0.806
**IGF-I**	rs67868323	19	4048561	*ZBTB7A*	T	G	0.016	0.003	-0.133	0.036	0.000	-0.009	0.034	0.803	0.052	0.038	0.173	0.031	0.046	0.499
**IGF-I**	rs2602717	19	4902950	*UHRF1/ARRDCS*	C	T	0.019	0.003	-0.116	0.047	0.013	0.008	0.045	0.863	-0.032	0.049	0.522	-0.034	0.049	0.486
**IGF-I**	rs8112883	19	7179320	*INSR*	G	T	0.017	0.003	0.011	0.034	0.741	0.012	0.033	0.707	-0.013	0.036	0.714	-0.047	0.044	0.287
**IGF-I**	rs8105174	19	10347032	*DNMT1*	C	T	0.05	0.003	0.089	0.041	0.032	0.109	0.039	0.005	0.038	0.044	0.386	0.042	0.053	0.427
**IGF-I**	rs6510033	19	30710785	*AC005597.1*	A	G	0.02	0.003	0.022	0.035	0.539	-0.022	0.034	0.516	-0.018	0.037	0.623	0.062	0.046	0.180
**IGF-I**	rs6510177	19	31211647	*ZNF536*	C	T	-0.023	0.003	-0.127	0.044	0.004	-0.043	0.042	0.315	-0.096	0.047	0.040	-0.117	0.054	0.031
**IGF-I**	rs62102136	19	34700561	*LSM14A*	C	T	0.016	0.003	-0.007	0.034	0.835	0.022	0.033	0.497	0.038	0.036	0.283	0.077	0.044	0.083
**IGF-I**	rs7254601	19	36147315	*COX6B1*	A	G	-0.016	0.003	-0.029	0.036	0.426	-0.030	0.035	0.391	-0.002	0.038	0.951	-0.019	0.048	0.690
**IGF-I**	rs58560372	19	38758752	*SPINT2*	C	T	0.02	0.003	-0.015	0.045	0.731	-0.008	0.043	0.861	-0.020	0.048	0.673	0.009	0.058	0.880
**IGF-I**	rs15052	19	41813375	*HNRN PUL1/ TGFB1 ZC3H4*	T	C	-0.018	0.003	-0.033	0.045	0.466	0.048	0.045	0.278	-0.020	0.049	0.679	-0.024	0.055	0.660
**IGF-I**	rs11671304	19	47564643		T	C	-0.018	0.003	-0.108	0.034	0.002	-0.047	0.033	0.153	-0.054	0.036	0.136	0.011	0.043	0.801
**IGF-I**	rs296361	19	48389363	*SULT2A1*	G	A	-0.025	0.003	-0.042	0.043	0.324	-0.009	0.041	0.823	-0.029	0.045	0.520	-0.016	0.056	0.774
**IGF-I**	rs2287922	19	49232226	*RASIP1*	A	G	-0.03	0.003	-0.012	0.031	0.707	-0.018	0.030	0.557	-0.081	0.033	0.015	0.018	0.041	0.662

IGF-I (continued)

Instrument	rs	chr	bp	Gene	Effect Allele	Other Allele	EXPOSURE (IGF) Beta	SE	CLL/SLL Beta[1]	SE	p-value	DLBCL Beta[1]	SE	p-value	FL Beta[1]	SE	p-value	MM Beta[1]	SE	p-value
**IGF-I**	rs7256521	19	53837110	*ZNF845*	A	G	-0.015	0.003	-0.028	0.033	0.397	-0.006	0.032	0.851	0.004	0.035	0.905	-0.041	0.041	0.308
**IGF-I**	rs12975366	19	54759361	*LILRBS*	C	T	-0.02	0.003	-0.002	0.033	0.947	0.027	0.032	0.398	0.036	0.035	0.303	0.005	0.041	0.897
**IGF-I**	rs6037508	20	3217989	*SLC4A11*	T	G	-0.017	0.003	0.044	0.039	0.265	0.050	0.037	0.175	0.035	0.040	0.386	0.036	0.050	0.467
**IGF-I**	rs7267595	20	10643850	*JAG1*	A	C	0.015	0.003	0.022	0.031	0.479	0.036	0.030	0.231	0.010	0.033	0.753	-0.051	0.041	0.206
**IGF-I**	rs2273058	20	20033319	*CRNKL1*	G	A	-0.022	0.003	-0.030	0.031	0.343	-0.011	0.030	0.707	-0.014	0.033	0.671	-0.039	0.041	0.331
**IGF-I**	rs6106324	20	20964988	*AL133465.1*	T	C	0.019	0.003	0.013	0.032	0.696	0.032	0.031	0.297	0.030	0.034	0.376	-0.083	0.042	0.050
**IGF-I**	rs2424396	20	21630280	*LINC01726*	A	G	-0.033	0.004	-0.005	0.054	0.919	-0.035	0.052	0.499	-0.145	0.058	0.012	-0.091	0.069	0.191
**IGF-I**	rs6088579	20	33284624	*PIGU/NCOA6*	G	A	0.027	0.003	0.043	0.043	0.320	0.065	0.041	0.117	-0.014	0.045	0.764	-0.001	0.057	0.991
**IGF-I**	rs2207132	20	39142516	*MAFB*	G	A	0.048	0.007	0.127	0.093	0.173	-0.039	0.092	0.671	-0.024	0.101	0.812	0.108	0.111	0.329
**IGF-I**	rs17265513	20	39832628	*ZHX3*	C	T	-0.022	0.003	-0.027	0.040	0.493	0.040	0.038	0.302	0.056	0.042	0.180	-0.031	0.052	0.563
**IGF-I**	rs16995311	20	49201102	*PTPN1*	A	C	0.04	0.005	-0.079	0.058	0.176	-0.153	0.056	0.007	-0.168	0.062	0.007	-0.113	0.079	0.153
**IGF-I**	rs2104476	20	54852856		A	G	-0.02	0.003	-0.077	0.035	0.028	-0.041	0.034	0.226	-0.042	0.037	0.256	-0.069	0.046	0.132
**IGF-I**	rs2738787	20	62328375	*RTEL*	G	A	-0.037	0.005	-0.138	0.062	0.025	-0.122	0.056	0.030	-0.007	0.064	0.917	-0.065	0.076	0.393
**IGF-I**	rs9978775	21	40694526	*BRWD1—AS1*	G	A	0.019	0.003	0.030	0.031	0.345	0.037	0.030	0.218	0.056	0.033	0.095	0.023	0.042	0.587
**IGF-I**	rs8138950	22	29448643	*ZNRF3*	C	T	0.015	0.003	0.011	0.032	0.728	0.037	0.030	0.219	0.013	0.033	0.689	0.001	0.040	0.976
**IGF-I**	rs2412973	22	30529631	*HORMAD2*	C	A	-0.014	0.003	-0.022	0.032	0.499	-0.030	0.031	0.321	-0.036	0.034	0.285	-0.019	0.041	0.650
**IGF-I**	rs12106594	22	31885316	*DRG1/*	C	T	-0.036	0.006	-0.103	0.070	0.143	-0.014	0.066	0.837	-0.011	0.074	0.884	-0.048	0.091	0.599
**IGF-I**	rs5755948	22	36179095	*RBFOX2*	G	A	-0.028	0.004	-0.032	0.046	0.485	-0.039	0.044	0.371	-0.031	0.048	0.521	-0.090	0.058	0.118
**IGF-I**	rs6519133	22	39096602	*JOSD1*	T	C	0.029	0.003	-0.012	0.033	0.727	-0.037	0.032	0.247	-0.059	0.035	0.093	0.044	0.041	0.289
**IGF-I**	rs9611565	22	41767486	*TEF*	T	C	0.029	0.003	-0.045	0.036	0.217	-0.045	0.034	0.191	0.054	0.038	0.159	-0.039	0.046	0.397
**IGF-I**	rs4823324	22	46238123	*ATXN10*	T	C	0.016	0.003	0.032	0.032	0.314	0.055	0.030	0.072	0.023	0.034	0.500	-0.035	0.041	0.396

[1]: log-additive models. FL: follicular lymphoma, DLBCL: diffuse large B-cell lymphoma, CLL/SLL: chronic lymphocytic leukaemia/small lymphocytic leukaemia, MM: multiple myeloma, OR: odds ratio; CI: confidence interval; SE: Standard error.

SUPPLEMENTARY TABLE S4. List of INTERLYMPH studies, their funding and special acknowledgements.

Abbreviation (Study name)	Subtypes	References
ATBC (Alpha-Tocopherol, Beta-Carotene Cancer Prevention Study)	DLBCL, FL, CLL/SLL	PMID: 8205268
BC (British Columbia Non-Hodgkin Lymphoma Study)	MM	PMID: 30289833/ PMID: 32569378
	DLBCL, FL, CLL/SLL	PMID: 17722095
CPS-II (American Cancer Society Cancer Prevention Study-II Nutrition Cohort)	DLBCL, MM, FL, CLL/SLL	PMID: 12015775
Engela (Environmental and genetic risks factors study in adult lymphoma)	DLBCL, FL, CLL/SLL	PMID: 19058175
EPIC (European Prospective Investigation into Cancer, Chronic Diseases, Nutrition and Lifestyles)	DLBCL, MM, FL, CLL/SLL	PMID: 17443415
Epilymph (Epilymph case-control study in six European countries)	DLBCL, MM, FL, CLL/SLL	PMID: 17087949
GEC (Genetic Epidemiology of CLL)	CLL/SLL	PMID: 21131588
GELA (Groupe d'Etude des Lymphomes de l'Adulte)	DLBCL	PMID 29025017, PMID: 23578722, PMID: 21482186, PMID: 21546499, PMID: 23235801
HPFS (Health Professionals Follow-up Study)	DLBCL, FL, CLL/SLL	PMID: 1678444
UCSF/NHS	DLBCL, FL, CLL/SLL	PMID: 19620980 PMCID: PMC2823809
Iowa-Mayo SPORE	DLBCL, FL, CLL/SLL	PMID 29025017
IMAGE-UAB	MM	PMID: 38849476
Italy GxE (Multicenter Italian study on gene-environment interactions in lymphoma etiology: translational aspects)	DLBCL, FL, MM, CLL/SLL	PMID: 33910586
Mayo Clinic case-control study	DLBCL, FL, CLL/SLL	PMID: 21686124
Mayo MM/MGUS	MM	PMID: 32569378
MCCS (The Melbourne Collaborative Cohort Study)	DLBCL, MM, FL, CLL/SLL	PMID: 28641380
MD Anderson	MM	PMID: 32569378
Memorial-Sloan Kettering (MSKCC)	DLBCL, FL, CLL/SLL	PMID: 23349640
National Cancer Institute (SEER)	DLBCL, FL, SLL	PMID: 15342441
New South Wales (NSW)	DLBCL, FL, CLL/SLL	PMID: 15386383
NHS (Nurses' Health Study)	DLBCL, FL, CLL/SLL	PMID: 15864280/ PMID: 7658481
NYUWHS (New York University Women's Health Study)	CLL/SLL, DLBCL, FL	PMID: 1873553
PLCO (Prostate, Lung, Colorectal, and Ovarian Cancer Screening Trial)	CLL/SLL, DLBCL, FL	PMID: 37958403
PLCO MM (Prostate, Lung, Colorectal, and Ovarian Cancer Screening Trial)	MM	PMID: 32569378
SCALE (Scandinavian Lymphoma Etiology Study; Karolinska; Sweden)	CLL/SLL, FL, DLBCL	PMID: 15687363

SCALE (Scandinavian Lymphoma Etiology Study; Statens Serum, Denmark)	CLL/SLL, FL, DLBCL	PMID: 15687363
UCSF2, UCSF1 (Molecular Epidemiology of non-Hodgkin lymphoma)	DLBCL, FL, CLL/SLL	PMID: 19620980; PMCID: PMC2823809
UCSF MM (Molecular Epidemiology of non-Hodgkin lymphoma)	MM	PMID: 32569378
ELCCS (Epidemiology & Genetics Unit Lymphoma Case-Control study; University of York (UK)	DLBCL, FL	PMID: 15456990
USC MM	MM	PMID: 32569378
Utah/Usher (Utah-Sheffield Hematology Epidemiology Research)	MM	PMID: 32569378
WHI (Women's Health Initiative)	DLBCL, FL, CLL/SLL	PMID: 14575938
Yale University (Population-based case-control study in Connecticut women)	DLBCL, MM, FL, CLL/SLL	PMID: 16264183

FUNDING AND ACKNOWLEDGMENTS FOR INDIVIDUAL STUDY

For studies in the multiple myeloma group:

This work was supported in part by the National Institutes of Health, National Cancer Institute (U01CA257679, R25 CA092049, P30 CA016672, R01 CA134674, P30 CA042014, R01 CA300518, R01 CA249955, R01 CA186646, U54 CA118948, P30 CA13148, R01 CA235026, R01 CA107476, R01 CA168762, R01 CA087014, R01 CA122663, P50 CA186781, NCI SEER Program HHSN261201800016I, and the National Cancer Institute Intramural Research Program), the US Center for Disease Control and Prevention's National Program of Cancer Registries (NU58DP007131), the Leukemia Lymphoma Society (6067-09), the Utah Population Database, the Utah Cancer Registry, Huntsman Cancer Institute, the Canadian Institutes of Health Research (81274), VicHealth, Cancer Council Victoria, the Australian National Health and Medical Research Council (209057, 396414, and 1074383), the Victorian Cancer Registry, the Australian Institute of Health and Welfare, the Australian National Death Index, the Australian Cancer Database, and the Mayo Clinic Cancer Center. The authors thank all site investigators that contributed to the studies within the Multiple Myeloma Working Group (InterLymph Consortium), staff involved at each site (ascertainment, laboratory, biobanking, and research informatics teams), and most importantly, the study participants for their contributions that made this study possible.

For studies included in the NHL GWAS:

GEC/Mayo GWAS – National Institutes of Health (CA118444, CA148690, CA92153). Intramural Research Program of the NIH, National Cancer Institute. Veterans Affairs Research Service. Data collection for Duke University was supported by a Leukemia & Lymphoma Society Career Development Award, the Bernstein Family Fund for Leukemia and Lymphoma Research, and the National Institutes of Health (K08CA134919), National Center for Advancing Translational Science (UL1 TR000135)

Iowa-Mayo SPORE – National Institutes of Health (P50 CA97274, R01 CA129539, and R01 CA200703) and the Henry J. Predolin Foundation**.**

Myeloma SPORE - 2P50CA100707 Project 6 to WC; SEER contract to USC N01-PC-35139 (NHL samples).

Mayo Clinic Case-Control – National Institutes of Health (R01 CA92153)**.**

GELA (G.S.) – The French National Cancer Institute (INCa).

NCI-SEER – Intramural Research Program of the National Cancer Institute, National Institutes of Health, and Public Health Service (N01-PC-65064,N01-PC-67008, N01-PC-67009, N01-PC-67010, N02-PC-71105). The authors would like to acknowledge the contribution to this study from central cancer registries supported through the Centers for Disease Control and Prevention's National Program of Cancer Registries and cancer registries supported by the National Cancer Institute's Surveillance Epidemiology and End Results Program.

ATBC – The ATBC Study is supported by the Intramural Research Program of the U.S. National Cancer Institute, National Institutes of Health, Department of Health and Human Services.

BCCA – Canadian Institutes for Health Research (CIHR); Canadian Cancer Society; Michael Smith Foundation for Health Research.

CPS-II - The Cancer Prevention Study-II (CPS-II) Nutrition Cohort is supported by the American Cancer Society. Genotyping for all CPS-II samples were supported by the Intramural Research Program of the National Institutes of Health, NCI, Division of Cancer Epidemiology and Genetics. The authors would also like to acknowledge the contribution to this study from central cancer registries supported through the Centers for Disease Control and Prevention National Program of Cancer Registries, and cancer registries supported by the National Cancer Institute Surveillance Epidemiology and End Results program. The authors express sincere appreciation to all Cancer Prevention Study-II participants, and to each member of the study and biospecimen management group.

ELCCS - Blood Cancer UK, United Kingdom.

ENGELA – Association pour la Recherche contre le Cancer (ARC), Institut National du Cancer (INCa), Fondation de France, Fondation contre la Leucémie, Agence nationale de sécurité sanitaire de l'alimentation, de l'environnement et du travail (ANSES)

EPIC – Coordinated Action (Contract #006438, SP23-CT-2005-006438); HuGeF (Human Genetics Foundation), Torino, Italy; Cancer Research UK.

EpiLymph – European Commission (grant references QLK4-CT-2000-00422 and FOOD-CT-2006-023103); the Spanish Ministry of Health (grant references CIBERESP, PI11/01810, PI14/01219, RCESP C03/09, RTICESP C03/10 and RTIC RD06/0020/0095), the Marató de TV3 Foundation (grant reference 051210), the Agència de Gestiód'AjutsUniversitarisi de Recerca – Generalitat de Catalunya (grant reference 2014SRG756) who had no role in the data collection, analysis or interpretation of the results; the NIH (contract NO1-CO-12400); the Compagnia di San Paolo—Programma Oncologia; the Federal Office for Radiation Protection grants StSch4261 and StSch4420, the José Carreras Leukemia Foundation grant DJCLS-R12/23, the German Federal Ministry for Education and Research (BMBF-01-EO-1303); the Health Research Board, Ireland and Cancer Research Ireland; Czech Republic supported by MH CZ – DRO (MMCI, 00209805) and **MEYS - NPS I - LO1413**; Fondation de France and Association de Recherche Contre le Cancer.

HPFS (Walter C. Willet) – The HPFS was supported in part by National Institutes of Health grants UO1 CA167552, R01 CA149445, and R01 CA098122. The content is solely the responsibility of the authors and does not necessarily represent the official views of the National Institutes of Health. The authors would like to acknowledge the contribution to this study from central cancer registries supported through the Centers for Disease Control and Prevention's National Program of Cancer Registries (NPCR) and/or the National Cancer Institute's Surveillance, Epidemiology, and End Results (SEER) Program. Central registries may also be supported by state agencies, universities, and cancer centers. Participating central cancer registries include the following: Alabama, Alaska, Arizona, Arkansas, California, Colorado, Connecticut, Delaware, Florida, Georgia, Hawaii, Idaho, Indiana, Iowa, Kentucky, Louisiana, Massachusetts, Maine, Maryland, Michigan, Mississippi, Montana, Nebraska, Nevada, New Hampshire, New Jersey, New Mexico, New York, North Carolina, North Dakota, Ohio, Oklahoma, Oregon, Pennsylvania, Puerto Rico, Rhode Island, Seattle

SEER Registry, South Carolina, Tennessee, Texas, Utah, Virginia, West Virginia, Wyoming. We would also like to thank the participants and staff of the Health Professionals Follow-up Study for their valuable contributions. The study protocol was approved by the institutional review boards of the Brigham and Women's Hospital and Harvard T.H. Chan School of Public Health, and those of participating registries as required.

IMAGE-UAB This research was supported, in part, by the National Cancer Institute of the National Institutes of Health under Award Numbers: U01 CA249955 and R01 CA300518 (EEB).

Italian GxE - Italian Association for Cancer Research (AIRC, Investigator Grant 11855) (PC); Fondazione Banco di Sardegna 2010-2012, and Regione Autonoma della Sardegna (LR7 CRP-59812/2012) (MGE). The Italian Ministry for Education, University and Research [PRIN 2007 prot. 2007WEJLZB and PRIN 2009 prot. 20092ZELR2]; the Italian Association for Cancer Research [IG 2011/11855] (PC).

MCCS – The Melbourne Collaborative Cohort Study recruitment was funded by VicHealth and Cancer Council Victoria. The MCCS was further supported by Australian NHMRC grants 209057, 396414 and 1074383 and by infrastructure provided by Cancer Council Victoria. Cases and their vital status were ascertained through the Victorian Cancer Registry (VCR) and the Australian Institute of Health and Welfare (AIHW), including the National Death Index and the Australian Cancer Database.

MSKCC – Geoffrey Beene Cancer Research Grant, Lymphoma Foundation (LF5541); Barbara K. Lipman Lymphoma Research Fund (74419); Robert and Kate Niehaus Clinical Cancer Genetics Research Initiative (57470); U01 HG007033; ENCODE; U01 HG007033.

NHS (Meir J. Stampfer, A. Heather Eliassen) – The NHS was supported in part by National Institutes of Health grants UM1 CA186107, P01 CA87969, R01 CA49449, R01 CA149445, R01 CA098122 and R01 CA134958. The content is solely the responsibility of the authors and does not necessarily represent the official views of the National Institutes of Health. The authors would like to acknowledge the contribution to this study from central cancer registries supported through the Centers for Disease Control and Prevention's National Program of Cancer Registries (NPCR) and/or the National Cancer Institute's Surveillance, Epidemiology, and End Results (SEER) Program. Central registries may also be supported by state agencies, universities, and cancer centers. Participating central cancer registries include the following: Alabama, Alaska, Arizona, Arkansas, California, Colorado, Connecticut, Delaware, Florida, Georgia, Hawaii, Idaho, Indiana, Iowa, Kentucky, Louisiana, Massachusetts, Maine, Maryland, Michigan, Mississippi, Montana, Nebraska, Nevada, New Hampshire, New Jersey, New Mexico, New York, North Carolina, North Dakota, Ohio, Oklahoma, Oregon, Pennsylvania, Puerto Rico, Rhode Island, Seattle SEER Registry, South Carolina, Tennessee, Texas, Utah, Virginia, West Virginia, Wyoming. We also thank the participants and staff of the Nurses' Health Study for their valuable contributions. The study protocol was approved by the institutional review boards of the Brigham and Women's Hospital and Harvard T.H. Chan School of Public Health, and those of participating registries as required

NSW - NSW was supported by grants from the Australian National Health and Medical Research Council (ID990920), the Cancer Council NSW, and the University of Sydney Faculty of Medicine.

NYUWHS - National Cancer Institute (P30 CA016087, U01 CA182934).

PLCO - This research was supported by the Intramural Research Program of the National Cancer Institute and by contracts from the Division of Cancer Prevention, National Cancer Institute, NIH, DHHS, and was funded in part with Federal funds from the National Cancer Institute, National Institutes of Health, under NCI Contract No. 75N910D00024. The authors acknowledge the research contributions of the Cancer Genomics Research Laboratory for their expertise, execution, and support of this research in the areas of project planning, wet laboratory processing of specimens, and bioinformatics analysis of generated data. The content of this publication does not necessarily reflect the views or policies of the Department of Health and

Human Services, nor does mention of trade names, commercial products, or organizations imply endorsement by the U.S. Government.

SCALE - Swedish Cancer Society (2009/659). Stockholm County Council (20110209) and the Strategic Research Program in Epidemiology at Karolinska Institutet. Swedish Cancer Society grant (02 6661). National Institutes of Health (5R01 CA69669-02); Plan Denmark. This research was supported, in part, by the National Cancer Institute of the National Institutes of Health under Award Numbers: 5U01CA257679-04 (HH).

UCSF2 – The UCSF studies were supported by the NCI, National Institutes of Health, CA1046282 and CA154643. The collection of cancer incidence data used in this study was supported by the California Department of Health Services as part of the statewide cancer reporting program mandated by California Health and Safety Code Section 103885; the National Cancer Institute's Surveillance, Epidemiology, and End Results Program under contract HHSN261201000140C awarded to the Cancer Prevention Institute of California, contract HHSN261201000035C awarded to the University of Southern California, and contract HHSN261201000034C awarded to the Public Health Institute; and the Centers for Disease Control and Prevention's National Program of Cancer Registries, under agreement #1U58 DP000807-01 awarded to the Public Health Institute. The ideas and opinions expressed herein are those of the authors, and endorsement by the State of California, the California Department of Health Services, the National Cancer Institute, or the Centers for Disease Control and Prevention or their contractors and subcontractors is not intended nor should be inferred.

UTAH/Sheffield - National Cancer Institute (NCI) CA134674. Partial support for data collection at the Utah site was made possible by the Utah Population Database (UPDB) and the Utah Cancer Registry (UCR). Partial support for all datasets within the UPDB is provided by the Huntsman Cancer Institute (HCI) and the HCI Cancer Center Support grant, NCI P30 CA042014. The UCR is funded by the NCI's SEER Program, Contract No. HHSN261201800016I, the US Centers for Disease Control and Prevention's National Program of Cancer Registries, Cooperative Agreement No. NU58DP007131, with additional support from the University of Utah and Huntsman Cancer Foundation. Partial support for data collection in Sheffield, UK was made possible by funds from Yorkshire Cancer Research and the Sheffield Experimental Cancer Medicine Centre. We thank the NCRI Haemato-oncology Clinical Studies Group, colleagues in the North Trent Cancer Network the North Trent Haemato-oncology Database.

WHI – WHI investigators are: *Program Office* - (National Heart, Lung, and Blood Institute, Bethesda, Maryland) Jacques Rossouw, Shari Ludlam, Dale Burwen, Joan McGowan, Leslie Ford, and Nancy Geller; *Clinical Coordinating Center* - (Fred Hutchinson Cancer Research Center, Seattle, WA) Garnet Anderson, Ross Prentice, Andrea LaCroix, and Charles Kooperberg; *Investigators and Academic Centers* - (Brigham and Women's Hospital, Harvard Medical School, Boston, MA) JoAnn E. Manson; (MedStar Health Research Institute/Howard University, Washington, DC) Barbara V. Howard; (Stanford Prevention Research Center, Stanford, CA) Marcia L. Stefanick; (The Ohio State University, Columbus, OH) Rebecca Jackson; (University of Arizona, Tucson/Phoenix, AZ) Cynthia A. Thomson; (University at Buffalo, Buffalo, NY) Jean Wactawski-Wende; (University of Florida, Gainesville/Jacksonville, FL) Marian Limacher; (University of Iowa, Iowa City/Davenport, IA) Robert Wallace; (University of Pittsburgh, Pittsburgh, PA) Lewis Kuller; (Wake Forest University School of Medicine, Winston-Salem, NC) Sally Shumaker; *Women's Health Initiative Memory Study* - (Wake Forest University School of Medicine, Winston-Salem, NC) Sally Shumaker. The WHI program is funded by the National Heart, Lung, and Blood Institute, National Institutes of Health, U.S. Department of Health and Human Services through contracts HHSN268201100046C, HHSN268201100001C, HHSN268201100002C, HHSN268201100003C, HHSN268201100004C, and HHSN271201100004C.

YALE – National Cancer Institute (CA62006); National Cancer Institute (CA165923).

SUPPLEMENTARY TABLE S5. Tests for interactions (likelihood ratio tests for multiplicative interactions) between serum IGF-I and sex, age, height, BMI, alcohol, smoking, and ethnicity in relation to risk of lymphoid neoplasm*

	SEX	BMI (<25,25+), kg/m^2	HEIGHT (sex-specific <med, >=med)	SMOKING (never, ever)	ALCOHOL (never, ever)	ETHNICITY (white, other)	SEX*BMI (<25,25-29,>25)
**LN**	0.08	0.26	0.81	0.98	0.88	0.43	
**NHL**	**0.01**	0.13	0.59	0.87	0.72	0.62	
**B-NHL**	**0.01**	0.11	0.59	0.80	0.53	0.88	
**CLL/SLL**	**0.01**	**0.02**					**0.004**
**MM**	0.54	0.65					

IGF: Insulin-like growth factor; HR: hazard ratio; CI: confidence interval; LN: lymphoid neoplasms; CLL/SLL, chronic lymphocytic leukaemia/small lymphocytic lymphoma; NHL, non-Hodgkin lymphoma; MM, multiple myeloma

* *Tested in "Model2:" adjusted for sex, qualification, UK parts (England/Wales, Scotland), Townsend index (quintiles), BMI (<25, 25-29, >=30, kg/m^2), ethnicity (white, black or black British, other), vigorous PA (0, 1 to 3, and 4 to 7 days/week), height (sex-specific tertiles), alcohol (never, previous, current), smoking (never, past, current), diabetes (yes/no) and fasting (tertiles)

IGF-I examined using 1-SD increase.

SUPPLEMENTARY TABLE S6. Sensitivity analysis: Hazard ratios and 95% CI for the association of serum IGF-I with risk of lymphoid neoplasms, adjusted for C-reactive protein (CRP), testosterone, sex hormone binding globulin (SHBG), and glycosylated haemoglobin (HbA1c),, overall and stratified by sex

Whole cohort:

	Overall								
	Q1	Q2	Q3	Q4		1-SD increase		1-SD increase	
	REF	HR (95% CI)	HR (95% CI)	HR (95% CI)	p-trend	HR (95% CI)	p-valor	corrected HR[a] (95% CI)	test for interaction (sex)
**Lymphoid neoplasms**									
N	971	863	790	697					
model 1	REF	0.98 (0.89 to 1.07)	0.98 (0.89 to 1.07)	0.98 (0.89 to 1.08)	0.66	1.01 (0.98 to 1.05)	0.45	1.02 (0.97 to 1.07)	
model 2	REF	0.98 (0.89 to 1.07)	0.98 (0.89 to 1.08)	0.98 (0.89 to 1.08)	0.66	1.01 (0.98 to 1.05)	0.43	1.02 (0.97 to 1.07)	0.08
model 3 (sensitivity)	REF	1.00 (0.91 to 1.10)	1.01 (0.92 to 1.12)	1.02 (0.92 to 1.13)	0.63	1.03 (1.00 to 1.07)	0.07	1.04 (1.00 to 1.09)	
**MM**									
N	198	172	181	181					
model 1	REF	0.95 (0.78 to 1.17)	1.10 (0.90 to 1.35)	**1.26 (1.02 to 1.55)**	**0.02**	**1.08 (1.01 to 1.16)**	**0.03**	**1.11 (1.01 to 1.22)**	
model 2	REF	0.96 (0.78 to 1.18)	1.11 (0.91 to 1.37)	**1.27 (1.03 to 1.56)**	**0.01**	**1.08 (1.01 to 1.17)**	**0.03**	**1.11 (1.01 to 1.22)**	0.54
model 3 (sensitivity)	REF	0.97 (0.79 to 1.19)	1.12 (0.91 to 1.37)	**1.27 (1.03 to 1.57)**	**0.02**	**1.08 (1.00 to 1.17)**	**0.04**	**1.11 (1.01 to 1.22)**	
**HL**									
N	41	33	28	19					
model 1	REF	0.84 (0.53 to 1.33)	0.74 (0.46 to 1.21)	0.53 (0.30 to 0.92)	0.02	0.83 (0.69 to 1.01)	0.06	0.79 (0.61 to 1.01)	
model 2	REF	0.89 (0.56 to 1.42)	0.80 (0.49 to 1.30)	0.57 (0.32 to 0.99)	0.05	0.86 (0.71 to 1.05)	0.14	0.83 (0.64 to 1.06)	0.92
model 3 (sensitivity)	REF	0.95 (0.60 to 1.52)	0.89 (0.54 to 1.46)	0.67 (0.38 to 1.18)	0.18	0.92 (0.76 to 1.12)	0.43	0.90 (0.70 to 1.16)	
**NHL**									
N	726	644	567	493					
model 1	REF	0.98 (0.88 to 1.08)	0.94 (0.84 to 1.05)	0.93 (0.83 to 1.05)	0.18	1.00 (0.96 to 1.05)	0.89	1.00 (0.95 to 1.06)	
model 2	REF	0.97 (0.87 to 1.08)	0.94 (0.84 to 1.05)	0.93 (0.82 to 1.04)	0.14	1.00 (0.96 to 1.04)	0.97	1.00 (0.95 to 1.06)	**0.01**
model 3 (sensitivity)	REF	1.00 (0.90 to 1.11)	0.97 (0.87 to 1.09)	0.97 (0.86 to 1.10)	0.56	1.02 (0.98 to 1.07)	0.31	1.03 (0.97 to 1.09)	
**B-NHL**									
N	647	557	498	431					
model 1	REF	0.95 (0.85 to 1.06)	0.93 (0.83 to 1.05)	0.92 (0.82 to 1.05)	0.18	1.01 (0.96 to 1.05)	0.72	1.01 (0.95 to 1.07)	
model 2	REF	0.95 (0.85 to 1.06)	0.93 (0.83 to 1.05)	0.92 (0.81 to 1.04)	0.15	1.01 (0.96 to 1.05)	0.79	1.01 (0.95 to 1.07)	**0.01**
model 3 (sensitivity)	REF	0.97 (0.86 to 1.09)	0.96 (0.85 to 1.08)	0.96 (0.84 to 1.09)	0.45	1.02 (0.98 to 1.07)	0.29	1.03 (0.97 to 1.10)	
**CLL/SLL**									
N	217	163	159	128					
model 1	REF	0.84 (0.68 to 1.03)	0.91 (0.74 to 1.12)	0.85 (0.68 to 1.06)	0.21	1.02 (0.95 to 1.11)	0.55	1.03 (0.93 to 1.14)	
model 2	REF	0.84 (0.68 to 1.03)	0.90 (0.73 to 1.11)	0.84 (0.67 to 1.05)	0.18	1.02 (0.94 to 1.11)	0.59	1.03 (0.93 to 1.14)	**0.01**
model 3 (sensitivity)	REF	0.83 (0.67 to 1.02)	0.88 (0.72 to 1.09)	0.81 (0.65 to 1.02)	0.11	1.01 (0.93 to 1.10)	0.78	1.01 (0.91 to 1.13)	
**DLBCL**									
N	194	193	150	143					
model 1	REF	1.11 (0.91 to 1.36)	0.95 (0.77 to 1.18)	1.05 (0.84 to 1.31)	0.98	1.02 (0.94 to 1.10)	0.69	1.02 (0.92 to 1.13)	
model 2	REF	1.12 (0.92 to 1.37)	0.96 (0.78 to 1.20)	1.06 (0.85 to 1.32)	0.96	1.02 (0.94 to 1.10)	0.63	1.03 (0.93 to 1.14)	0.56
model 3 (sensitivity)	REF	1.17 (0.96 to 1.43)	1.03 (0.83 to 1.28)	1.15 (0.92 to 1.44)	0.43	1.05 (0.97 to 1.14)	0.19	1.07 (0.97 to 1.19)	
**FL**									
N	129	113	103	73					
model 1	REF	0.94 (0.73 to 1.21)	0.92 (0.71 to 1.19)	0.72 (0.54 to 0.97)	0.04	0.92 (0.83 to 1.02)	0.11	0.90 (0.78 to 1.02)	
model 2	REF	0.93 (0.72 to 1.20)	0.91 (0.70 to 1.19)	0.72 (0.53 to 0.96)	0.04	0.92 (0.83 to 1.02)	0.10	0.89 (0.78 to 1.02)	0.33
model 3 (sensitivity)	REF	0.96 (0.74 to 1.24)	0.96 (0.73 to 1.25)	0.77 (0.57 to 1.04)	0.13	0.95 (0.85 to 1.05)	0.30	0.93 (0.81 to 1.07)	
**T-NHL**									
N	45	48	39	31					
model 1	REF	1.13 (0.75 to 1.70)	0.99 (0.64 to 1.52)	0.86 (0.54 to 1.38)	0.47	0.96 (0.82 to 1.13)	0.63	0.95 (0.77 to 1.17)	
model 2	REF	1.12 (0.74 to 1.69)	0.98 (0.63 to 1.51)	0.86 (0.54 to 1.37)	0.46	0.96 (0.81 to 1.13)	0.61	0.95 (0.76 to 1.17)	0.27
model 3 (sensitivity)	REF	1.15 (0.76 to 1.73)	1.02 (0.66 to 1.58)	0.92 (0.57 to 1.48)	0.66	0.98 (0.83 to 1.17)	0.86	0.98 (0.79 to 1.22)	

IGF: Insulin-like growth factor; HR: hazard ratio; CI: confidence interval; CLL/SLL, chronic lymphocytic leukaemia/small lymphocytic lymphoma; DLBCL, diffuse large B-cell lymphoma; FL, follicular lymphoma; HL, Hodgkin lymphoma; NHL, non-Hodgkin lymphoma (including MM); MM, multiple myeloma

Model1: adjusted for sex, qualification, UK parts (England/Wales, Scotland), Townsend index (quintiles)

Model2: model 1 + BMI (<25, 25-29, >=30 kg/m^2), ethnicity (white, black or black British, other), vigorous PA, height (sex-specific tertiles), alcohol (never, previous, current), smoking (never, past, current), diabetes(yes/no) and fasting (tertiles). Model 3: model2 + for serum concentrations of C-reactive protein, glycated haemoglobin, sex hormone–binding globulin, and testosterone (sex-specific tertiles, unknown)

IGF-I sex-specific quartiles. [a]: HRs per 1-SD increment were additionally corrected for regression dilution using a regression dilution ratio (0.77) obtained from the subsample of participants with repeat IGF-I measurements

TABLE S6 (continued) Female cohort:

	FEMALE							
	Q1	Q2	Q3	Q4		1-SD increase		1-SD increase
	REF	HR (95% CI)	HR (95% CI)	HR (95% CI)	p-trend	HR (95% CI)	p-valor	corrected HR[a] (95% CI)
**Lymphoid neoplasms**								
N	448	363	344	287				
model 1	REF	0.90 (0.78 to 1.03)	0.94 (0.82 to 1.09)	0.93 (0.80 to 1.08)	0.41	0.97 (0.92 to 1.03)	0.31	0.96 (0.90 to 1.04)
model 2	REF	0.90 (0.79 to 1.04)	0.95 (0.82 to 1.10)	0.93 (0.80 to 1.08)	0.44	0.97 (0.92 to 1.03)	0.34	0.97 (0.90 to 1.04)
model 3 (sensitivity)	REF	0.94 (0.81 to 1.08)	1.01 (0.87 to 1.17)	1.01 (0.86 to 1.18)	0.75	1.01 (0.95 to 1.07)	0.85	1.01 (0.93 to 1.09)
**MM**								
N	92	72	77	**82**				
model 1	REF	0.87 (0.64 to 1.19)	1.05 (0.77 to 1.42)	**1.33 (0.98 to 1.81)**	**0.05**	1.11 (1.00 to 1.25)	0.06	1.15 (1.00 to 1.32)
model 2	REF	0.89 (0.66 to 1.22)	1.08 (0.79 to 1.46)	**1.36 (1.00 to 1.85)**	**0.04**	**1.12 (1.00 to 1.25)**	**0.05**	**1.16 (1.00 to 1.34)**
model 3 (sensitivity)	REF	0.89 (0.65 to 1.22)	1.08 (0.79 to 1.47)	**1.36 (0.98 to 1.87)**	**0.05**	1.12 (1.00 to 1.26)	0.06	1.16 (1.00 to 1.34)
**HL**								
N	18	14	14	7				
model 1	REF	0.78 (0.39 to 1.58)	0.79 (0.39 to 1.61)	0.39 (0.16 to 0.97)	0.06	0.80 (0.59 to 1.08)	0.14	0.74 (0.50 to 1.10)
model 2	REF	0.78 (0.39 to 1.58)	0.77 (0.38 to 1.59)	0.38 (0.15 to 0.95)	0.05	0.79 (0.58 to 1.06)	0.12	0.73 (0.49 to 1.08)
model 3 (sensitivity)	REF	0.86 (0.42 to 1.75)	0.93 (0.45 to 1.93)	0.51 (0.20 to 1.29)	0.23	0.89 (0.65 to 1.21)	0.45	0.85 (0.57 to 1.29)
**NHL**								
N	335	273	249	195				
model 1	REF	0.90 (0.77 to 1.06)	0.92 (0.78 to 1.08)	0.85 (0.71 to 1.02)	0.10	0.94 (0.88 to 1.00)	0.06	0.92 (0.84 to 1.00)
model 2	REF	0.91 (0.77 to 1.06)	0.92 (0.78 to 1.09)	0.85 (0.71 to 1.02)	0.10	0.94 (0.88 to 1.00)	0.06	0.92 (0.84 to 1.00)
model 3 (sensitivity)	REF	0.94 (0.80 to 1.11)	0.98 (0.83 to 1.16)	0.93 (0.77 to 1.12)	0.53	0.97 (0.91 to 1.04)	0.43	0.96 (0.88 to 1.06)
**B-NHL**								
N	296	241	221	164				
model 1	REF	0.91 (0.76 to 1.08)	0.93 (0.78 to 1.11)	0.82 (0.68 to 1.00)	0.08	0.94 (0.87 to 1.01)	0.08	0.92 (0.84 to 1.01)
model 2	REF	0.91 (0.77 to 1.08)	0.94 (0.78 to 1.12)	0.83 (0.68 to 1.01)	0.09	0.94 (0.88 to 1.01)	0.09	0.92 (0.84 to 1.01)
model 3 (sensitivity)	REF	0.95 (0.80 to 1.13)	1.00 (0.83 to 1.20)	0.90 (0.74 to 1.11)	0.47	0.98 (0.91 to 1.05)	0.53	0.97 (0.88 to 1.07)
**CLL/SLL**								
N	89	71	59	42				
model 1	REF	0.90 (0.66 to 1.23)	0.85 (0.61 to 1.19)	0.75 (0.52 to 1.10)	0.13	0.90 (0.79 to 1.03)	0.12	0.87 (0.73 to 1.04)
model 2	REF	0.90 (0.66 to 1.23)	0.85 (0.61 to 1.19)	0.75 (0.52 to 1.10)	0.13	0.90 (0.79 to 1.03)	0.12	0.87 (0.73 to 1.04)
model 3 (sensitivity)	REF	0.90 (0.65 to 1.23)	0.85 (0.60 to 1.19)	0.74 (0.50 to 1.10)	0.13	0.89 (0.78 to 1.03)	0.12	0.86 (0.71 to 1.04)
**DLBCL**								
N	94	84	72	61				
model 1	REF	1.01 (0.75 to 1.36)	0.98 (0.72 to 1.34)	1.01 (0.72 to 1.40)	0.98	0.99 (0.88 to 1.11)	0.85	0.98 (0.84 to 1.15)
model 2	REF	1.03 (0.77 to 1.39)	1.01 (0.74 to 1.38)	1.03 (0.74 to 1.44)	0.89	1.00 (0.89 to 1.13)	0.99	1.00 (0.85 to 1.17)
model 3 (sensitivity)	REF	1.09 (0.81 to 1.47)	1.10 (0.80 to 1.51)	1.16 (0.82 to 1.64)	0.40	1.05 (0.93 to 1.19)	0.46	1.06 (0.90 to 1.25)
**FL**								
N	72	62	54	35				
model 1	REF	0.93 (0.66 to 1.31)	0.88 (0.62 to 1.26)	**0.65 (0.43 to 0.98)**	**0.05**	0.88 (0.76 to 1.02)	0.08	0.85 (0.70 to 1.02)
model 2	REF	0.94 (0.66 to 1.32)	0.88 (0.62 to 1.27)	**0.65 (0.43 to 0.99)**	**0.05**	0.88 (0.76 to 1.02)	0.09	0.85 (0.70 to 1.02)
model 3 (sensitivity)	REF	0.98 (0.70 to 1.39)	0.96 (0.67 to 1.39)	0.73 (0.48 to 1.13)	0.21	0.93 (0.80 to 1.08)	0.33	0.91 (0.74 to 1.10)
**T-NHL**								
N	25	14	15	14				
model 1	REF	0.59 (0.30 to 1.13)	0.67 (0.35 to 1.27)	0.70 (0.36 to 1.37)	0.29	0.85 (0.65 to 1.10)	0.21	0.80 (0.57 to 1.13)
model 2	REF	0.57 (0.30 to 1.10)	0.64 (0.33 to 1.23)	0.67 (0.34 to 1.31)	0.24	0.83 (0.64 to 1.08)	0.17	0.78 (0.55 to 1.11)
model 3 (sensitivity)	REF	0.54 (0.28 to 1.05)	0.59 (0.30 to 1.15)	0.60 (0.29 to 1.21)	0.15	0.79 (0.59 to 1.04)	0.09	0.73 (0.50 to 1.05)

IGF: Insulin-like growth factor; HR: hazard ratio; CI: confidence interval; CLL/SLL, chronic lymphocytic leukaemia/small lymphocytic lymphoma; DLBCL, diffuse large B-cell lymphoma; FL, follicular lymphoma; HL, Hodgkin lymphoma; NHL, non-Hodgkin lymphoma (including MM); MM, multiple myeloma

Model1: adjusted for sex, qualification, UK parts (England/Wales, Scotland), Townsend index (quintiles)

Model2: model 1 + BMI (<25, 25-29, >=30 kg/m^2), ethnicity (white, black or black British, other), vigorous PA, height (sex-specific tertiles), alcohol (never, previous, current), smoking(never, past, current), diabetes(yes/no) and fasting (tertiles). Model 3: model2 + for serum concentrations of C-reactive protein, glycated haemoglobin, sex hormone–binding globulin, and testosterone (sex-specific tertiles, unknown)

IGF-I sex-specific quartiles. [a]: HRs per 1-SD increment were additionally corrected for regression dilution using a regression dilution ratio (0.77) obtained from the subsample of participants with repeat IGF-I measurements

TABLE S6 (continued) Male cohort:

	MALE							
	Q1	Q2	Q3	Q4		1-SD increase		1-SD increase
	REF	HR (95% CI)	HR (95% CI)	HR (95% CI)	p-trend	HR (95% CI)	p-value	corrected HR[a] (95% CI)
**Lymphoid neoplasms**								
N	523	500	446	410				
model 1	REF	1.04 (0.92 to 1.18)	1.00 (0.88 to 1.14)	1.02 (0.89 to 1.16)	0.93	1.04 (1.00 to 1.09)	0.07	1.06 (1.00 to 1.12)
model 2	REF	1.04 (0.92 to 1.18)	1.00 (0.88 to 1.13)	1.01 (0.88 to 1.15)	0.95	1.04 (0.99 to 1.09)	0.10	1.05 (0.99 to 1.12)
model 3 (sensitivity)	REF	1.05 (0.93 to 1.19)	1.02 (0.89 to 1.16)	1.04 (0.91 to 1.19)	0.73	**1.05 (1.00 to 1.11)**	**0.03**	**1.07 (1.01 to 1.14)**
**MM**								
N	106	100	104	99				
model 1	REF	1.02 (0.78 to 1.34)	1.15 (0.87 to 1.50)	1.21 (0.91 to 1.59)	0.13	1.06 (0.96 to 1.17)	0.26	1.08 (0.95 to 1.22)
model 2	REF	1.02 (0.77 to 1.34)	1.13 (0.86 to 1.49)	1.18 (0.89 to 1.56)	0.19	1.05 (0.95 to 1.15)	0.36	1.06 (0.93 to 1.21)
model 3 (sensitivity)	REF	1.02 (0.77 to 1.34)	1.14 (0.86 to 1.50)	1.18 (0.89 to 1.57)	0.18	1.05 (0.95 to 1.16)	0.36	1.06 (0.93 to 1.21)
**HL**								
N	23	19	14	12				
model 1	REF	0.89 (0.48 to 1.64)	0.70 (0.36 to 1.37)	0.64 (0.31 to 1.30)	0.16	0.85 (0.66 to 1.10)	0.23	0.82 (0.59 to 1.14)
model 2	REF	0.99 (0.54 to 1.84)	0.78 (0.40 to 1.53)	0.71 (0.35 to 1.45)	0.28	0.91 (0.71 to 1.16)	0.44	0.88 (0.64 to 1.21)
model 3 (sensitivity)	REF	1.06 (0.57 to 1.96)	0.86 (0.44 to 1.71)	0.81 (0.39 to 1.68)	0.49	0.96 (0.75 to 1.23)	0.74	0.95 (0.68 to 1.31)
**NHL**								
N	391	371	318	298				
model 1	REF	1.04 (0.90 to 1.20)	0.96 (0.83 to 1.11)	1.00 (0.85 to 1.16)	0.71	1.05 (0.99 to 1.11)	0.08	1.06 (0.99 to 1.14)
model 2	REF	1.03 (0.89 to 1.19)	0.95 (0.82 to 1.10)	0.98 (0.84 to 1.14)	0.58	1.05 (0.99 to 1.11)	0.11	1.06 (0.99 to 1.13)
model 3 (sensitivity)	REF	1.04 (0.90 to 1.21)	0.97 (0.83 to 1.13)	1.01 (0.86 to 1.18)	0.85	1.06 (1.00 to 1.12)	**0.04**	**1.08 (1.00 to 1.16)**
**B-NHL**								
N	351	316	277	267				
model 1	REF	0.99 (0.85 to 1.15)	0.93 (0.80 to 1.10)	1.00 (0.85 to 1.18)	0.81	**1.06 (1.00 to 1.12)**	**0.05**	**1.08 (1.00 to 1.16)**
model 2	REF	0.98 (0.84 to 1.14)	0.92 (0.79 to 1.08)	0.98 (0.84 to 1.16)	0.64	1.05 (0.99 to 1.11)	0.08	1.07 (0.99 to 1.15)
model 3 (sensitivity)	REF	0.99 (0.85 to 1.15)	0.93 (0.80 to 1.10)	1.00 (0.85 to 1.18)	0.78	**1.06 (1.00 to 1.12)**	**0.05**	**1.08 (1.00 to 1.16)**
**CLL/SLL**								
N	128	92	100	86				
model 1	REF	0.80 (0.61 to 1.04)	0.94 (0.72 to 1.23)	0.91 (0.69 to 1.20)	0.71	**1.10 (1.00 to 1.21)**	**0.05**	**1.13 (1.00 to 1.28)**
model 2	REF	0.80 (0.61 to 1.05)	0.94 (0.72 to 1.22)	0.90 (0.68 to 1.19)	0.65	**1.10 (1.00 to 1.21)**	**0.05**	**1.13 (1.00 to 1.28)**
model 3 (sensitivity)	REF	0.79 (0.60 to 1.03)	0.91 (0.70 to 1.2)	0.87 (0.65 to 1.15)	0.48	1.09 (0.99 to 1.20)	0.09	1.11 (0.98 to 1.26)
**DLBCL**								
N	100	109	78	82				
model 1	REF	1.20 (0.91 to 1.58)	0.93 (0.69 to 1.25)	1.09 (0.81 to 1.47)	1.00	1.04 (0.94 to 1.15)	0.47	1.05 (0.92 to 1.20)
model 2	REF	1.20 (0.91 to 1.58)	0.92 (0.68 to 1.25)	1.08 (0.80 to 1.45)	0.93	1.03 (0.93 to 1.15)	0.52	1.04 (0.91 to 1.20)
model 3 (sensitivity)	REF	1.24 (0.94 to 1.63)	0.97 (0.72 to 1.31)	1.14 (0.84 to 1.55)	0.75	1.06 (0.95 to 1.18)	0.28	1.08 (0.94 to 1.24)
**FL**								
N	57	51	49	38				
model 1	REF	0.95 (0.65 to 1.38)	0.96 (0.65 to 1.41)	0.80 (0.53 to 1.22)	0.36	0.96 (0.83 to 1.12)	0.62	0.95 (0.79 to 1.15)
model 2	REF	0.93 (0.63 to 1.36)	0.94 (0.64 to 1.39)	0.79 (0.52 to 1.20)	0.32	0.96 (0.82 to 1.11)	0.56	0.94 (0.78 to 1.15)
model 3 (sensitivity)	REF	0.94 (0.64 to 1.37)	0.96 (0.65 to 1.42)	0.83 (0.54 to 1.27)	0.44	0.98 (0.84 to 1.14)	0.75	0.97 (0.79 to 1.18)
**T-NHL**								
N	20	34	24	17				
model 1	REF	1.83 (1.05 to 3.18)	1.38 (0.76 to 2.5)	1.06 (0.55 to 2.03)	0.92	1.04 (0.84 to 1.28)	0.71	1.05 (0.81 to 1.37)
model 2	REF	1.82 (1.04 to 3.17)	1.38 (0.76 to 2.52)	1.07 (0.55 to 2.06)	0.95	1.04 (0.85 to 1.28)	0.68	1.06 (0.81 to 1.38)
model 3 (sensitivity)	REF	1.90 (1.09 to 3.32)	1.50 (0.82 to 2.74)	1.21 (0.62 to 2.36)	0.72	1.10 (0.89 to 1.35)	0.37	1.13 (0.86 to 1.48)

IGF: Insulin-like growth factor; HR: hazard ratio; CI: confidence interval; CLL/SLL, chronic lymphocytic leukaemia/small lymphocytic lymphoma; DLBCL, diffuse large B-cell lymphoma; FL, follicular lymphoma; HL, Hodgkin lymphoma; NHL, non-Hodgkin lymphoma (including MM); MM, multiple myeloma

Model1: adjusted for sex, qualification, UK parts (England/Wales, Scotland), Townsend index (quintiles)

Model2: model 1 + BMI (<25, 25-29, >=30 kg/m^2), ethnicity (white, black or black British, other), vigorous PA, height (sex-specific tertiles), alcohol (never, previous, current), smoking (never, past, current), diabetes(yes/no) and fasting (tertiles). Model 3: model2 + for serum concentrations of C-reactive protein, glycated haemoglobin, sex hormone–binding globulin, and testosterone (sex-specific tertiles, unknown)

IGF-I sex-specific quartiles. [a]: HRs per 1-SD increment were additionally corrected for regression dilution using a regression dilution ratio (0.77) obtained from the subsample of participants with repeat IGF-I measurements

SUPPLEMENTARY TABLE S7. Sensitivity analysis: Hazard ratios and 95% CI for the association of serum IGF-I with risk of multiple myeloma, excluding the first two years of follow-up, and with complete data analysis, overall and stratified by sex

	Q1	Q2	Q3	Q4		1-SD increase		1-SD increase
	REF	HR (95% CI)	HR (95% CI)	HR (95% CI)	p-trend	HR (95% CI)	p-value	corrected HR[a]
**OVERALL**								
n	198	172	181	181		723		723
model 1	REF	0.95 (0.78 to 1.17)	1.10 (0.90 to 1.35)	**1.26 (1.02 to 1.55)**	**0.02**	**1.08 (1.01 to 1.16)**	**0.03**	**1.11 (1.01 to 1.22)**
model 2	REF	0.96 (0.78 to 1.18)	1.11 (0.91 to 1.37)	**1.27 (1.03 to 1.56)**	**0.01**	**1.08 (1.01 to 1.17)**	**0.03**	**1.11 (1.01 to 1.22)**
Exclusion of the first two years of follow-up								
n	173	159	159	169		660		660
model 2	REF	1.02 (0.82 to 1.26)	1.11 (0.89 to 1.38)	**1.34 (1.08 to 1.66)**	**0.007**	**1.10 (1.01 to 1.18)**	**0.02**	**1.13 (1.02 to 1.24)**
Complete data analysis								
n	182	155	171	168		676		676
model 2	REF	0.92 (0.74 to 1.14)	1.11 (0.90 to 1.37)	**1.23 (0.99 to 1.53)**	**0.03**	1.07 (0.99 to 1.16)	0.07	1.10 (0.99 to 1.21)
**FEMALE**								
n	92	72	77	82		323		323
model 1	REF	0.87 (0.64 to 1.19)	1.05 (0.77 to 1.42)	**1.33 (0.98 to 1.81)**	**0.05**	1.11 (1.00 to 1.25)	0.06	1.15 (1.00 to 1.32)
model 2	REF	0.89 (0.66 to 1.22)	1.08 (0.79 to 1.46)	**1.36 (1.00 to 1.85)**	**0.04**	**1.12 (1.00 to 1.25)**	**0.05**	**1.16 (1.00 to 1.34)**
Exclusion of the first two years of follow-up:								
MM, n	84	63	70	78		295		295
model 2	REF	0.85 (0.61 to 1.18)	1.05 (0.76 to 1.45)	**1.38 (1.00 to 1.90)**	**0.04**	**1.13 (1.01 to 1.27)**	**0.04**	**1.17 (1.01 to 1.36)**
Complete data analysis								
N	82	63	72	78		295		295
model 2	REF	0.85 (0.61 to 1.19)	1.09 (0.79 to 1.5)	**1.39 (1.00 to 1.91)**	**0.03**	**1.13 (1.01 to 1.27)**	**0.04**	**1.17 (1.01 to 1.36)**
**MALE**								
n	106	100	104	99		409		409
model 1	REF	1.02 (0.78 to 1.34)	1.15 (0.87 to 1.50)	1.21 (0.91 to 1.59)	0.13	1.06 (0.96 to 1.17)	0.26	1.08 (0.95 to 1.22)
model 2	REF	1.02 (0.77 to 1.34)	1.13 (0.86 to 1.49)	1.18 (0.89 to 1.56)	0.19	1.05 (0.95 to 1.15)	0.36	1.06 (0.93 to 1.21)
Exclusion of the first two years of follow-up								
n	89	96	89	91		365		365
model 2	REF	1.16 (0.87 to 1.55)	1.15 (0.85 to 1.55)	1.28 (0.95 to 1.73)	0.13	1.02 (0.92 to 1.13)	0.29	1.03 (0.90 to 1.18)
Complete data analysis								
n	100	92	99	90		381		381
model 2	REF	0.97 (0.73 to 1.28)	1.11 (0.83 to 1.46)	1.10 (0.82 to 1.47)	0.37	1.02 (0.92 to 1.13)	0.69	1.03 (0.90 to 1.18)

IGF: Insulin-like growth factor; HR: hazard ratio; CI: confidence interval; IGF-I sex-specific quartiles; n: number.
Model[1]: adjusted for sex, qualification, UK parts (England/Wales, Scotland), Townsend index (quintiles)
Model2: model 1 + BMI (<25, 25-29, >=30 kg/m^2), ethnicity (white, black or black British, other), vigorous PA, height (sex-specific tertiles), alcohol (never, previous, current), smoking (never, past, current), diabetes (yes/no) and fasting (tertiles)
[a]: HRs per 1-SD increment were additionally corrected for regression dilution using a regression dilution ratio (0.77) obtained from the subsample of participants with repeat IGF-I measurements

SUPPLEMENTARY TABLE S8. Sensitivity analysis: Hazard ratios (HR) and 95% confidence intervals (CI) for risk of chronic lymphocytic leukaemia (CLL/SLL) per 1-SD increase in circulating IGF-I, overall and jointly by sex and body mass index (BMI) at enrolment, following exclusion of participants in the highest and lowest values, 1% of the distribution (n= 4,442)[a]

	Female		Male		Overall	
**body mass index (kg/m²)**	n	HR[b] (95% CI)	n	HR[b] (95% CI)	n	HR[b] (95% CI)
**<25**	103	0.94 (0.72 to 1.25)	85	0.83 (0.62 to 1.12)	188	0.88 (0.72 to 1.08)
**25-29**	92	0.91 (0.68 to 1.21)	216	1.02 (0.85 to 1.22)	308	0.98 (0.84 to 1.15)
**≥30**	65	0.75 (0.53 to 1.07)	99	**1.28 (1.00 to 1.63)**	164	1.06 (0.86 to 1.29)

P-value for two-way interactions for IGF-I and sex= 0.07
P-value for two-way interactions for IGF-I and body mass index= 0.11
P-value for three-way interactions for IGF-I, sex and BMI= 0.05
IGF: Insulin-like growth factor [a]: IGF-I mean(SD)/min/max (nmol/L): a) for the whole cohort (n = 444,187): 21.4 (5.7) / 1.4 / 126.8; b) for the restricted cohort (n = 439,745): 21.4 (5.3) / 8.6 / 39.0; c) for patients with CLL/SLL of the whole cohort (n = 667): 20.9 (7.0) / 6.3 / 105.7; d) for patients with CLL/SLL of the restricted cohort (n = 661): 20.7 (5.5) / 9.3 / 39.0.
[b]: HRs per 1-SD increment were corrected for a regression dilution ratio (0.77) obtained from the subsample of 17,697 participants with repeat IGF-I measurements. Model adjusted for sex, qualification, UK parts (England/Wales, Scotland), Townsend index (quintiles), ethnicity (white, black or black British, other), vigorous PA, height (sex-specific tertiles), alcohol (never, previous, current), smoking (never, past, current), diabetes (yes/no) and fasting (tertiles).

SUPPLEMENTARY FIGURE S9. Scatter plot of SNP-IGF-I vs SNP-MM associations

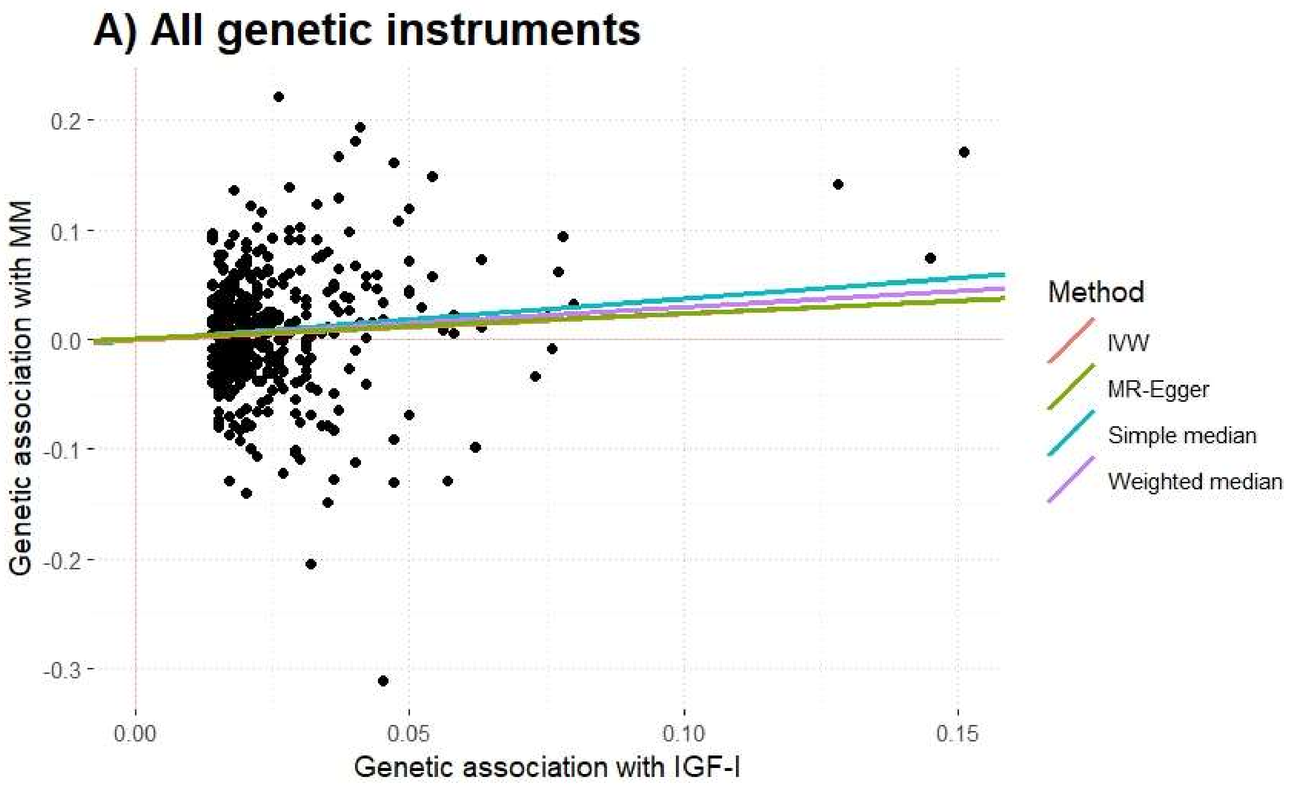

SUPPLEMENTARY TABLE S10. Comparison of published and current results from prospective studies on the relation between IGF-I levels and multiple myeloma (MM)

			tertiles (T) or quartiles (Q)					per unit increase		
			T1/Q1	T2/Q2	T3/Q3	Q4				
Studies, year, reference	N	type[1]		HR/OR (95% CI)	HR/OR (95% CI)	HR/OR (95% CI)	p-trend	HR/OR (95% CI)	unit (nmol/L)	p-trend
MMCC, 2012,[8]	493	NCC	REF	1.0 (0.7 to 1.3)	0.8 (0.6 to 1.1)	1.3 (0.9 to 1.8)	0.22	1.1 (1.0 to 1.3)	1-SD*~ 6.4	0.07
EPIC, 2017,[9]	237	NCC	REF	0.8 (0.5 to 1.2)	1.0 (0.6 to 1.6)	NA	0.6	NA	NA	NA
UK Biobank, 2021,[10]	367	C	NA	NA	NA	NA	NA	1.13 (1.01 to 1.27)	5.0	<0.05
UK Biobank, Current	732	C	REF	0.96 (0.78 to 1.18)	1.11 (0.91 to 1.37)	1.27 (1.03 to 1.56)	0.01	1.11 (1.01 to 1.22)	1-SD: 5.7<br>5.5 (M)<br>5.8 (F)	0.03

IGF: Insulin-like growth factor. MMCC: the Multiple Myeloma Cohort Consortium; EPIC: the European Prospective Investigation into Cancer and Nutrition; [1]: NCC: nested case-control study; C: cohort; N: number of MM cases; OR: Odds ratios; HR: Hazard ratio; OR: Odds ratios; T: tertile; Q: quartile; OR: odds ratio, NA: not available; SD: standard deviation; M: male; F: female. *: 48.85 ng/mL.

References

1. Clarke R, Shipley M, Lewington S, et al. Underestimation of risk associations due to regression dilution in long-term follow-up of prospective studies. American journal of epidemiology 1999;150(4):341–353.
2. Elliott P, Peakman TC. The UK Biobank sample handling and storage protocol for the collection, processing and archiving of human blood and urine. International Journal of Epidemiology [Epub ahead of print].
3. Murphy N, Carreras-Torres R, Song M, et al. Circulating Levels of Insulin-like Growth Factor 1 and Insulin-like Growth Factor Binding Protein 3 Associate With Risk of Colorectal Cancer Based on Serologic and Mendelian Randomization Analyses. Gastroenterology 2020;158(5):1300-1312.e20.
4. Bowden J, Davey Smith G, Haycock PC, Burgess S. Consistent Estimation in Mendelian Randomization with Some Invalid Instruments Using a Weighted Median Estimator. Genetic epidemiology 2016;40(4):304–314.
5. Bowden J, Smith GD, Burgess S. Mendelian randomization with invalid instruments: effect estimation and bias detection through Egger regression. International journal of epidemiology 2015;44(2):512–525.
6. Verbanck M, Chen CY, Neale B, Do R. Detection of widespread horizontal pleiotropy in causal relationships inferred from Mendelian randomization between complex traits and diseases. Nature Genetics 2018 50:5 2018;50(5):693–698.
7. Burgess S, Thompson SG. Avoiding bias from weak instruments in Mendelian randomization studies. International Journal of Epidemiology 2011;40(3):755–764.
8. Birmann BM, Neuhouser ML, Rosner B, et al. Prediagnosis biomarkers of insulin-like growth factor-1, insulin, and interleukin-6 dysregulation and multiple myeloma risk in the Multiple Myeloma Cohort Consortium. Blood. 2012 Dec 13;120(25):4929-37.
9. Perez-Cornago A, Appleby PN, Tipper S, et al. Prediagnostic circulating concentrations of plasma insulin-like growth factor-I and risk of lymphoma in the European Prospective Investigation into Cancer and Nutrition. Int J Cancer. 2017 Mar 1;140(5):1111-1118.
10. Knuppel A, Fensom GK, Watts EL, et al. Circulating insulin-like growth factor-I concentrations and risk of 30 cancers: Prospective analyses in UK biobank. Cancer Research 2021;80(18):4014–4021.
